# Multiomics profiling of primary lung cancers and distant metastases reveals immunosuppression as a common characteristic of tumor cells with metastatic plasticity

**DOI:** 10.1186/s13059-020-02175-0

**Published:** 2020-11-04

**Authors:** Won-Chul Lee, Alexandre Reuben, Xin Hu, Nicholas McGranahan, Runzhe Chen, Ali Jalali, Marcelo V. Negrao, Shawna M. Hubert, Chad Tang, Chia-Chin Wu, Anthony San Lucas, Whijae Roh, Kenichi Suda, Jihye Kim, Aik-Choon Tan, David H. Peng, Wei Lu, Ximing Tang, Chi-Wan Chow, Junya Fujimoto, Carmen Behrens, Neda Kalhor, Kazutaka Fukumura, Marcus Coyle, Rebecca Thornton, Curtis Gumbs, Jun Li, Chang-Jiun Wu, Latasha Little, Emily Roarty, Xingzhi Song, J. Jack Lee, Erik P. Sulman, Ganesh Rao, Stephen Swisher, Lixia Diao, Jing Wang, John V. Heymach, Jason T. Huse, Paul Scheet, Ignacio I. Wistuba, Don L. Gibbons, P. Andrew Futreal, Jianhua Zhang, Daniel Gomez, Jianjun Zhang

**Affiliations:** 1 Department of Genomic Medicine, The University of Texas MD Anderson Cancer Center, Houston, TX USA; 2 Department of Thoracic/Head and Neck Medical Oncology, The University of Texas MD Anderson Cancer Center, Houston, TX USA; 3 Cancer Research UK Lung Cancer Centre of Excellence, University College London Cancer Institute, London, UK; 4 Department of Neurosurgery, Baylor College of Medicine, Houston, TX USA; 5 Department of Radiation Oncology, The University of Texas MD Anderson Cancer Center, Houston, TX USA; 6 Department of Epidemiology, The University of Texas MD Anderson Cancer Center, Houston, TX USA; 7 Broad Institute of Harvard and Massachusetts Institute of Technology, Cambridge, MA USA; 8 Department of Thoracic Surgery, Kindai University Faculty of Medicine, Osaka-Sayama, Japan; 9 Division of Medical Oncology, University of Colorado Anschutz Medical Campus, Aurora, CO USA; 10 Department of Biostatistics and Bioinformatics, Moffitt Cancer Center, Tampa, FL USA; 11 NYU Langone Health, New York, NY USA; 12 Department of Translational Molecular Pathology, The University of Texas MD Anderson Cancer Center, Houston, TX USA; 13 Department of Pathology, The University of Texas MD Anderson Cancer Center, Houston, TX USA; 14 Department of Biostatistics, The University of Texas MD Anderson Cancer Center, Houston, TX USA; 15 New York University Langone School of Medicine, New York, NY USA; 16 Department of Neurosurgery, The University of Texas MD Anderson Cancer Center, Houston, TX USA; 17 Department of Thoracic Surgery, The University of Texas MD Anderson Cancer Center, Houston, TX USA; 18 Department of Bioinformatics and Computational Biology, The University of Texas MD Anderson Cancer Center, Houston, TX USA; 19 Current Address: Department of Radiation Oncology, Memorial Sloan Kettering Cancer Center, New York, NY USA

**Keywords:** Lung cancer, Metastasis, Multiomics, Immune profiling, Genomics, DNA methylation, Gene expression

## Abstract

**Background:**

Metastasis is the primary cause of cancer mortality accounting for 90% of cancer deaths. Our understanding of the molecular mechanisms driving metastasis is rudimentary.

**Results:**

We perform whole exome sequencing (WES), RNA sequencing, methylation microarray, and immunohistochemistry (IHC) on 8 pairs of non-small cell lung cancer (NSCLC) primary tumors and matched distant metastases. Furthermore, we analyze published WES data from 35 primary NSCLC and metastasis pairs, and transcriptomic data from 4 autopsy cases with metastatic NSCLC and one metastatic lung cancer mouse model. The majority of somatic mutations are shared between primary tumors and paired distant metastases although mutational signatures suggest different mutagenesis processes in play before and after metastatic spread. Subclonal analysis reveals evidence of monoclonal seeding in 41 of 42 patients. Pathway analysis of transcriptomic data reveals that downregulated pathways in metastases are mainly immune-related. Further deconvolution analysis reveals significantly lower infiltration of various immune cell types in metastases with the exception of CD4+ T cells and M2 macrophages. These results are in line with lower densities of immune cells and higher CD4/CD8 ratios in metastases shown by IHC. Analysis of transcriptomic data from autopsy cases and animal models confirms that immunosuppression is also present in extracranial metastases. Significantly higher somatic copy number aberration and allelic imbalance burdens are identified in metastases.

**Conclusions:**

Metastasis is a molecularly late event, and immunosuppression driven by different molecular events, including somatic copy number aberration, may be a common characteristic of tumors with metastatic plasticity.

## Background

Metastasis, a process of cancer cells spreading from the primary tumor to distant organs, is the primary cause of cancer mortality. It is estimated that metastasis is responsible for 90% of cancer deaths [1] and this has remained true over the past half century [2]. Understanding the mechanisms underlying metastasis is critical to identify biomarkers and novel therapeutic targets and eventually improve patient outcomes. Clinically, metastasis equals late-stage cancer, but when (molecular time) and how (underlying mechanism and mode of seeding) metastasis occurs is largely unknown.

Cancer spread, including distant metastasis, is thought to result from accumulation of somatic mutations followed by selection of the fittest clones, eventually giving rise to metastatic spread [3–6]. Comparative studies on paired primary tumors and metastases have the potential to identify molecular changes associated with the development of metastasis. Using this approach, several studies have revealed a varying degree of genetic divergence between primary tumors and metastases [7–11]. However, the majority of previous studies have focused on the somatic mutations of primary tumors and metastases. Other molecular changes such as somatic copy number aberrations (SCNAs), epigenetic and gene expression alterations, and tumor microenvironment, particularly immune contexture, can play important roles in the metastatic cascade [12–15]. With the intent to comprehensively depict the difference in the molecular and immune landscapes between primary NSCLC tumors and metastases, we performed multiomics profiling of 8 pairs of NSCLC primary tumors, matched distant metastases, and tumor-adjacent morphologically normal tissues. Specifically, all 8 patients underwent whole exome sequencing (WES) and RNA sequencing (RNA-seq); 7 were subjected to methylation microarray, and 5 were assessed for T cell profile by immunohistochemistry (IHC) with multiple T cell markers (Additional file 1: Table S1). In addition, we re-analyzed previously published WES data from 35 pairs of primary NSCLC tumors and matched brain metastases (Brastianos cohort) [11] and RNA-seq data from 4 patients with extensive metastatic NSCLC [16] and from a metastatic lung cancer mouse model [17] and compared these results to intratumor heterogeneity (ITH) data in primary NSCLC tumors from the TRACERx dataset [18].

## Results

### Metastasis is a molecularly late event following the clonal expansion model

**Fig. 1:**
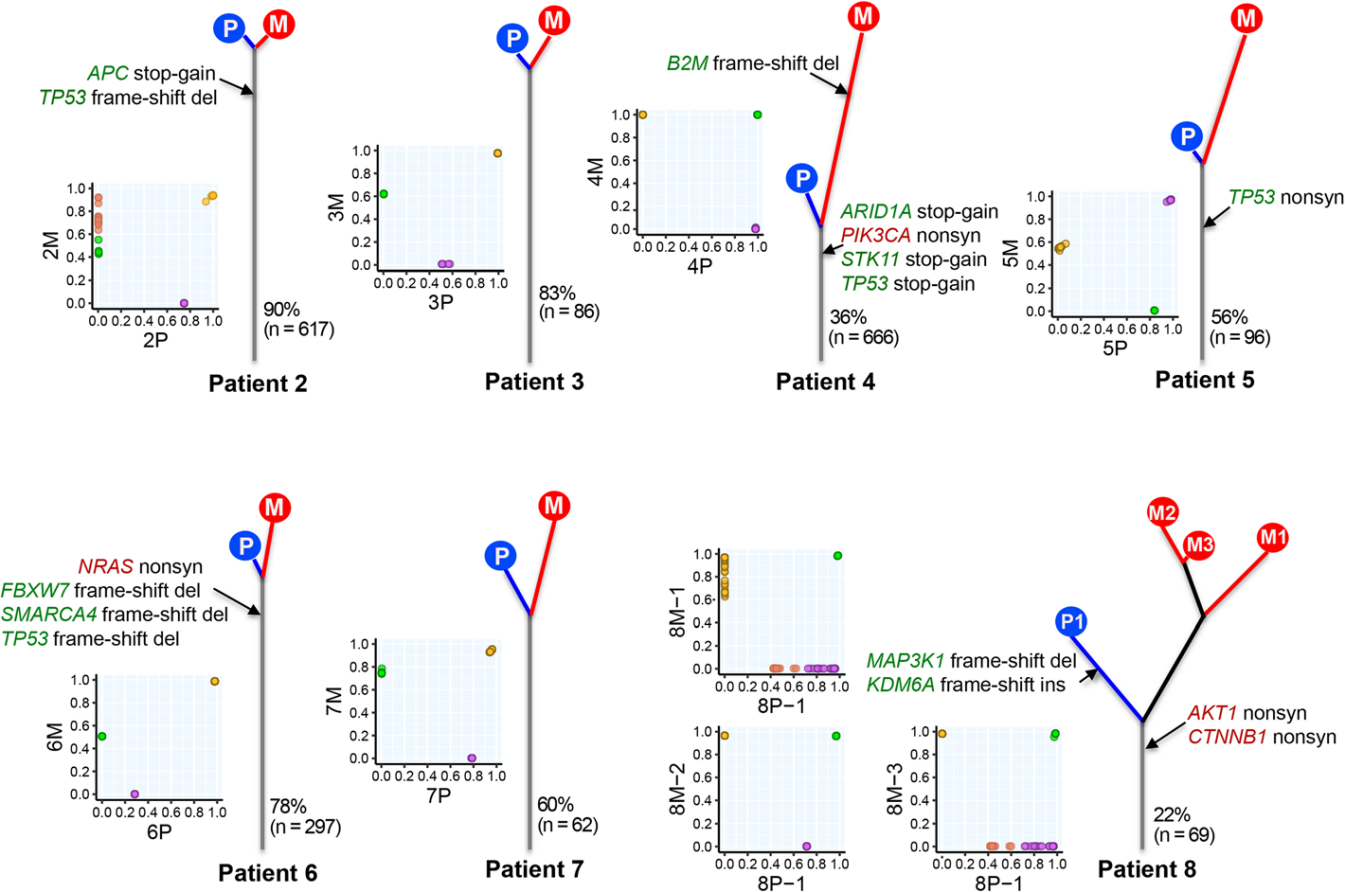
Genetic divergence of primary lung tumors and paired distant metastases. For each patient, the left scatter plot shows cancer cell fraction (CCF) values for somatic single nucleotide variants. Variants with different color show the different clones. In the phylogenetic trees of primary (P) and metastatic (M) tumors, trunk and branch lengths are proportional to the number of somatic mutations. Cancer gene mutations are displayed with the trees (oncogene in red and tumor suppressor gene in green)

We first investigated the difference of somatic mutations between primary tumors and matched distant metastases in the 7 patients with available paired germline DNA. Despite a wide span (3 to 24 months, a median of 9 months) and different therapies between resection of primary tumors and metastases (Additional file 1: Table S2), an average of 67% of mutations were shared between primary tumors and matched distant metastases (Fig. 1) and the tumor mutation burden (TMB) was similar between primary NSCLC tumors and metastases (Additional file 2: Fig. S1A). To validate these findings, we analyzed published WES data from 35 pairs of primary NSCLCs and brain metastases from the Brastianos cohort [11]. Similarly, primary NSCLC tumors and paired metastases shared an average of 69% of mutations and harbored essentially the same TMB (Additional file 2: Fig. S1B). These data suggest that at the time the metastasis occurred, these tumors were already late in their course of molecular evolution when most somatic mutations have already occurred.

To precisely quantify the difference in mutational landscape in the context of intratumor heterogeneity (ITH), we calculated the genetic distance between primary NSCLC tumors and metastases based on the cancer cell fraction (CCF) of the mutations derived from PyClone considering variant allele frequency (VAF), tumor purity, and local copy number changes [19]. Compared to ITH data from the TRACERx NSCLC study [18], the genetic distance between primary tumors and paired metastases was greater than that between spatially separated tumor regions within the same tumors although the absolute difference was relatively small (Additional file 2: Fig. S2). This suggests that neoplastic progression of NSCLC may follow the clonal expansion model whereby spatially proximal cells are genetically more similar to each other [20].

### Monoclonal seeding is the predominant mode for metastasis in NSCLC

To investigate the mode of metastasis in this cohort of NSCLC, we computed CCF of all somatic single nucleotide variants using PyClone [19]. As shown in Fig. 1, all mutations shared by primary NSCLC tumors and paired metastatic tumors were clonal (defined as mutations clustered to the clone with the highest CCF) in both primary tumors and metastases. Next, we inferred the subclonal architecture of each tumor by correlating CCF of different clones based on the pigeonhole principle [21, 22] (Additional file 2: Fig. S3). The results demonstrate that only single founding clones were shared between primary NSCLC tumors and paired metastases and all subclones were private to primary tumors or paired metastases consistent with a monoclonal/monophyletic seeding mode during metastasis in this cohort of NSCLC patients. Analyses of the previously published 35 pairs of primary NSCLCs and metastases showed that 34 of the 35 patients (97%) followed the monoclonal seeding mode (Additional file 2: Fig. S4). Furthermore, we obtained WES data from 3 tumor regions of the brain metastasis of patient 8 in our cohort and 4 tumor regions of two brain metastases of patient PB0308 in the Brastianos cohort (Additional file 2: Fig. S5). Subclonal analysis of multiregional sequencing data was also consistent with the monoclonal seeding model except for one tumor region of PB0308 that harbored a small number of clones indicative of polyclonal seeding. Taken together, these data suggest monoclonal/monophyletic seeding may be a predominant mode of metastasis in the majority of NSCLCs.

### Mutational processes of shared and private mutations between primary NSCLCs and paired distant metastases

Each organ has its unique microenvironment, and tumor cells may be exposed to distinct environmental mutagens in different organs that could theoretically lead to distinct mutational processes. It is well known that different cancer types have distinct mutational signatures [23, 24]. However, whether the mutational processes change when NSCLC metastasizes to different organs has not been well studied. Shared mutations (between primary NSCLC tumors and metastases) and private mutations (specific to primary tumors or metastases) provide a unique opportunity to address this question. We next calculated the contribution of different mutational signatures in primary NSCLCs and paired metastases from the 7 patients in our cohort and 35 patients in the Brastianos cohort [11] to investigate whether the different mutational processes were operative in primary NSCLC tumors and metastases (Additional file 1: Table S3).

As shown in Additional file 2: Fig. S6, COSMIC signature 4 (associated with tobacco exposure) was the dominant mutational signature in both primary tumors and metastases reflecting the fact that a majority of patients (37/42; 88%) had a history of cigarette smoking. Other top mutational signatures in primary tumors included signature 1 (associated with spontaneous deamination), signature 2 (associated with APOBEC-mediated processes), signature 24 (exposure to aflatoxin), and signature 13 (associated with APOBEC-mediated processes), which were also the top mutational signatures in metastases.

To further dissect the mutational processes associated with early clonal expansion before metastasis took place versus later subclonal diversification in primary NSCLC tumors and metastases, we delineated the mutational signatures of shared mutations between primary NSCLC tumors and metastases representing early genomic events and mutations private to primary tumors or metastases representing later subclonal mutations in primary or metastases, respectively. As the mutations private to primary tumors or metastases were too few for signature analyses for most patients, we first did this analysis by combining shared or private mutations from all patients. As shown in Additional file 2: Fig. S7, signature 4 was the dominant mutational signature in shared clonal mutations, consistent with previous reports that mutations associated with tobacco carcinogen exposure were often early clonal mutations [18]. Signature 4 was also associated with a small proportion of mutations unique to metastases that might represent rare subclonal mutations in primary tumors missed by single sampling. On the other hand, signature 5 (etiology-unknown, associated with smoking in lung cancers [23]) and signature 30 (etiology-unknown, associated with base excision repair in model system [25]) emerged as the top mutational signatures for primary-only mutations and signatures 2 and 13, both of which are associated with APOBEC-mediated processes, had become the top mutational signatures for mutations identified exclusively in metastases. Additionally, signature 1 (associated with spontaneous deamination) and signature 3 (associated with homologous recombination, HR) also appeared among the top mutational processes in mutations unique to primary NSCLC tumors or metastases. Furthermore, we re-ran analyses at the individual patient level on the 14 patients with more than 50 mutations for shared, primary-only, and metastasis-only mutations (Additional file 1: Table S4). Similar to the results from combined mutations, the contribution of signature 4 was significantly higher in early mutations shared by primary NSCLC tumors and metastases: the average contribution was 58% in shared mutations, 12% in primary-only mutations, and 13% in metastasis-only mutations (*p* = 0.00037 for shared versus primary-only and *p* = 0.0017 for shared versus metastatic-only mutations). In addition, compared to shared mutations, primary-only mutations were significantly enriched for signatures 1, 3, 13, and 18, while metastasis-only mutations were significantly enriched for signatures 1 and 13 (Additional file 2: Fig. S8 and Fig. S9). Taken together, these results suggest distinct mutational processes might be operating at different molecular time during the neoplastic evolution of NSCLC. While smoking-associated processes may be the main driver for mutagenesis during early clonal expansion of primary NSCLC tumors, other mechanisms such as spontaneous deamination, HR-DNA repair, and APOBEC-mediated processes may have played more important roles during subclonal diversification in both primary tumors and metastases.

### Increased chromosome instability in metastases compared to paired primary tumors

**Fig. 2:**
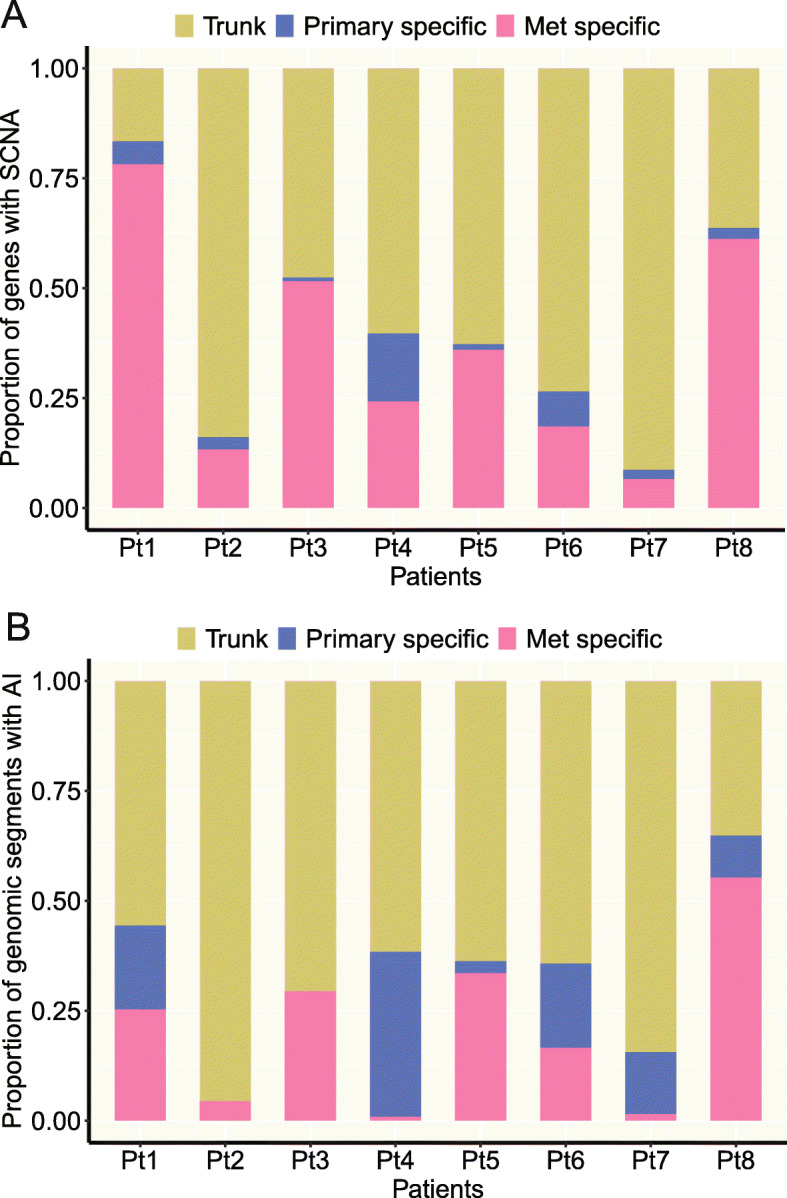
The level of concordance for somatic copy number aberrations (SCNAs) and allelic imbalance (AI). **a** The proportions of trunk, primary-specific, and metastasis-specific SCNA events. SCNA events were defined at gene level. Specifically, segment log2 ratio means were assigned to genes within each segment with SCNA so each sample would have log2 ratio values of the same number of genes for fair comparison between samples. **b** The proportions of trunk, primary-specific, and metastasis-specific AI events

Somatic copy number aberration (SCNA) is another key feature of human malignancies that could potentially impact expression of large groups of genes. We next delineated the genome-wide SCNA profiles of primary NSCLCs and paired distant metastases using a gene-based SCNA analysis algorithm for exome sequencing data that allows fair comparison of the SCNAs between different samples [26–28] to identify shared and unique SCNA events (Additional file 2: Fig. S10). To minimize the impact of tumor purity on SCNA analysis, we obtained purity-adjusted log2 copy number ratios for each tumor in this study (see the “Methods” section for details). As shown in Fig. 2a, on average, 59% of SCNA events were shared between primary tumors and paired metastases suggesting the majority of SCNA events were early molecular events during the carcinogenesis of these NSCLCs. Similarly, analysis of pairs of primary NSCLC tumors and brain metastases in the Brastianos cohort also revealed an average of 54% of SCNA events shared between primary NSCLCs and metastases from the same patients (Additional file 2: Fig. S11). Interestingly, patients with similar SCNA profiles between primary tumors and paired metastases did not necessarily have similar somatic mutation profiles (Spearman’s correlation of concordant ratio between SCNA profiles and somatic mutation profiles rho = 0.21, *p* = 0.19; Additional file 2: Fig. S12) suggesting SCNA and mutations were independent genomic events in these tumors. On the other hand, metastases demonstrated a significantly higher SCNA burden than paired primary tumors (*p* = 0.021) (Additional file 2: Fig. S13). No particular SCNAs were found to be enriched in metastases. Furthermore, compared to the ITH dataset from the TRACERx study [18], the SCNA landscape between primary tumors and paired metastases was more different than between spatially separated tumor regions within the same NSCLC tumors (*p* = 5.1e−11, Additional file 2: Fig. S14) once again supporting clonal expansion during metastasis of these NSCLC tumors.

Next, we compared genome-wide allelic imbalance (AI) events in primary tumors and matched metastases. We first used FACETS, a widely accepted algorithm for AI analysis [29], and observed a trend of higher AI burden in metastases although the difference did not reach statistical significance (Additional file 2: Fig. S15). We subsequently applied hapLOHseq, a sensitive algorithm specifically developed by our group to identify AI from exome sequencing data [30]. From hapLOHseq analysis, metastases demonstrated a higher AI burden than paired primary tumors by either the number of genomic segments subjected to AI (paired-sample Wilcoxon test *p* = 0.0079) or the total size of genome affected by AI (paired-sample Wilcoxon test *p* = 0.013) (Additional file 2: Fig. S16). Importantly, the majority of AI events (an average of 70% in our cohort and 82% in the Brastianos cohort) were shared between primary NSCLC tumors and matched metastases (Fig. 2b and Additional file 2: Fig. S17) suggesting that the majority of AI events had occurred prior to metastatic spread. We did not observe any particular AI events (chromosomal regions) that were enriched in primary tumors or metastases. Taken together, these results suggest that there might be a higher level of chromosomal instability (CIN) in metastases leading to more chromosomal aberrations. These findings are in line with TRACERx data that a higher level of CIN was associated with inferior survival [18].

### Majority of cancer gene mutations occur before metastasis

Next, we sought to investigate whether canonical cancer gene mutations were associated with metastasis. In our cohort, we identified 16 canonical cancer gene mutations defined as non-synonymous mutations of oncogenes and tumor suppressors leading to identical amino acid changes previously reported in cancers [31] or disrupting mutations in tumor suppressors (stop-gain, splicing, and frameshift INDELs) and 13 of 16 (81%) canonical cancer gene mutations were shared by primary and paired metastatic tumors, suggesting that these are acquired early prior to the occurrence of metastasis (Fig. 1). In patient 4, a frameshift deletion on beta2-microglobulin (*B2M*) was exclusively identified in the metastasis. In patient 8, two cancer gene mutations (a *MAP3K1* frameshift deletion and a *KDM6A* frameshift insertion) were identified only in the primary tumor, suggesting both were later molecular events in the primary tumor acquired after the spread of metastatic clones. Using the same definition, we identified 74 canonical cancer gene mutations from the 35 pairs of NSCLCs and brain metastases and 64 (86%) were shared between primary NSCLCs and brain metastases (Additional file 1: Table S5). Of note, 83% (148/178) of canonical cancer gene mutations were shared between regions of the same tumor in the TRACERx dataset [18] comparable to that between primary NSCLCs and paired metastases suggesting that a majority of canonical cancer gene mutations could have been acquired early before metastatic progression.

### Similar DNA methylation profiles between metastases and primary NSCLC tumors

**Fig. 3:**
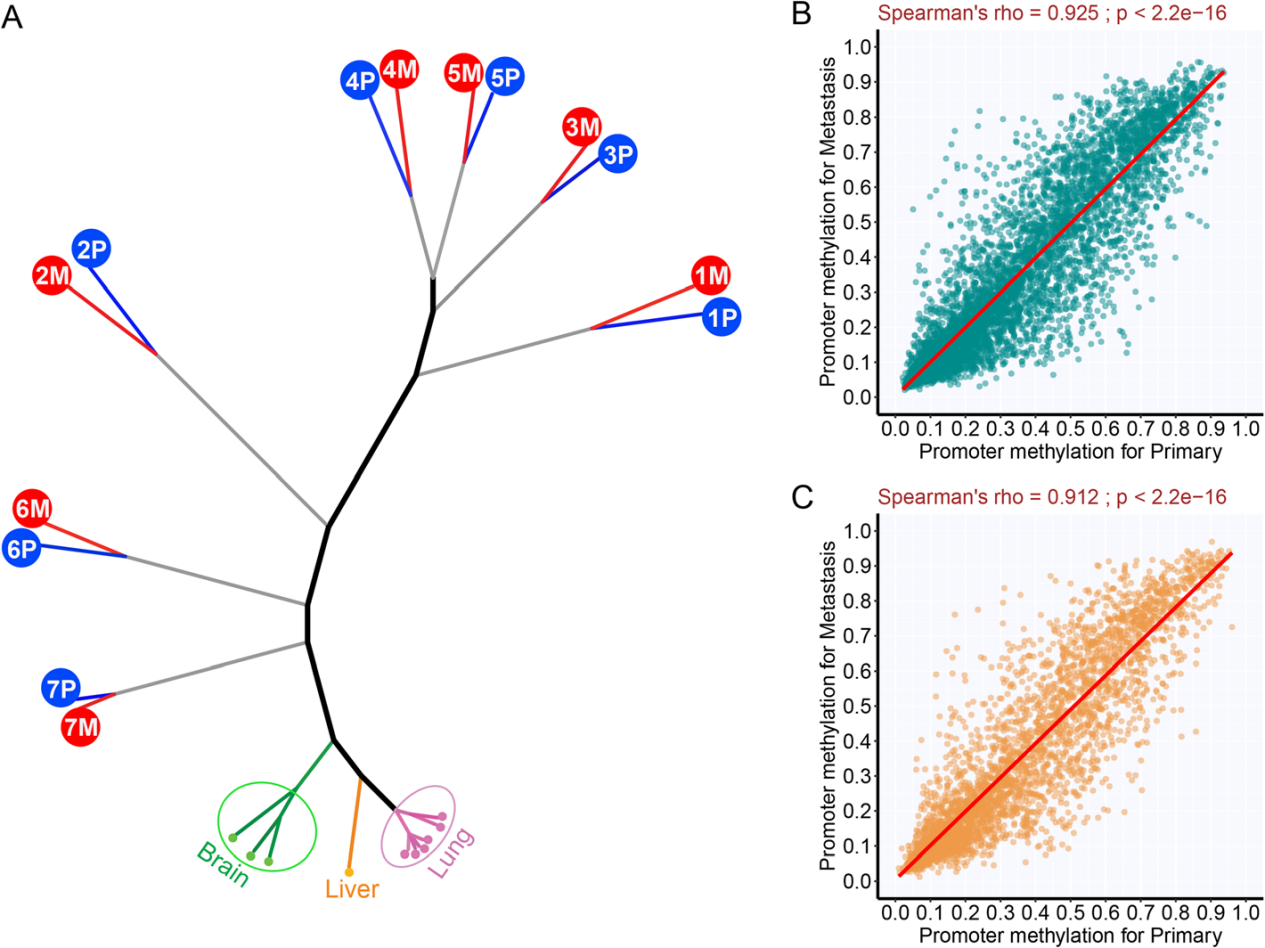
DNA methylation similarity between primary tumors and paired metastases. **a** Unsupervised clustering of all samples including normal tissues based on DNA methylation level of all CpG islands (*n* = 27,000). **b** Correlation of promoter DNA methylation between primary tumors and metastases for the 1084 genes showing a negative correlation between DNA methylation and gene expression (Spearman’s rank correlation ≤ − 0.5 based on the data in our main cohort). **c** Correlation of promoter DNA methylation level between primary tumors and metastases for the 521 genes previously reported to be regulated by DNA methylation in NSCLC from the study examining 73 cell lines

In addition to genomic aberrations, somatic epigenetic alterations, such as DNA methylation, may also impact neoplastic transformation and fitness. DNA methylation changes during metastatic progression have not been well documented in lung cancer. We next compared the methylome of primary tumors and paired metastases with the intent to identify DNA methylation changes associated with metastasis. Unsupervised hierarchical clustering using the methylation levels of approximately 27,000 CpG islands demonstrated that all tumor-adjacent uninvolved tissues from different patients clustered together based on the organ of origin (lung, brain, and liver) (Fig. 3a) separating remotely from tumor tissues highlighting the tissue-specific methylation patterns and significant divergence between uninvolved and tumor tissues. Furthermore, metastases clustered with their paired primary NSCLC tumors rather than with other metastases from other patients suggesting the marked heterogeneity in methylation patterns between individuals.

Next, we compared primary NSCLC tumors and metastases in a supervised approach to identify DNA methylation aberrations associated with metastasis. For this analysis, we focused on DNA methylation changes that could potentially impact expression of certain genes. We identified 1048 genes with promoter methylation levels negatively correlated with mRNA expression of the same tumors (Spearman’s rank correlation ≤ − 0.5 between promoter methylation and gene expression; Additional file 1: Table S6) based on the data from our main cohort of samples including normal samples (*n* = 24). The promoter methylation levels of these genes were well correlated between primary NSCLCs and metastatic tumors (*r* = 0.925, Fig. 3b), and none of the genes was significantly differentially methylated after adjusting for false discovery rate (FDR). Moreover, we scrutinized a set of genes that were previously reported to be significantly downregulated by DNA hypermethylation (*n* = 521) [32]. However, no single gene was differentially methylated between primary tumors and metastases from our main cohort (Fig. 3c and Additional file 1: Table S7). With the small sample size acknowledged, these consistent findings suggest the overall somatic methylation aberrations may have occurred before metastatic spread in this cohort of NSCLCs and were largely maintained during neoplastic evolution at both primary and metastatic sites.

### Upregulation of metabolic pathways and downregulation of immune pathways in metastases

**Fig. 4:**
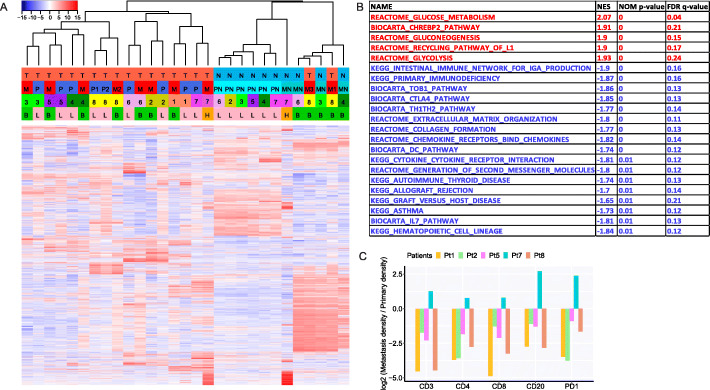
Differential signaling pathways in metastasis and immunohistochemical assessment of leukocyte antigens. **a** Unsupervised clustering of gene expression profiles using highly variable genes (standard deviation > 2.0; *n* = 4139). The complete linkage and 1-correlation distance metric were used. Each row represents a gene, and each column represents a sample. Tumor versus normal: T, tumor; N, normal. Tissue type: P, primary tumor; M, metastasis; PN, adjacent normal lung; MN, metastasis adjacent normal tissue. Organ: L, lung; B, brain; H, liver. **b** Upregulated and downregulated pathways in metastasis (nominal *p* < 0.01 and *q* < 0.25). Pathways in red are upregulated pathways in metastasis, and pathways in blue are downregulated pathways in metastasis. **c** Comparison of immune cell infiltration between primary NSCLC tumors and paired metastases by immunohistochemistry (IHC) of immune markers (CD3, CD4, CD8, CD20, and PD1). The density was defined as the number of cells positive for each marker per square millimeter. The *y* axis shows the ratio (log2) of density of each cell type in metastases versus that in paired primary lung cancers

To explore the potential molecular aberrations associated with metastasis beyond genomic and epigenetic changes, we compared the transcriptomic profiles between primary NSCLC tumors and metastases. Similar to DNA methylation aberrations, the unsupervised hierarchical clustering demonstrated that tumor-adjacent uninvolved lung tissues were separated from tumor samples overall and further clustered according to their organ of origin (Fig. 4a) highlighting the tissue-specific gene expression patterns as well as tumor-specific (irrespective of primary NSCLCs or metastases) transcriptomic changes. In the tumor cluster, metastases were overall more similar to their corresponding primary tumors than to each other, suggesting more pronounced inter- than intratumor gene expression heterogeneity.

Further, we compared the transcriptomic profiles of primary NSCLCs and metastases to identify specific pathways associated with metastases. Using gene set enrichment analysis (GSEA), 5 pathways were significantly upregulated and 17 pathways were significantly downregulated (*p* < 0.01 and FDR < 0.25) in metastases compared to primary NSCLC tumors (Fig. 4b). Upregulation of the recycling pathway of L1 known to be associated with neuronal development was noted, likely due to localization of 7 of 8 metastases within the brain. All remaining upregulated pathways were associated with metabolism. Interestingly, among the 17 signaling pathways downregulated in metastases, 14 were immune-related (Fig. 4b). Importantly, there was no significant association between TMB and tumor purity (cor = 0.19) suggesting that identification of up- or downregulated pathways was unlikely driven by tumor purity. Moreover, metastases showed significantly lower immune score [26] than primary tumors (Additional file 2: Fig. S18, *p* = 0.042). These patterns were accompanied by lower CD3, CD4, CD8, CD20, and PD-1 densities in metastases than primary tumors in 4 of 5 patients by IHC (Fig. 4c). Beyond the overall decreased infiltration of immune cells in metastases, a higher CD4/CD8 T cell ratio was seen, potentially supportive of the immunosuppressive environment predominating in metastases (Additional file 2: Fig. S19).

**Fig. 5:**
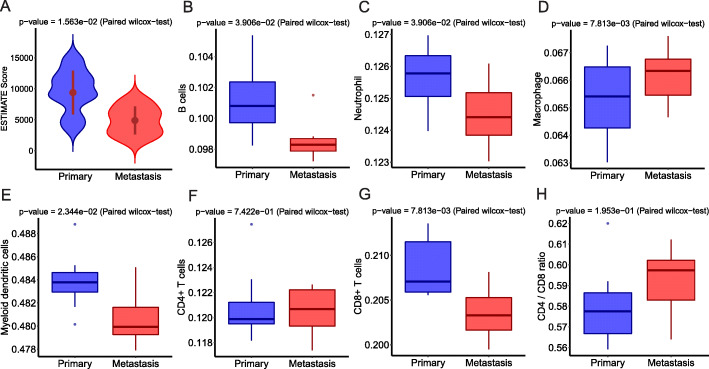
Immune cell infiltration in primary lung cancers versus metastases by deconvolution of transcriptomic profiles. **a** The overall immune cell infiltration was inferred by RNA-seq data using ESTIMATE. **b**–**g** The immune cell subsets were inferred by deconvolution of RNA-seq data using TIMER. The *y* axis represents the proportion of each immune cell type in the specimen. **h** The CD4/CD8 ratio inferred using TIMER. The difference was assessed by the paired-sample Wilcoxon test

To further understand downregulation of immune-related pathways in metastases, we deconvoluted the transcriptomic data using various algorithms. We first applied ESTIMATE, an algorithm estimating infiltration of overall immune cells [33], and the analysis revealed significantly lower ESTIMATE scores in metastases than primary tumors (Fig. 5a) suggesting an overall lower immune cell infiltration in metastases. Next, we deconvoluted gene expression data by TIMER [34] to infer the infiltration of main immune cell types. As shown in Fig. 5b–g, the infiltration of most immune cell types was higher in primary lung cancers than metastases with the exception of macrophages, which were higher in metastases (Fig. 5d) and CD4+ T cells that were similar between primary and metastases (Fig. 5f), which led to higher CD4/CD8 ratio in metastases, although the difference did not reach statistical significance (Fig. 5h). We further applied CIBERSORT to classify the immune subsets at a more granular level [35]. Unfortunately, the inferred infiltration of most immune cell subsets derived by CIBERSORT was very low in this cohort of tumors (Additional file 2: Fig. S20) precluding comparison between primary tumors and metastases. Nevertheless, CIBERSORT data suggested the macrophages were predominately pro-tumor M2 macrophages [36], which were significantly higher in metastases (Additional file 2: Fig. S20H). Taken together, these results suggest that downregulation of immune-related pathways was mainly due to reduced immune cell infiltration in metastases.

### Immunosuppression may not be due to immune privilege

As 7 of 8 metastatic tumors were in the brain, immune privilege of the central nervous system (CNS) [37–39] and/or steroid treatment prior to resection could have contributed to downregulation of immune pathways. To evaluate whether the immunosuppression only applied to brain metastases, we first specifically analyzed patient 7, who had a liver metastasis. Importantly, not only did liver metastasis show a lower immune score than the primary tumor (Additional file 2: Fig. S18B), but the GSEA analysis using patient 7 only also showed that 15/17 downregulated signaling pathways in the liver metastasis were directly related to immune responses (Additional file 1: Table S8). Next, we analyzed a recently published treatment-naïve NSCLC patient dataset with extracranial metastases [16] and found that in 3 of 4 patients, metastatic samples showed a lower immune score than their primary counterparts (Additional file 2: Fig. S21). Furthermore, we re-analyzed transcriptomic data from 4 extracranial metastases from a genetically engineered mouse model (GEMM) of human lung adenocarcinoma [17]. Once again, immune scores were decreased specifically in metastases (Additional file 2: Fig. S21). Taken together, these data suggest that downregulation of immune pathways may be a common phenomenon associated with NSCLC metastases irrespective of site.

### Genomic basis for immunosuppression in metastases

As mentioned above, a higher SCNA burden in metastases has been associated with immunosuppression in multiple cancer types [40]. We attempted to identify additional genomic aberrations that could contribute to the suppressive immune microenvironment in metastases but did not detect mutations of any gene critical to immune surveillance exclusively in metastases [41] from the combined WES cohort of 43 patients. We next evaluated loss of heterozygosity (LOH) of human leukocyte antigen (HLA) class I, a potential immune evasion mechanism in cancer [42, 43], and found 16 of 43 patients harbored HLA LOH in either primary NSCLC and/or metastases (Additional file 2: Fig. S22). Among these 16 patients, 5 harbored HLA LOH only in metastases, while 1 patient had HLA LOH only in the primary tumor. Interestingly, HLA LOH was associated with a higher SCNA burden, chromosomal losses in particular, regardless of primary or metastasis (Additional file 2: Fig. S23). We further compared the expression of HLA genes between the 8 pairs of primary NSCLCs and matched distant metastases to explore whether decreased HLA expression may lead to ineffective neoantigen presentation and immune evasion. As shown in Additional file 2: Fig. S24, the expression of HLA genes was overall lower in metastases, but the difference did not reach statistical significance.

## Discussion

Lung cancer is the leading cause of cancer mortality globally and metastases account for most deaths. Little is known about the timing and mechanisms underlying metastasis. Metastasis is believed to be an evolutionary process shaped by the dynamic interaction of tumor cells with host factors, particularly host anti-tumor immune surveillance. The molecular aberrations of tumors as well as tumor microenvironment at different molecular levels could profoundly impact this process, and comprehensive molecular analyses of primary tumors and distant metastases are warranted to depict cancer metastatic evolution. Through multiomics analyses of primary NSCLC tumors and paired distant metastases, we demonstrated that overall, metastases resemble their corresponding primary NSCLC tumors closely regarding shared somatic mutations including canonical cancer gene mutations, DNA methylation profiles, and transcriptomic profiles suggesting a majority of these are early molecular events occurring before metastatic spread. On the other hand, metastases differed from primary tumors by showing more profound immunosuppressive microenvironment than primary tumors. It suggests that cells with metastatic plasticity may be the ones enabled to escape immunosurveillance, thus leading to successful metastasis to distant organs.

Monoclonal and polyclonal seeding represent the two main hypothesized modes of metastasis. In monoclonal seeding, clones in the primary tumor compete and a single clone or cell eventually seeds the metastases, while in the polyclonal seeding hypothesis, multiple distinct clones cooperatively, collectively, or independently seed the metastases [21, 22, 44]. Through comparison of the subclonal architecture of primary NSCLC tumors and their paired distant metastases, we discovered evidence of monoclonal seeding in 41 of 42 tumors (one patient without germ line control was excluded). Similarly, in their pioneer study comparing brain metastasis to matched primary tumors, Brastianos et al. revealed monoclonal seeding in a majority of cancer types including renal cell carcinoma, ovarian cancer, breast cancer, melanoma, and esophageal cancer [11] suggesting that monoclonal seeding is a predominant mode of metastasis regardless of cancer type or metastatic site.

Another critical question is what capacities are associated with cells that are capable of metastasis. *Xenograft* studies have suggested that metastases originate from particular subclones with distinct “metastatic” profiles [45–48] while others have shown that metastases develop through stochastic events from primary tumor cells with an equal metastatic potential [49, 50]. In our study, comparison of transcriptomic profiles of metastases to primary NSCLCs revealed a more suppressed immune microenvironment in metastases. This was supported by the lower densities of immune cell infiltration in metastases, which could highlight immune exclusion or a lack of ability to proliferate locally within the tumor. This is also supported by the lack of PD-1 expression in metastases, which could highlight the inability of T cells to become activated and expand upon encountering their cognate antigens within tumors. Furthermore, though the overall immune cell densities were lower in metastases, we evaluated the relationship between cell types considered to be pro- and anti-tumor to gain insight into their balance within the tumor microenvironment. These analyses demonstrated a higher level of infiltration of M2 macrophages known to associate with immunosuppressive tumor microenvironment [51] in metastases. These are complemented by the higher CD4/CD8 ratio in metastases, which could portend the presence of regulatory T cells further known to inhibit anti-tumor responses, though our inability to evaluate additional markers prevents us from confirming this hypothesis.

Immunosuppression in brain metastases has been reported [15, 52, 53]. However, these findings have been confounded by immune privilege associated with the blood-brain barrier (BBB) [37–39]. Here, suppressed immune pathways were observed not only in brain metastases, but also in extracranial metastases in both human autopsy cases and murine GEMM lung cancers suggesting immune evasion in metastases may be a universal phenomenon in NSCLC. Metastasis involves cancer cells breaking away from the primary tumor, traveling through blood or lymphatics, and forming a new tumor at other sites. During this process, cancer cells must survive many challenges, particularly immune surveillance. Therefore, being able to escape from destruction by immune cells is necessary for cancer cells to successfully metastasize, which has been experimentally demonstrated in animal studies [54, 55]. Cancer cell shedding into blood is not uncommon, even for early stage diseases [56], though not all patients with circulating tumor cells develop metastases. Therefore, it is possible that only the cells capable of escaping immune surveillance could survive and form distant metastases.

In our search for unique “metastatic” genomic profiles, in addition to a previous report of more HLA loss in metastasis [42], distant metastases showed more SCNA and genome-wide AI events than their primary counterparts. Emerging evidence has shown that SCNA is associated with a cold immune microenvironment across cancer types [26, 57]. The exact mechanisms underlying the association between SCNA and immunosuppression are not well understood. Several hypotheses including relatively low neoantigen concentration and protein imbalance leading to impaired tumor signal in high aneuploidy tumors were proposed [40]. It is possible that, at least in a subset of NSCLCs, different cancer cells with different SCNA/AI profiles break off from tumor cells and only the cells with high SCNA/AI burdens can survive the immune attack during seeding and become metastatic clones. Furthermore, the HLA gene expression also appeared to be lower in metastases, although the difference did not reach statistical significance, which could be due to small sample size. Given the heterogeneous nature of NSCLC, the molecular mechanisms leading to immune evasion are likely heterogeneous across different tumors. Further studies with larger cohorts of primary metastasis are needed to identify particular molecular features associated with immunosuppression in metastases.

One important limitation of the current study is the small sample size, particularly for transcriptomic profiling analyses. Unfortunately, there are very little molecular profiling data available from lung cancer distant metastases even for unpaired specimens. For example, MET500 is a comprehensive molecular profiling study on 500 adult patients with metastatic solid tumors, thus far the largest dataset on molecular profiling of metastases [58]. However, there were only 24 lung cancer patients in the cohort and 5 specimens of distant metastases (*n* = 3 for liver; *n* = 1 for adrenal; *n* = 1 for brain). This highlights the need for more multiomics studies on lung cancer metastases, to underscore the molecular mechanisms underlying distant metastases of NSCLC.

Immunosuppression has already occurred in primary lung cancers [14, 15]. Our current data revealed more profound immunosuppression in metastases than primary tumors, in line with reports that immune checkpoint blockade therapy may have different efficacy in primary NSCLC tumors versus metastases [59]. Future comprehensive profiling studies with larger cohorts of ideally paired primary NSCLCs and metastases have the potential to identify distinct immunosuppressive features in primary tumors and metastases to provide novel insights into precise immunotherapy strategies for patients exhibiting different primary versus metastatic tumor burdens.

## Conclusions

Metastasis may be a late event during the molecular evolution of NSCLC when the majority of genomic and epigenetic events have occurred. Immunosuppression, endowed by different molecular features such as somatic copy number aberrations, may be a common characteristic of cancer cells with metastatic plasticity in NSCLC.

## Methods

### Patient cohort and sample collection

Peripheral blood, primary NSCLC tumors, distant metastases, and tumor-adjacent normal tissues were collected from 7 patients with metachronous metastatic NSCLC and one patient with synchronous metastatic NSCLC (Additional file 1: Table S2). Peripheral blood mononuclear cells (PBMC) were immediately isolated from 10 ml whole blood and stored at − 80 °C. Surgical specimens were snap frozen in liquid nitrogen immediately after surgical resection and stored at − 80 °C. All surgical specimens were subjected to pathological examination to confirm the diagnosis and ensure the sample quality before DNA or RNA extraction.

### Whole exome sequencing

Genomic DNA was extracted and subjected to library preparation for sequencing with Agilent SureSelect Human All Exon V4 kit according to the manufacturer’s instructions. The 76-bppaired-end WES was performed on Illumina HiSeq 2000 platform with mean target coverages of 200× and 100× for tumor and normal samples, respectively.

### Somatic mutation calling

We ran MuTect [60] for somatic single nucleotide variants (SNVs), and Pindel [61] for somatic small insertions and deletions (INDELs). Mutations previously reported in public database (dbSNP138, 1000Genomes, ESP6500, and EXAC) with > 1% allele frequency were removed. Next, we applied the following mutation filtering criteria: (i) sequencing depth ≥ 50 for tumor and ≥ 30 for normal, (ii) tumor allele frequency ≥ 5% for single nucleotide variants and ≥ 10% for INDELs, and (iii) normal allele frequency < 1%.

### Genetic distance calculation

Using cancer cell fractions (CCFs) of all somatic mutations including SNVs and INDELs, we calculated genetic distance between any given two samples (primary tumor versus paired metastasis or two different regions of the same tumors). We used three different definitions of genetic distance. First, Nei’s genetic distance [62, 63] was calculated as follows:

$$ D=-\ln \frac{\sum \left({x}_i{y}_i+\left(1-{x}_i\right)\left(1-{y}_i\right)\right)}{\sqrt{\left(\sum {x_i}^2+{\left(1-{x}_i\right)}^2\right)\left(\sum {y_i}^2+{\left(1-{y}_i\right)}^2\right)}} $$

Here, *x*_*i*_ and *y*_*i*_ denote the CCFs of the *i*th mutation in sample *x* and sample *y*, respectively.

Next, we calculated the genetic distance by taking the mean of absolute CCF difference defined as below:

$$ D=\frac{\sum \left|{x}_i-{y}_i\right|}{n} $$

In the formula, *x*_*i*_ and *y*_*i*_ denote the CCFs of the *i*th mutation in sample *x* and sample *y*, respectively, and *n* is the number of total mutations.

Finally, the Jaccard distance [64] was calculated as follows:

$$ D=\frac{\sum {\left({x}_i-{y}_i\right)}^2}{\sum {x_i}^2+\sum {y_i}^2-\sum \left({x}_i{y}_i\right)} $$

In the Jaccard distance definition, *x*_*i*_ and *y*_*i*_ denote the CCFs of the *i*th mutation in sample *x* and sample *y*, respectively.

For the ITH dataset, one tumor can have multiple two-sample combinations. In this case, we took the average distance among all the possible pairwise combinations.

### Mutational signature analysis

Mutation signatures were determined by deconstructSigs [65] with 30 COSMIC signatures provided by the package.

### Somatic copy number aberration analysis

We applied ExomeCN, a gene-based SCNA analysis algorithm for exome sequencing data that allows fair comparison of the SCNAs between different samples. Specifically, we first obtained copy number segments with copy ratios between tumor and normal. The log2 copy number ratios of the segments were then assigned to the genes within the segments by CNTools [66], so each sample would have log2 ratio values of the same number of genes for fair comparison between samples. We defined copy number gains and losses in all tumor samples using +log_2_1.5 for gain and −log_2_1.5 for loss, respectively. Since the signal to noise ratio of SCNA could be reduced in the samples with lower tumor purity, we obtained purity-adjusted log_2_ ratios by log_2_((original copy ratio − 1)/purity + 1) [67] if any of the paired samples from the same patients passed the original log_2_ thresholds of +log_2_1.5 and −log_2_1.5. Tumor purity was estimated by Sequenza [68]. Copy number gain burden and loss burden were defined as the number of copy number gains and losses in a given sample.

### Allelic imbalance analysis

We applied two algorithms for the AI analysis, FACETS [29] and hapLOHseq [30]. FACETS is an allele-specific copy number analysis pipeline utilizing next-generation sequencing (NGS) data. It processes BAM file and segments total- and allele-specific read counts to estimate integer copy number calls adjusted for tumor purity, ploidy, and clonal heterogeneity. For hapLOHseq, the germline variants were first called with GATK and germline haplotypes were then estimated using the simple phaser utility of hapLOHseq. Next, we ran hapLOHseq to identify putative regions of AI in all samples. For each patient, we took the hapLOHseq AI event calls that were made for each sample, one at a time, and tested if these events existed in the other samples of the same individual using a binomial test, assessing if there is higher than expected phase concordance of 0.5 over a tested region (*p* < 1e−4) [69]. Finally, for each patient, we characterized segments of the genome as follows: normal (no AI in any sample), norm-specific (AI region unique to the normal tissue), private (AI region only seen in a single tumor sample of a patient), shared (AI region seen in multiple samples of a patient), met-specific (AI region unique to a metastatic sample), or trunk (AI region seen in all samples of a patient’s tumor, or in the case of metastatic patients, AI regions seen in both the primary and metastatic samples of a patient).

### Subclonal architecture analysis and phylogeny inference

For each patient, we ran PyClone [19] with 10,000 iterations and 1000 burn-in parameters. To be specific, we only used somatic SNVs because their variant allele frequency is expected to be relatively more accurate than INDELs. With local copy number data obtained from Sequenza [68], the PyClone was run with paired samples (primary and metastasis). We only considered mutation clusters with at least 5 mutations. To infer phylogenetic trees, mutation data was converted to the binary data with mutations being 1 and wild-type being 0 and fed into Phangorn R package. Tree topologies were estimated by *pratchet*, and branch lengths were inferred by *acctran*.

### DNA methylation analysis

Methylation profiling was done using Infinium MethylationEPIC Kit (Illumina, Inc.) according to manufacturer’s protocol. Data normalization and beta value determination were carried out using Illumina GenomeStudio Software with the additional Methylation Module Software. RnBeads package in R was then used to derive 27,000 CpG island methylation values. The hierarchical clustering based on DNA methylation level of the CpG islands was performed with the correlation distance metric (1 – Pearson’s correlation coefficient). We identified a list of genes whose promoter DNA methylation was negatively correlated with mRNA expression (Spearman’s rank correlation coefficient ≤ − 0.5) by correlating DNA methylation and gene expression data of all samples including normal tissues in our cohort. We also obtained previously reported 521 Significantly Repressed in Association with DNA Methylation (SRAM) genes known to be regulated by DNA methylation in NSCLC from the study examining 73 cell lines [32] and compared their promoter DNA methylation levels between primary tumors and paired metastases in our cohort.

### RNA sequencing

Extracted RNA was converted to cDNA using Ribo-SPIA Technology (NuGEN, San Carlos, CA). The cDNA library was then sequenced on Illumina HiSeq 2000 platform using 76 bp paired-end reads.

### Transcriptomic data processing and gene set enrichment analysis

RNA sequencing reads were mapped to the hg19 reference genome using the STAR aligner [70]. For calculation of gene expression, raw count data of each gene were first obtained using HTSeq [71] and normalized by scaling the raw library size using calcNormFactors in edgeR package in R [72]. Then, Voom [73] transformation was applied to normalized counts and a linear model fit to the data for differential expression analysis using Limma package [74]. To identify upregulated and downregulated pathways in metastasis, we run GSEA (v3.0) [75, 76] against the combined gene sets including KEGG, BioCarta, and Reactome.

### Estimation of immune infiltration by de-convolution of transcriptomic data

Immune scores were calculated by taking the average of normalized expression levels of 40 genes including cytolytic markers, HLA molecules, genes associated with IFN-γ pathway, chemokines, and adhesion molecules as previously described [26]. The overall immune cell infiltration was derived using ESTIMATE [33] and various immune cell subtypes were inferred by TIMER [34].

### Immunohistochemistry

Immune profiling by IHC with multiple immune markers including CD3, CD4, CD8, CD20, and PD1 was performed on primary tumors and matched distant metastases as previously described [77]. The density was defined as the number of cells positive for each marker per square millimeter.

## Supplementary information

**Additional file 1:** Supplementary Tables S1-S8.

**Additional file 2:** Supplementary Figs. S1-S22.

**Additional file 3:** Review history.

## Data Availability

Whole exome sequencing, RNA-seq, and DNA methylation microarray data were deposited in European Genome-phenome Archive (EGA) with the accession codes EGAS00001004077 [78], EGAS00001004078 [79], and EGAS00001004079 [80], respectively. The previously published WES data from 35 pairs of primary NSCLC tumors and matched brain metastases (Brastianos cohort) [11] was obtained from the database of Genotypes and Phenotypes (dbGaP) with the accession number phs000730.v1.p1 [81]. Multiregion WES data of 100 early-stage NSCLC tumors enrolled in TRACERx [18] was utilized to infer the genetic distance between spatially separated regions within the same tumors. The RNA-seq data from primary NSCLC tumors and multiple metastases at the time of autopsy from 4 treatment-naïve NSCLC patients was obtained from a previous publication [16]. The microarray gene expression data generated from the metastatic lung cancer mouse model [17] was obtained from Gene Expression Omnibus (GEO) data repository (GSE14449) [82].
