# Supplementary material for: Multiomics profiling of primary lung cancers and distant metastases reveals immunosuppression as a common characteristic of tumor cells with metastatic plasticity
Source: Genome Biol. 2020 Nov 4;21:271. doi: 10.1186/s13059-020-02175-0 (PMC7640699; doi:10.1186/s13059-020-02175-0)
Supplement: Supplementary file 2 — Additional file 2: Supplementary Figs. S1-S22. [file 13059_2020_2175_MOESM2_ESM.docx]

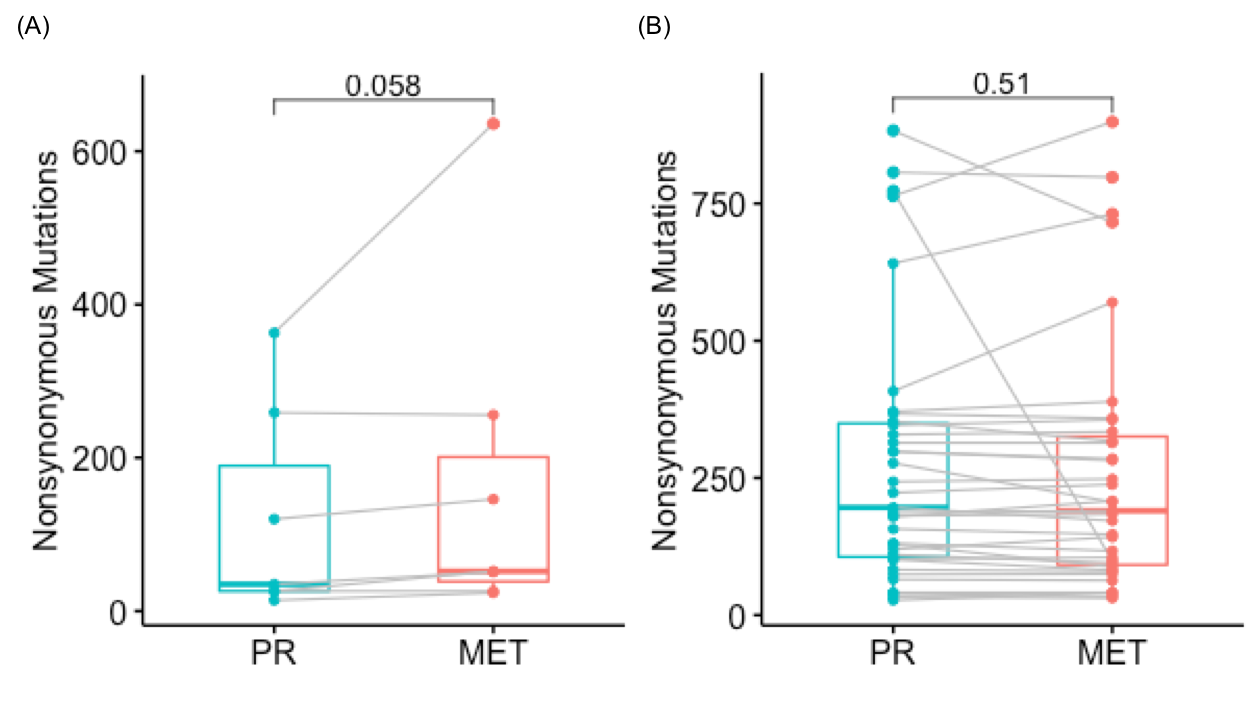


**Fig. S1. Comparison of tumor mutational burden (TMB)**

**(A)** TMB comparison between 7 pairs of primary tumors and metastases from our main cohort. **(B)** TMB comparison between 35 pairs of primary tumors and metastases from the external cohort (*Brastianos* *et el.*, *Cancer Discovery*, *2015*).

**C**

**A**

**B**

**Fig. S2. Genetic distance of somatic mutations**

The pairwise genetic distance was compared between spatially separated tumor regions of the same tumors using intra-tumor heterogeneity (ITH) dataset (*Jamal-Hanjani M, et al., NEJM, 2017*) versus the 42 pairs of primary tumors and distant metastases (PM) in the current study. The genetic distance was calculated based on the cancer cell fraction (CCF) of all mutations inferred by PyClone adjusted for purity and local copy number changes. (**A**) Nei’s genetic distance. (**B**) The mean absolute cancer cell fraction (CCF) difference. (**C**) Jaccard distance. P-value was calculated by Wilcoxon rank-sum test.


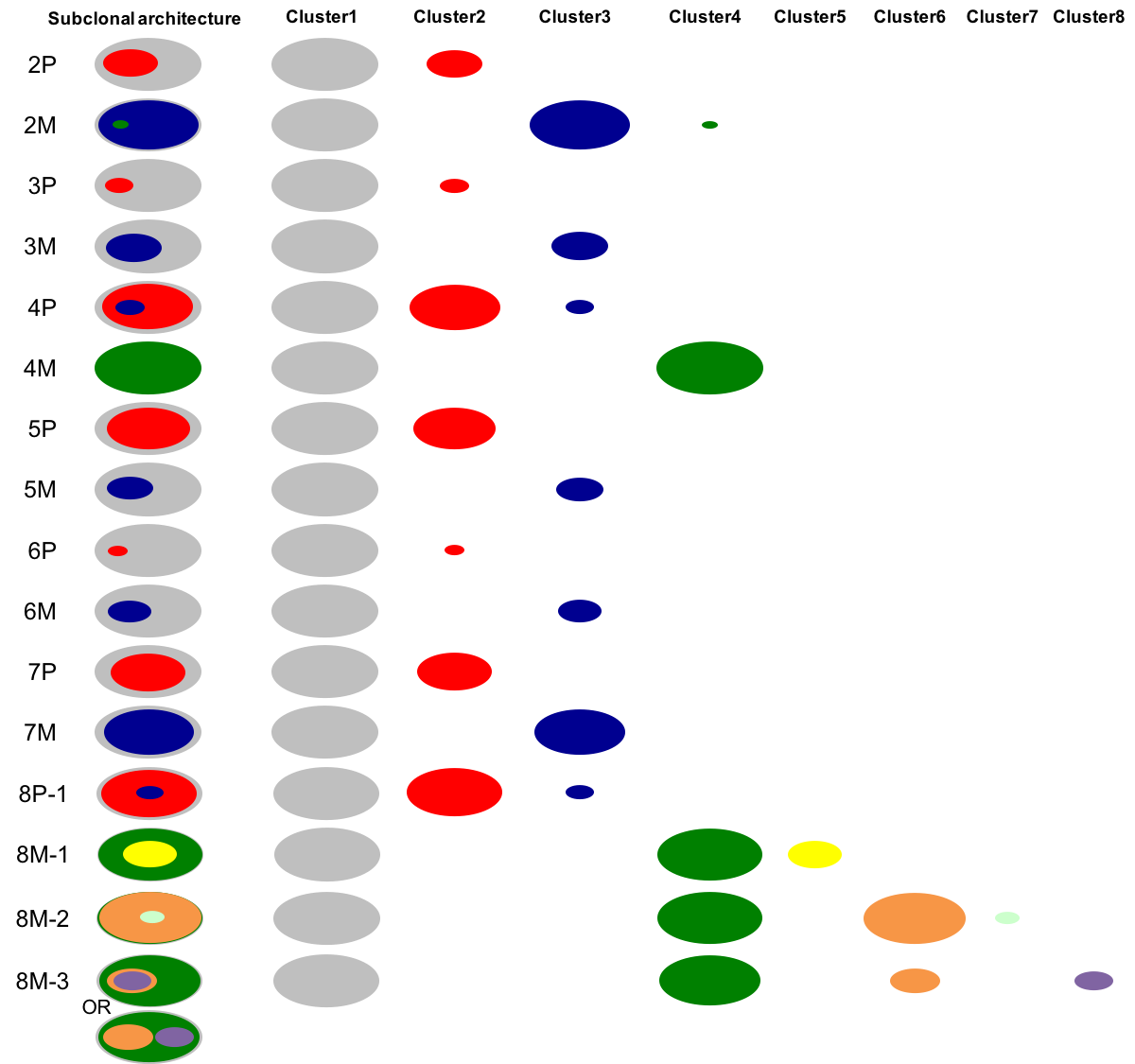


**Fig. S3. Subclonal architecture for all tumor samples in our main cohort**

The size of ovals is proportional to the cancer cell fraction (CCF) of each mutation cluster. The column on the far left shows the final subclonal architecture constructed from mutation clusters based on pigeonhole principle. Cluster 1 represents a founding clone with the largest CCF, while other clusters are considered as subclones.


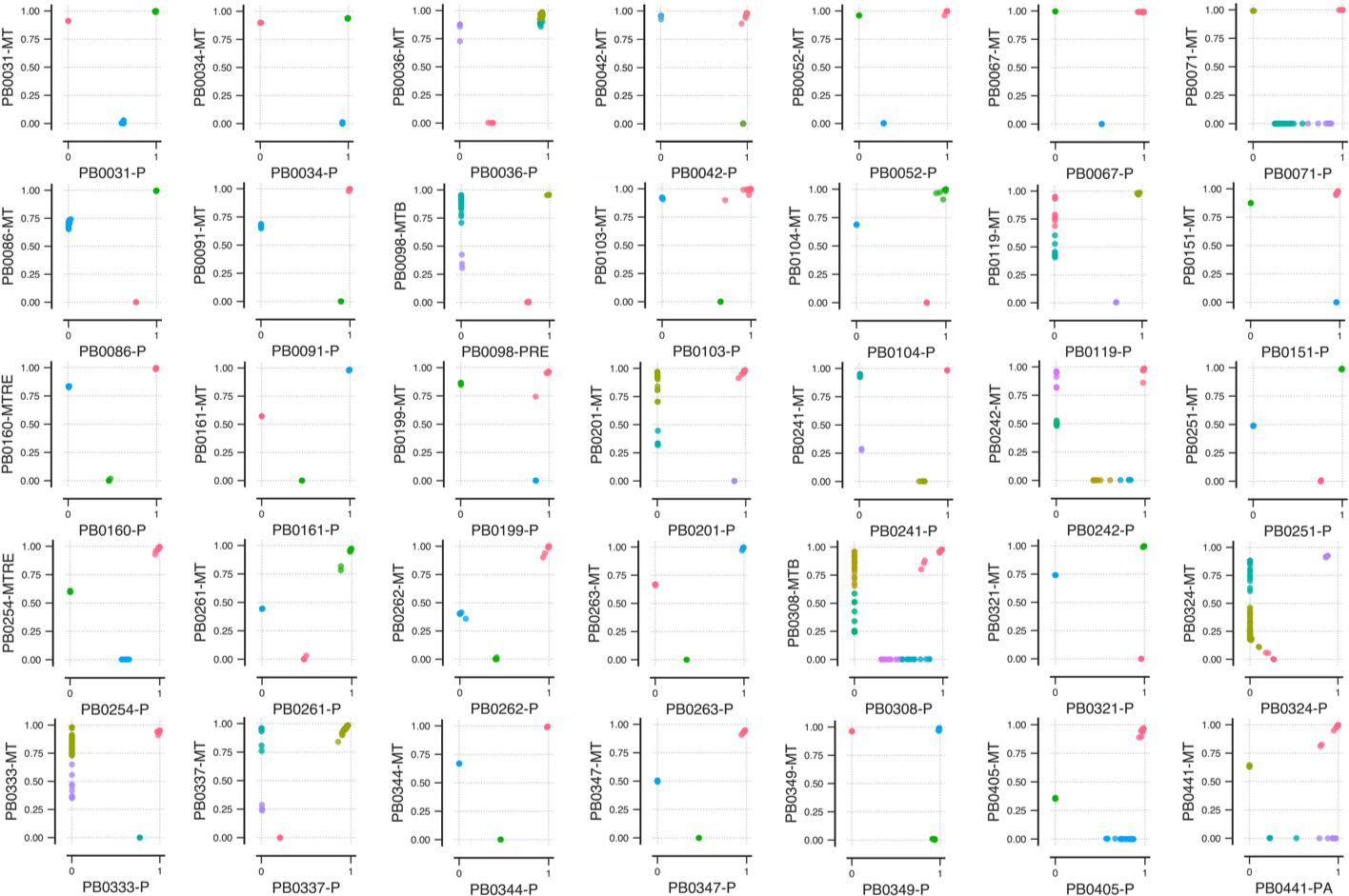


**Fig. S4. Pairwise cancer cell fraction plots between primary tumors and paired metastases**

Pairwise cancer cell fraction plots for 35 pairs of primary tumors and brain metastases (*Brastianos* *et el.*, *Cancer Discovery*, *2015*). The dots with different colors represent the mutations belonging to different clones estimated by PyClone. Clusters with less than 5 mutations are not shown.


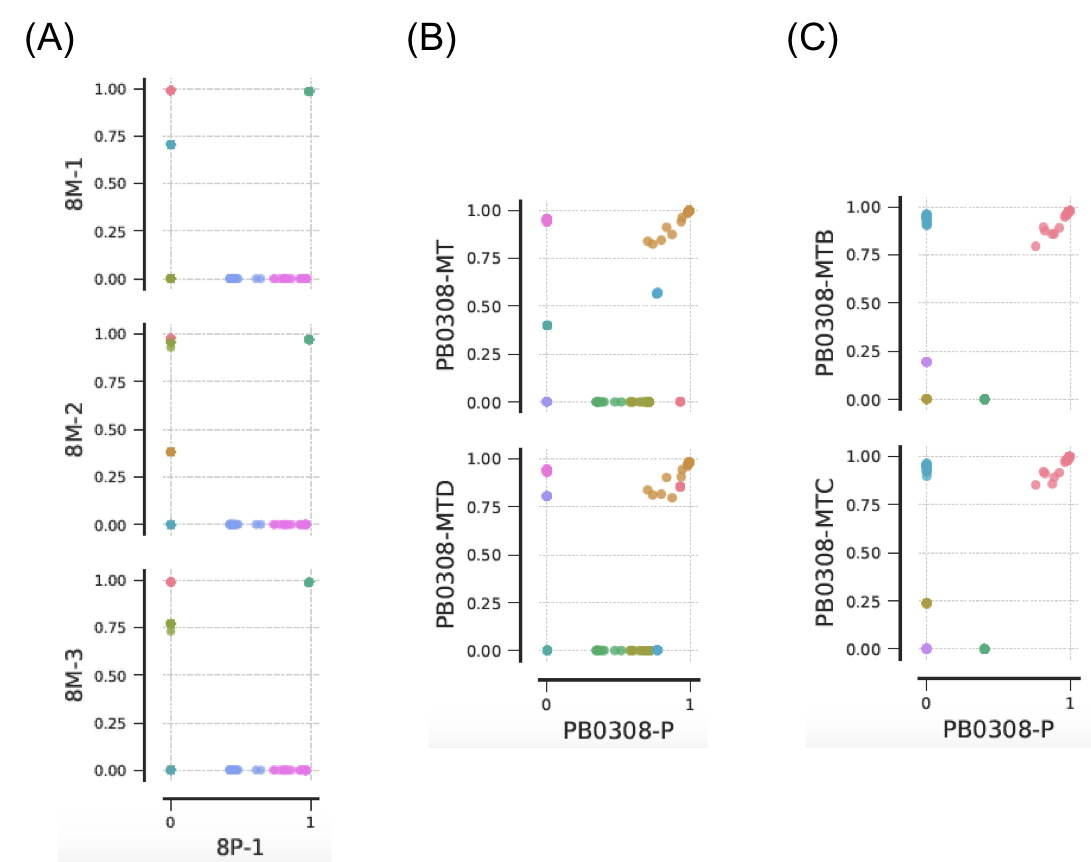


**Fig. S5. Pairwise cancer cell fraction plots between primary tumors and paired metastases with multi-region sequencing**


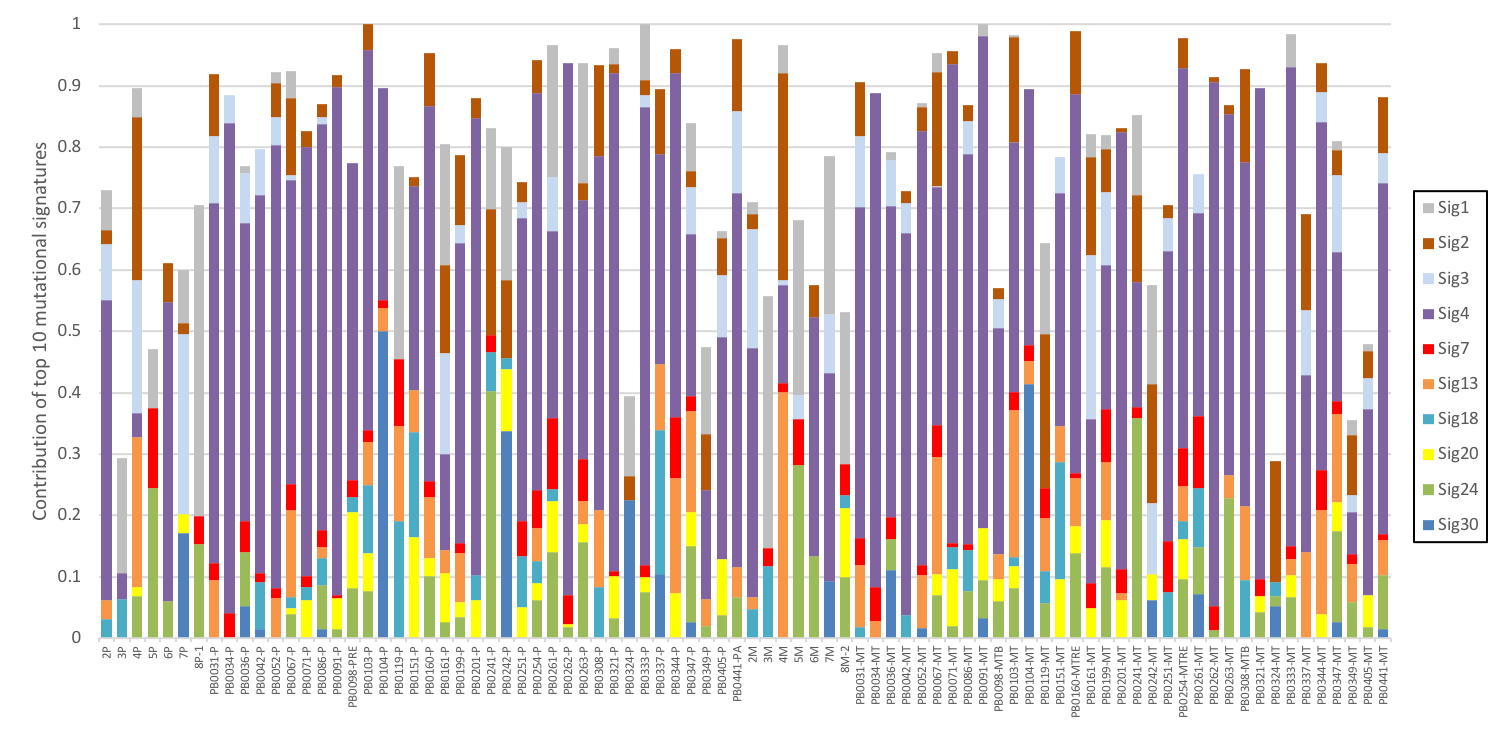


**Fig. S6. Contribution of the top 10 mutational signatures for individual tumors**

Relative contributions of the top 10 mutational signatures across all samples (42 primary tumors and 42 metastases).


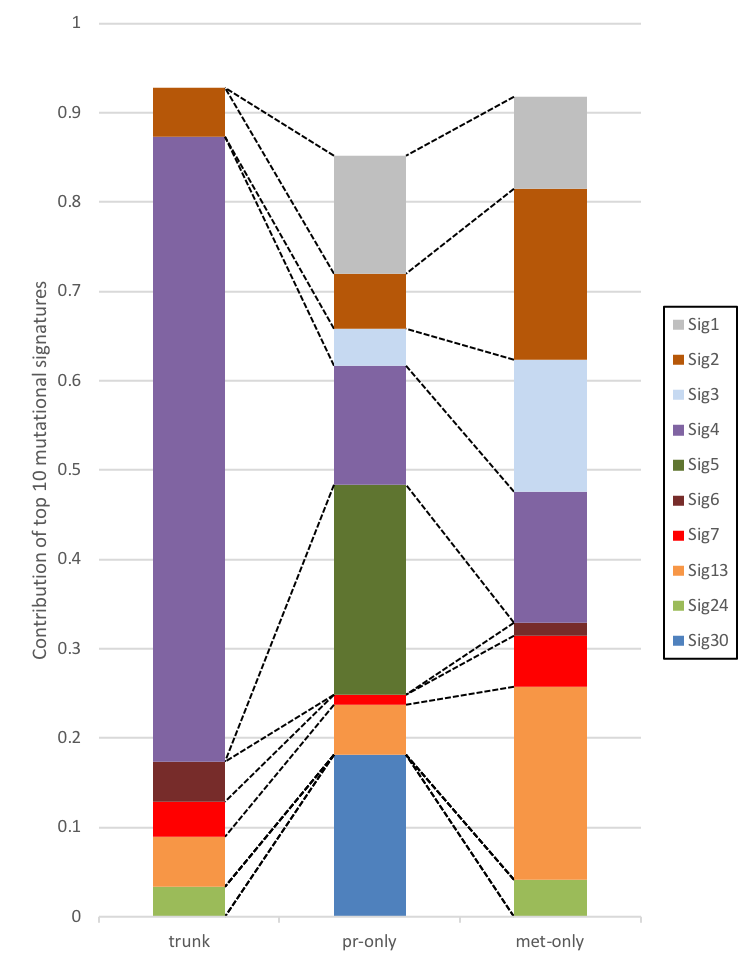


**Fig. S7. Contribution of the top 10 mutational signatures for trunk, primary-only, and metastasis-only mutations (combined)**

Relative contributions of the top 10 mutational signatures across mutations shared by primary tumors and metastases (trunk), only detected in primary tumors (pr-only), or only detected in metastases (met-only). For each category, mutations from different patients were combined.


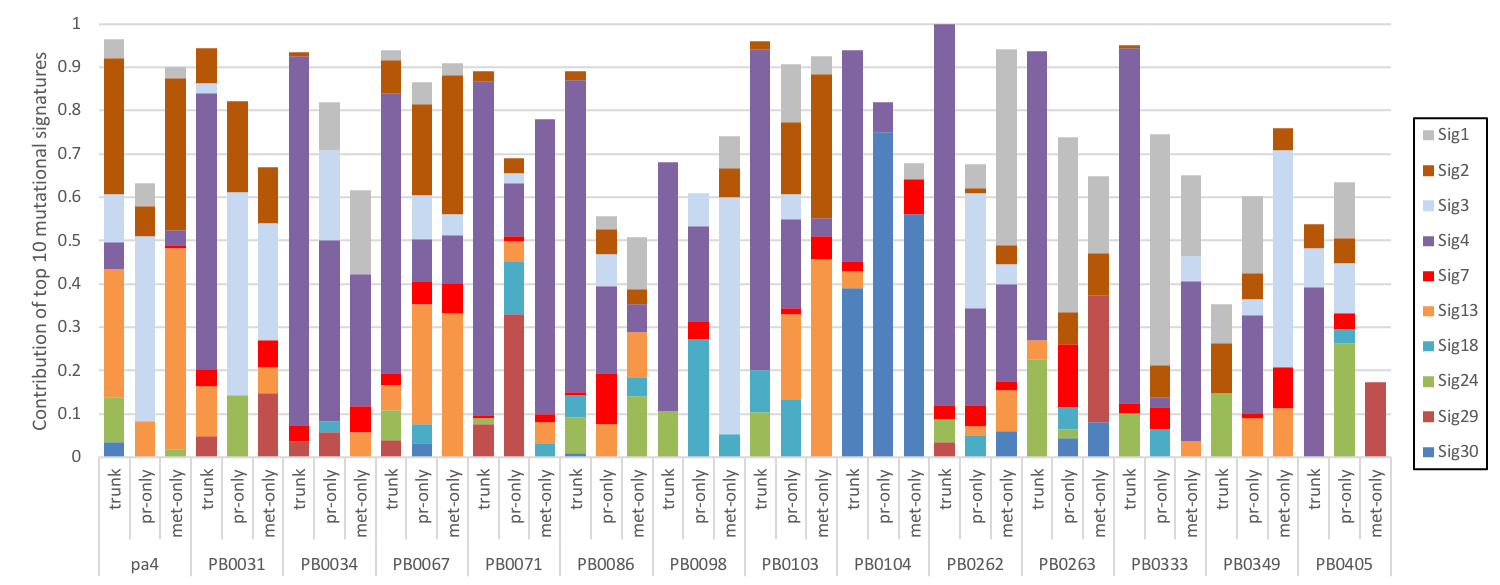


**Fig. S8. Contribution of the top 10 mutational signatures for trunk, primary-only, and metastasis-only mutations by individual patient**

Relative contributions of the top 10 mutational signatures across mutations shared by primary tumors and metastases (trunk), only detected in primary tumors (pr-only), or only detected in metastases (met-only) by individual patient. Only the 14 patients with more than 50 mutations for trunk, primary-only and metastasis-only mutations were included in this analysis.


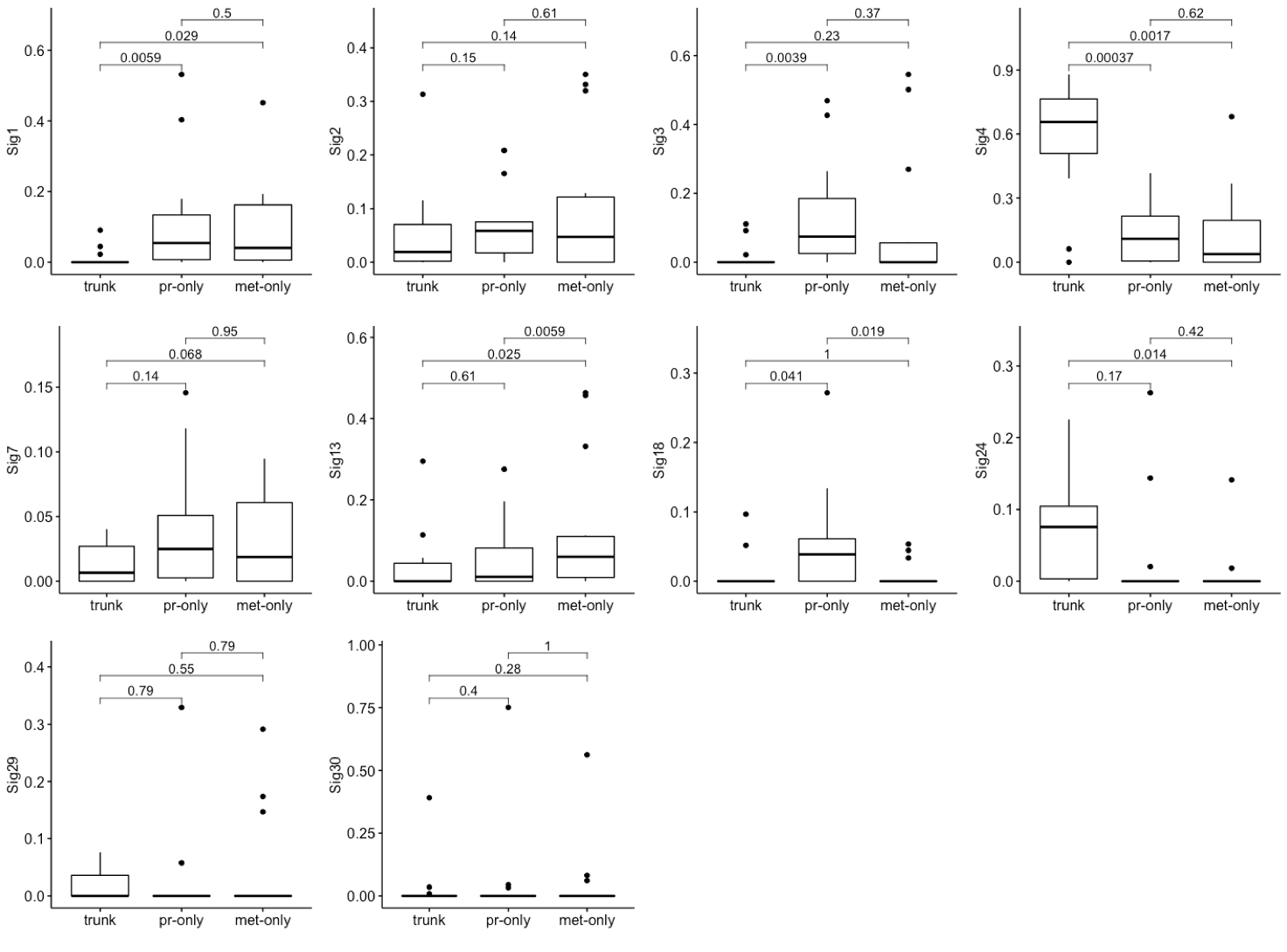


**Fig. S9. Comparison of the contributions to trunk, primary-only and metastasis-only mutations by mutational signature**

The contributions of the top 10 mutational signatures to mutations shared by primary tumors and metastases (trunk), only detected in primary tumors (pr-only), or only detected in metastases (met-only) were compared. The difference was assessed by Wilcoxon signed-rank test.


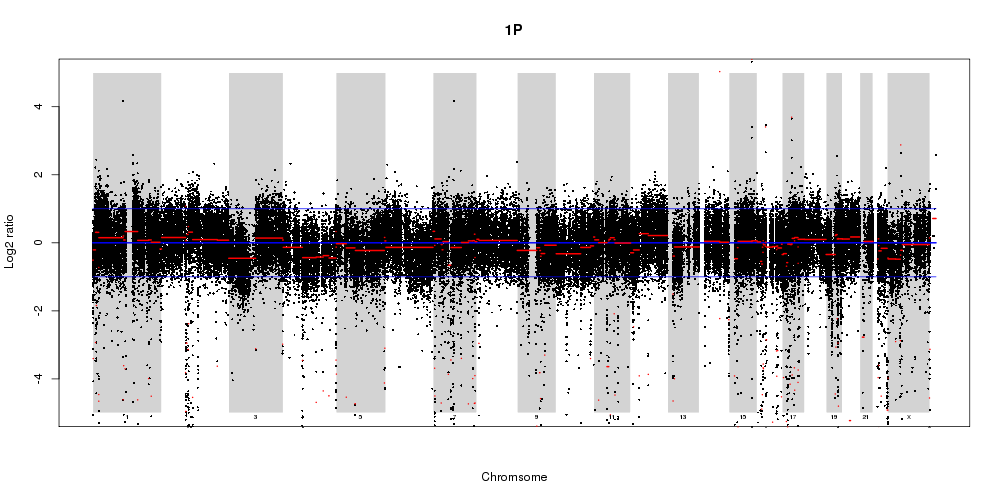

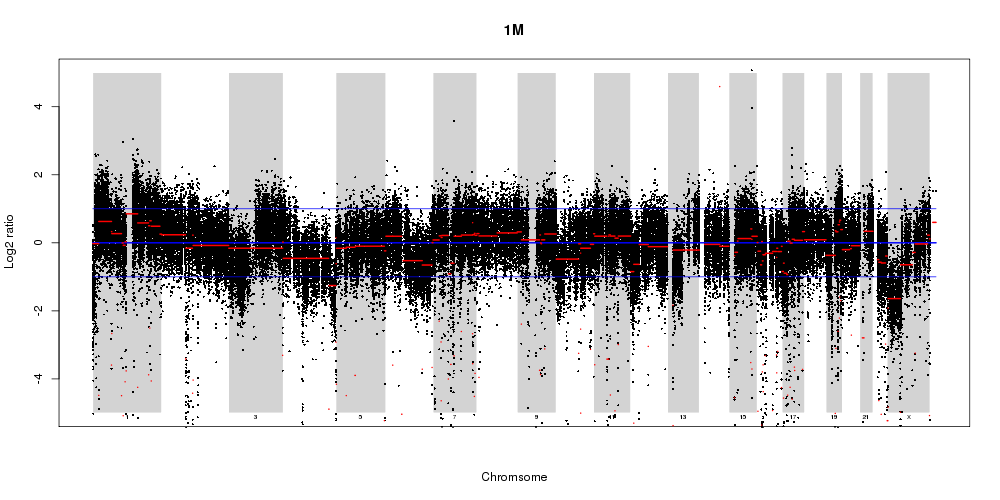


| **1P** | **1M** |
| --- | --- |


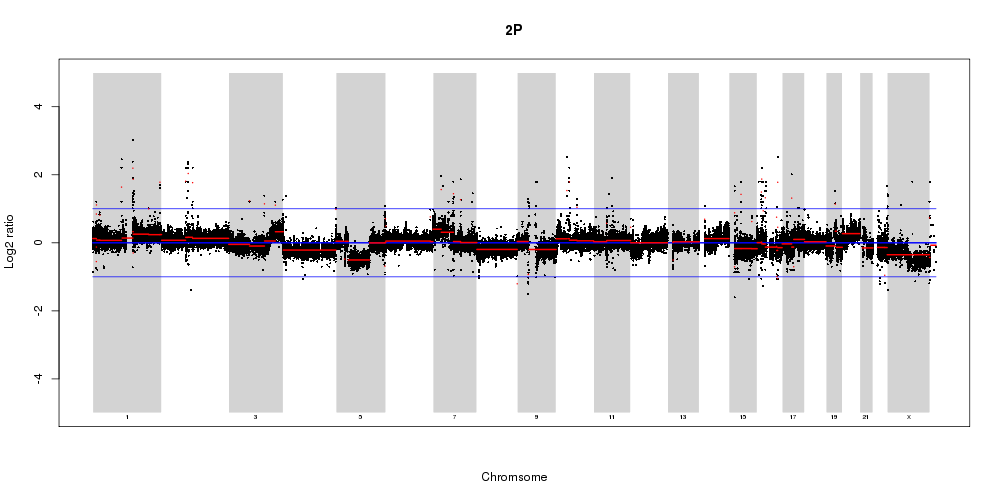

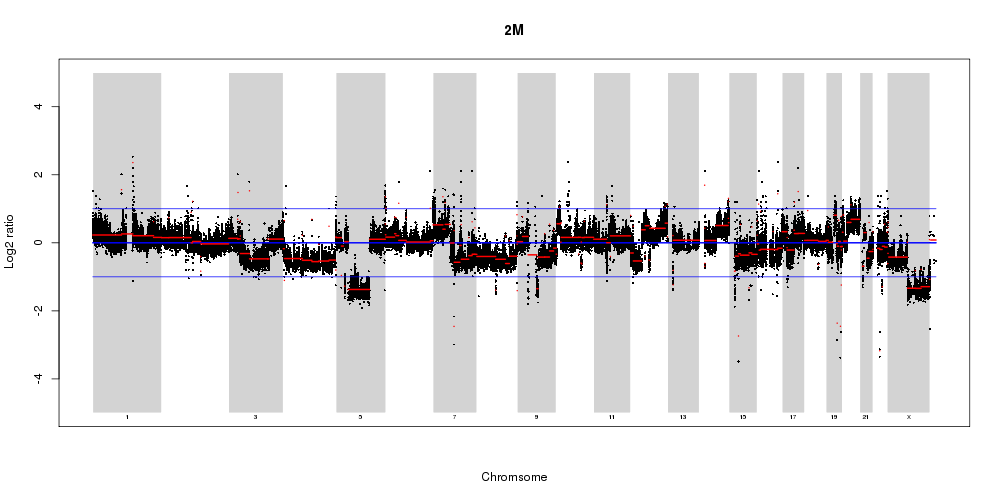


| **2P** | **2M** |
| --- | --- |


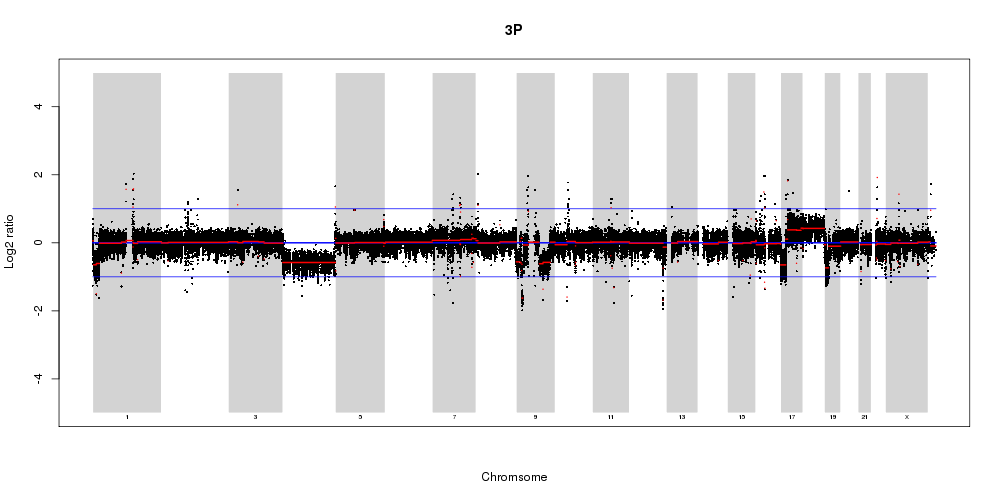

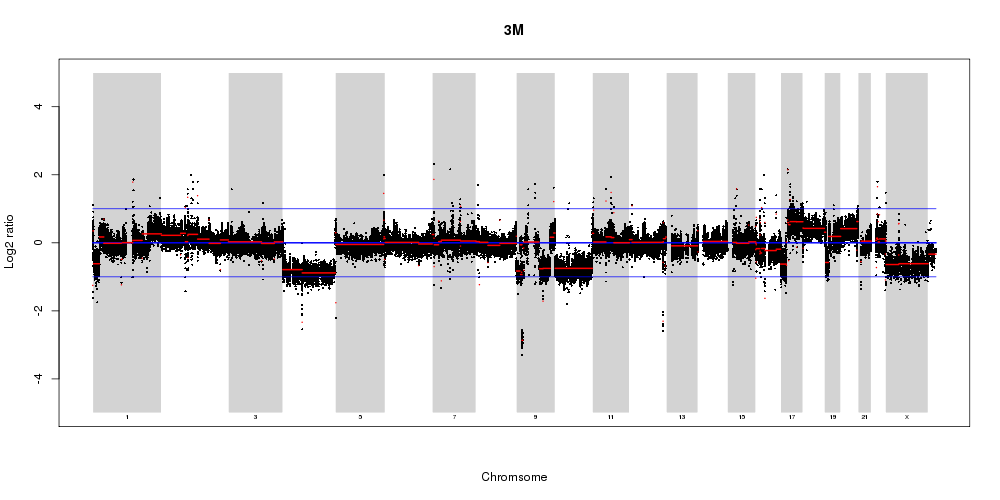


| **3P** | **3M** |
| --- | --- |


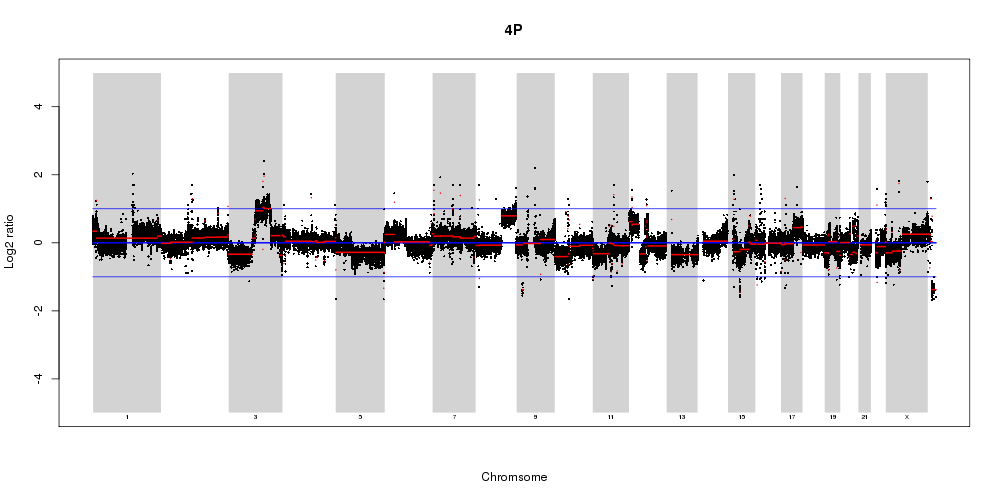

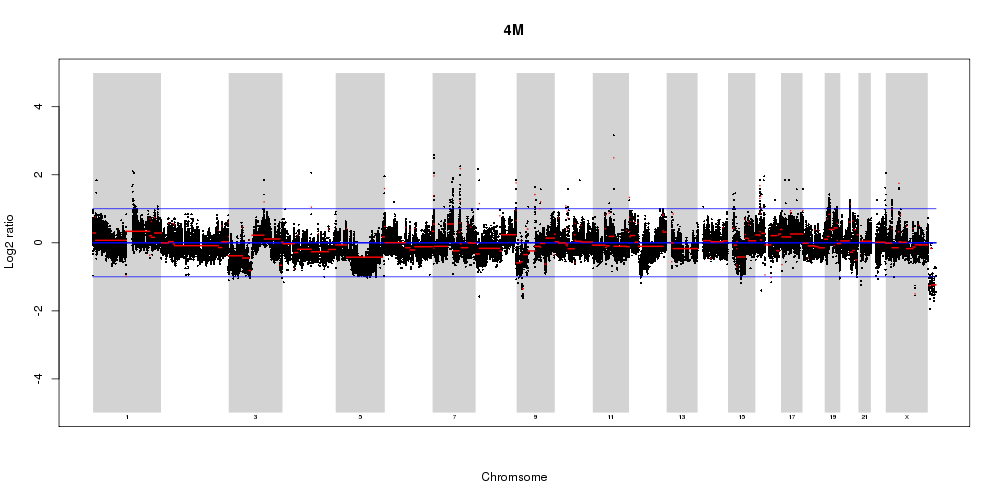


| **4P** | **4M** |
| --- | --- |

**
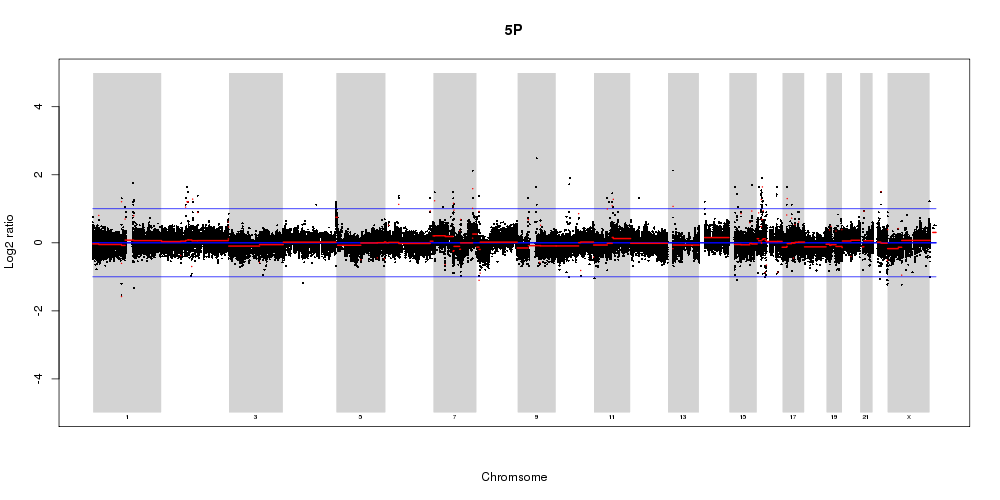

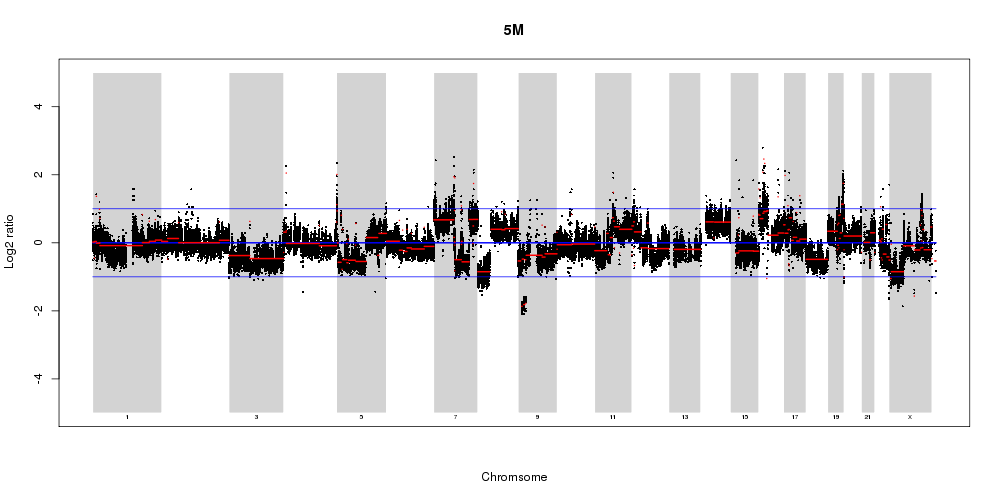
**

| **5P** | **5M** |
| --- | --- |

**
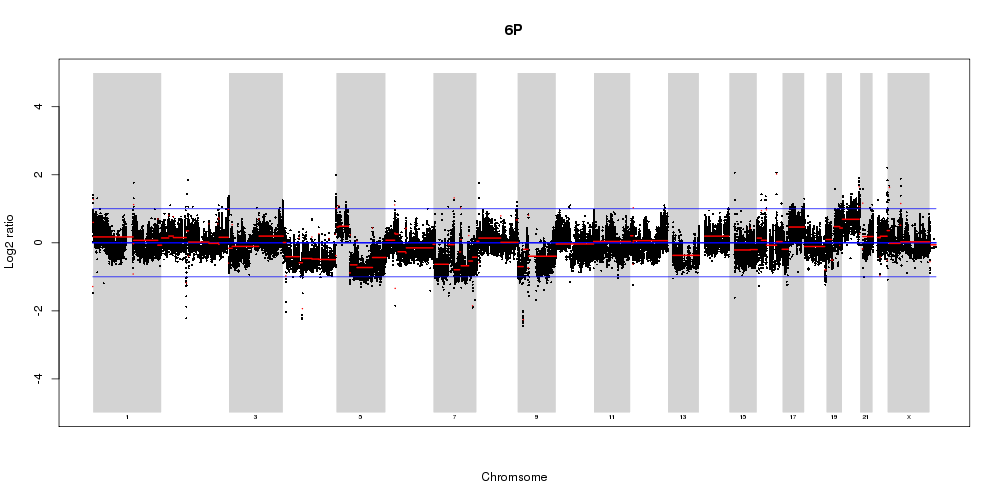

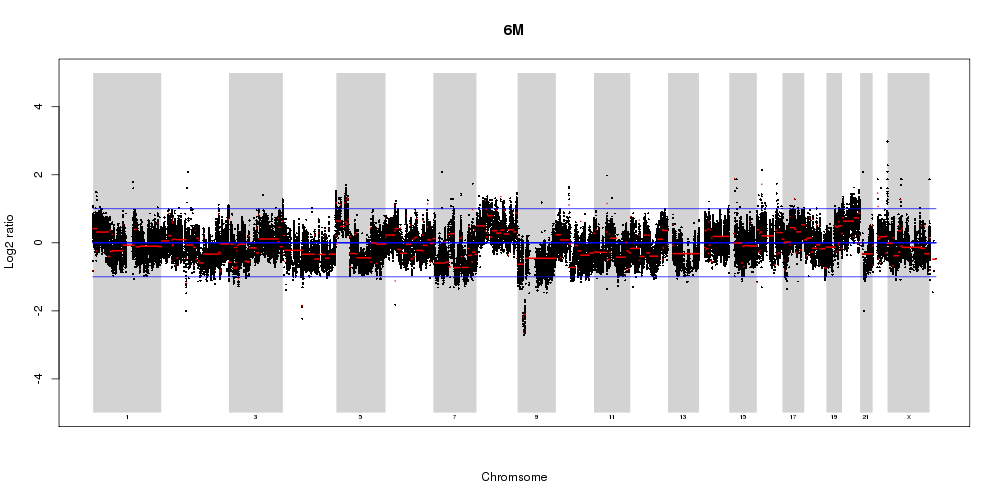
**

| **6P** | **6M** |
| --- | --- |

**
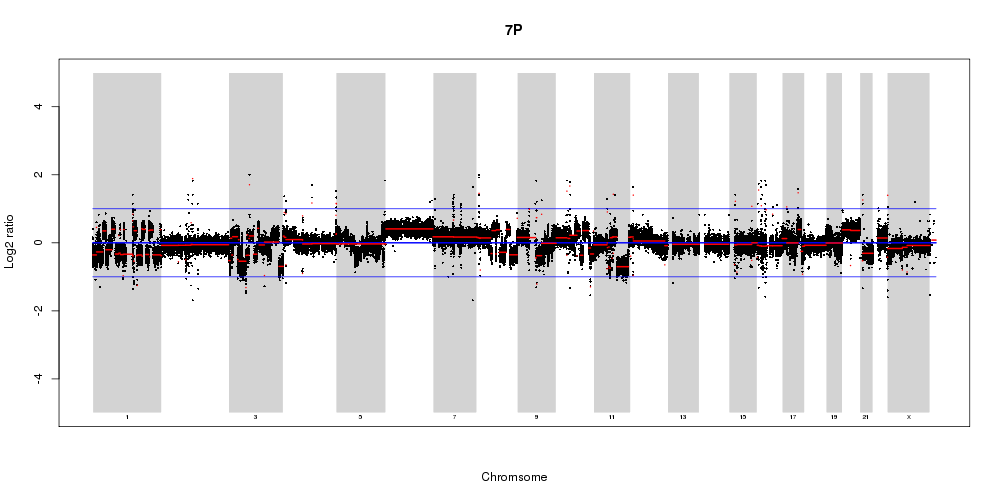

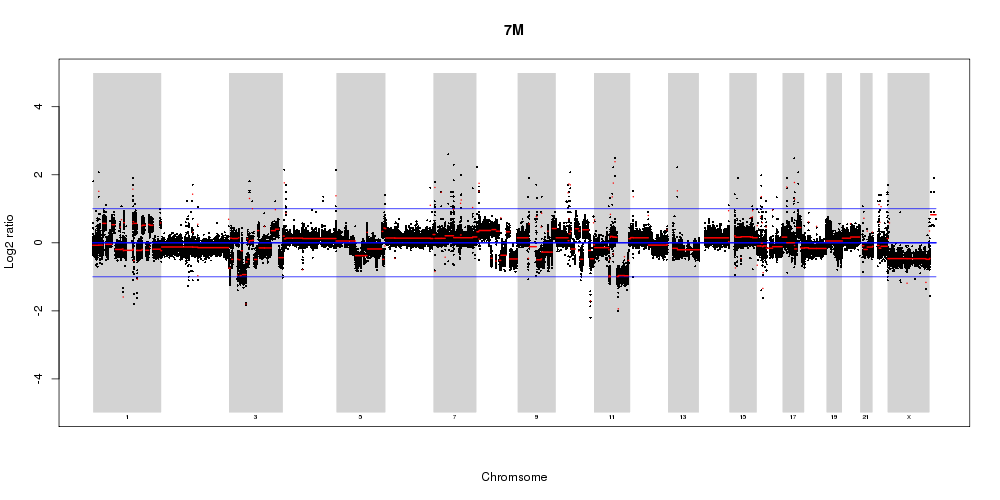
**

| **7P** | **7M** |
| --- | --- |

**
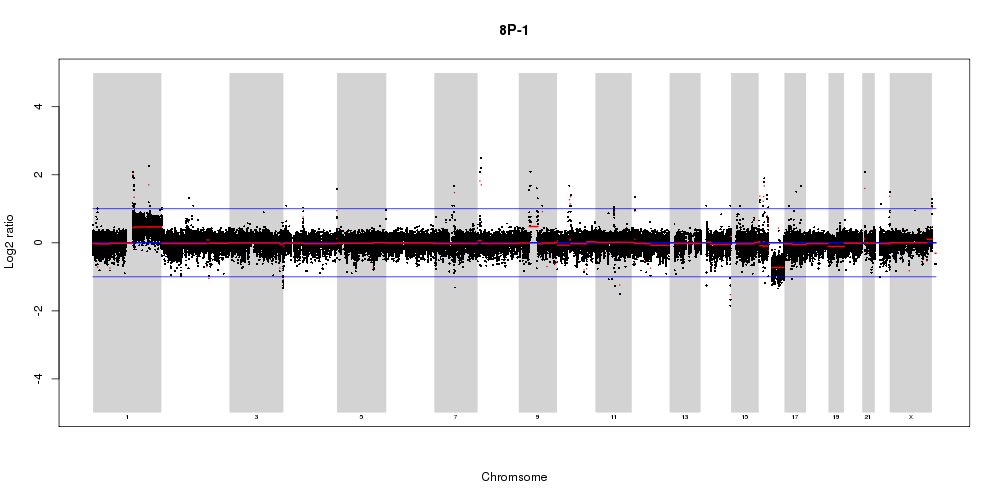

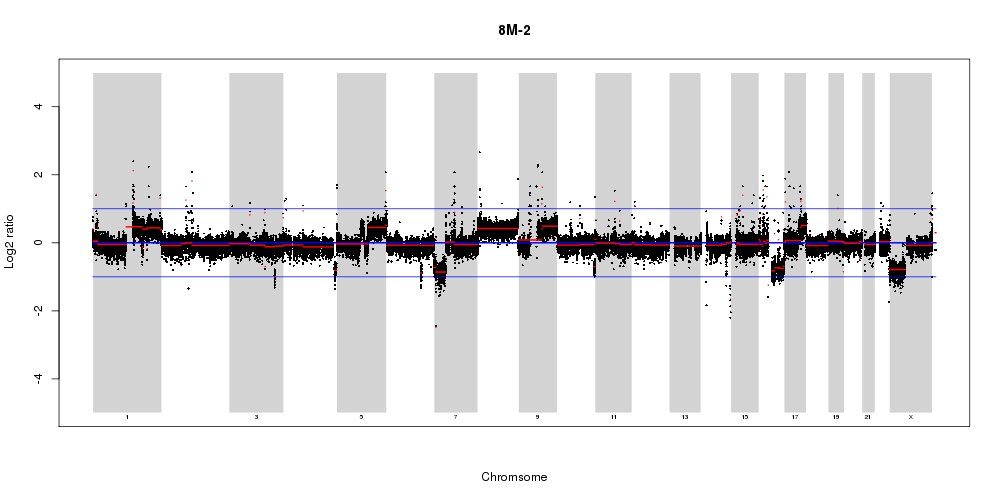
**

| **8P-1** | **8M-2** |
| --- | --- |


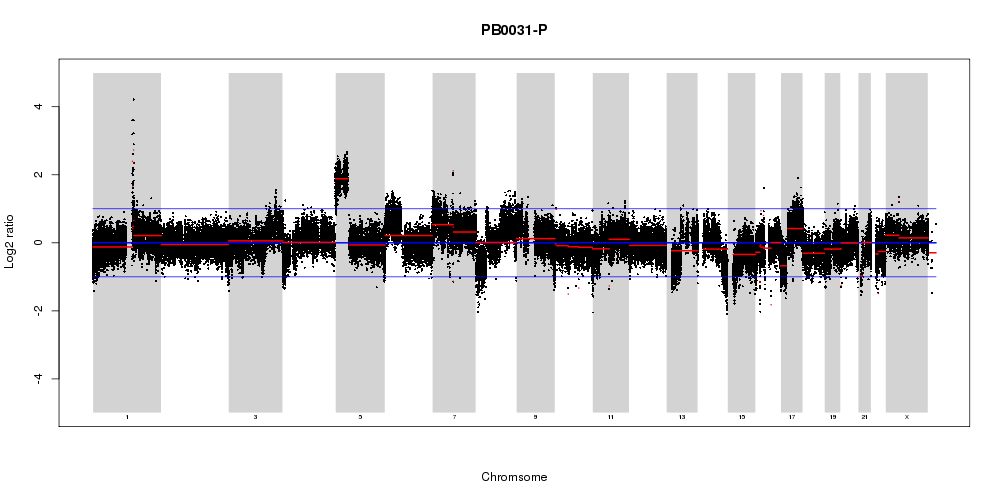

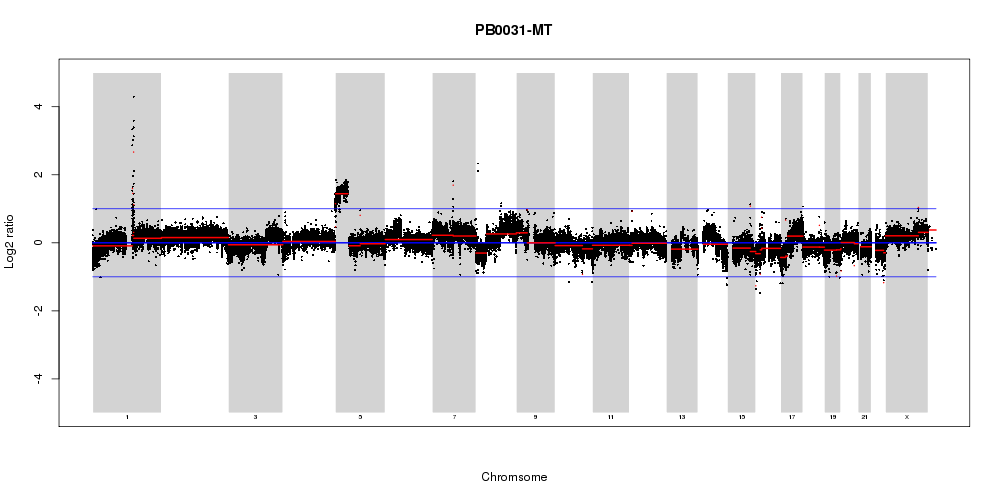


| **PB0031-P** | **PB0031-MT** |
| --- | --- |


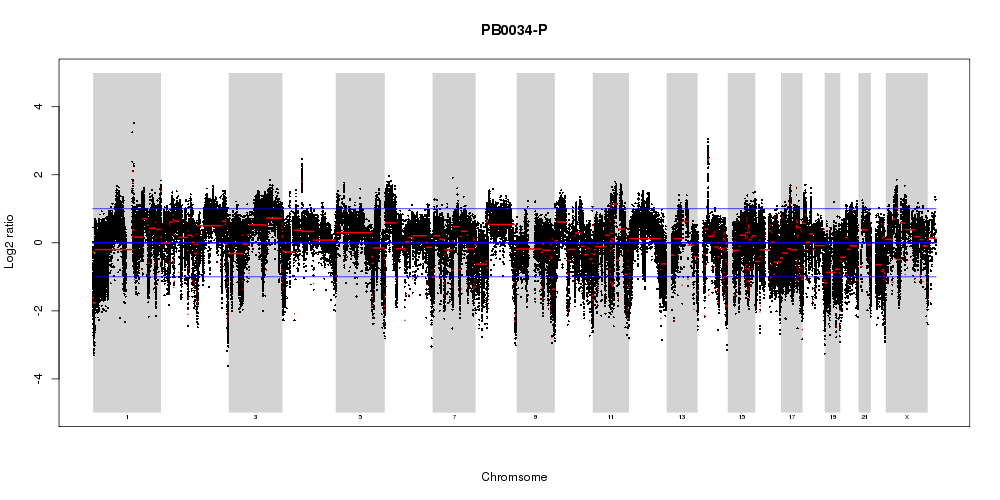

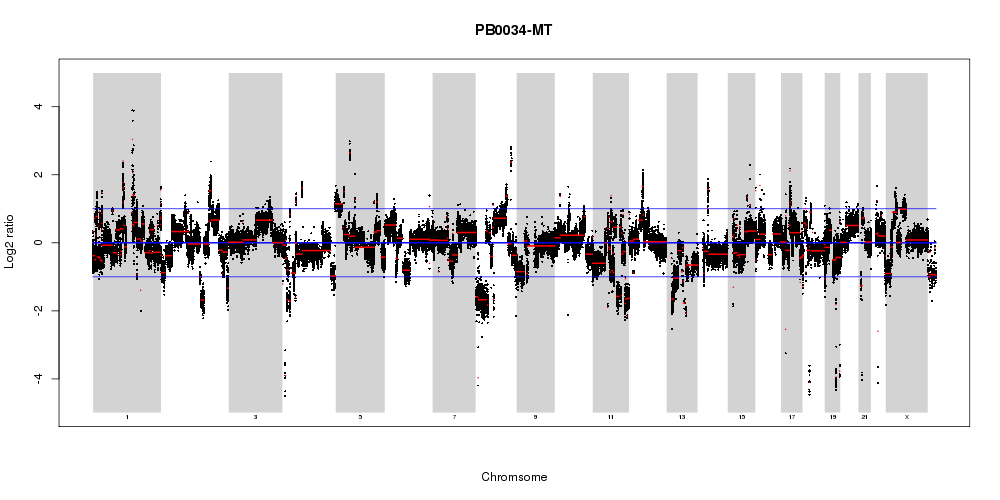


| **PB0034-P** | **PB0034-MT** |
| --- | --- |


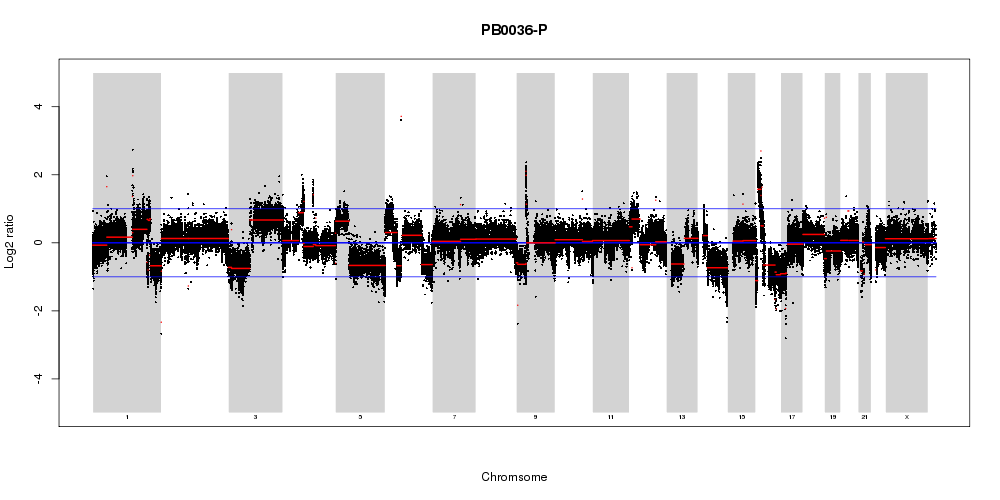

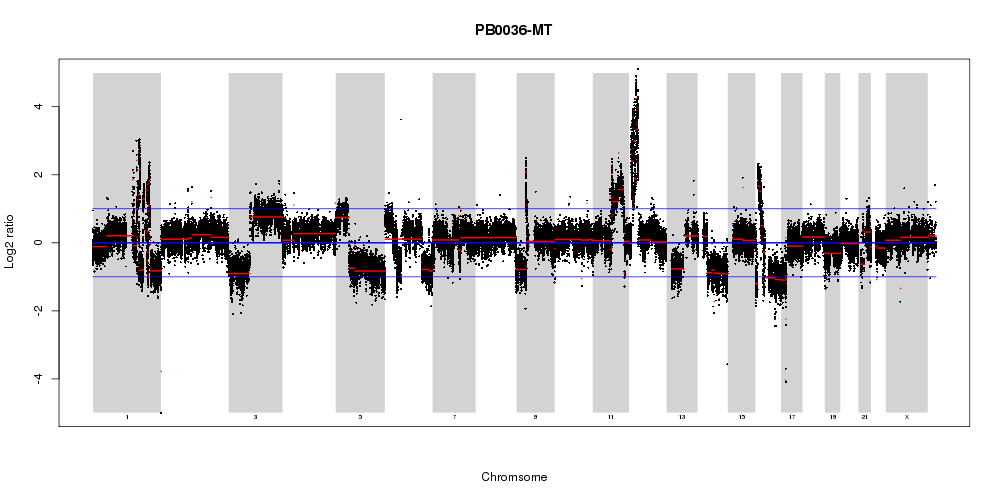


| **PB0036-P** | **PB0036-MT** |
| --- | --- |


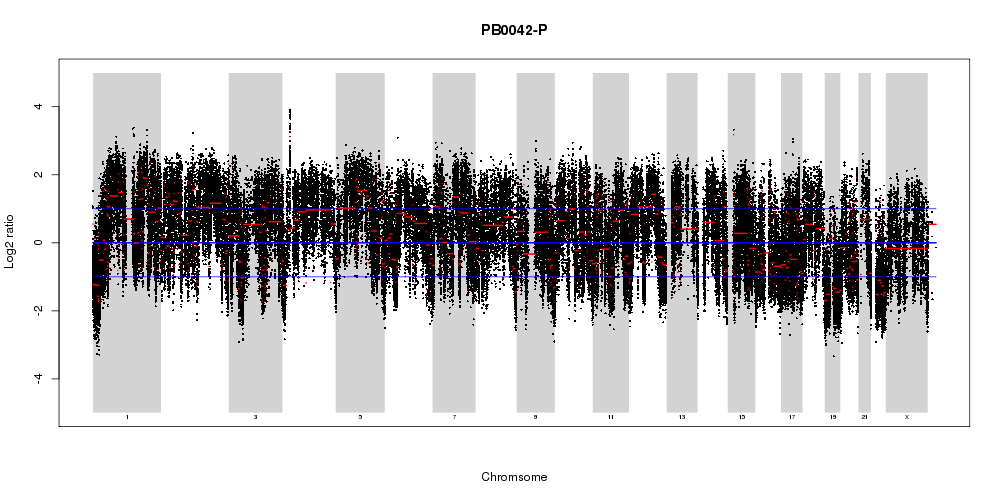

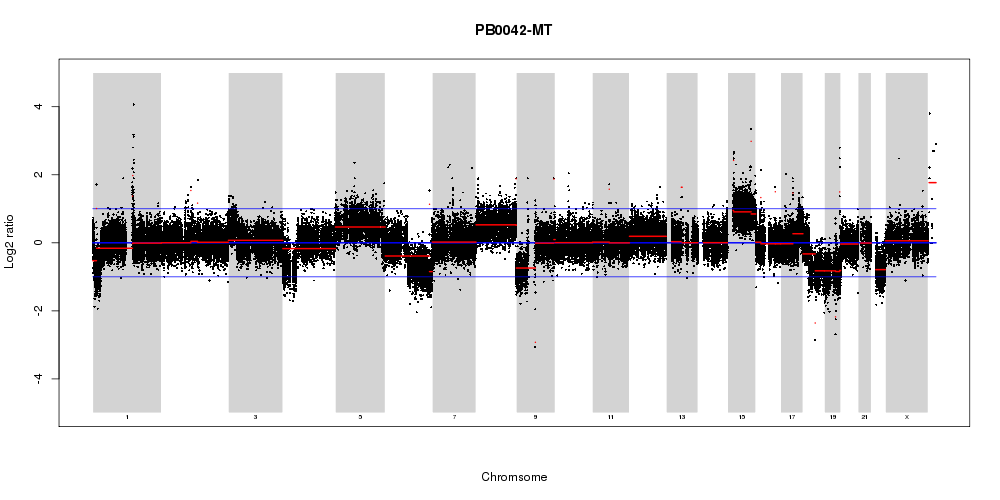


| **PB0042-P** | **PB0042-MT** |
| --- | --- |


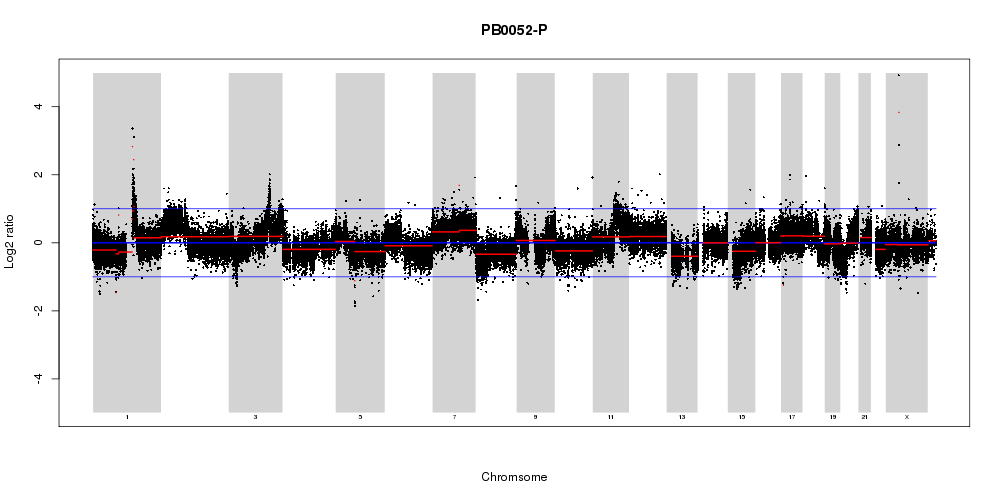

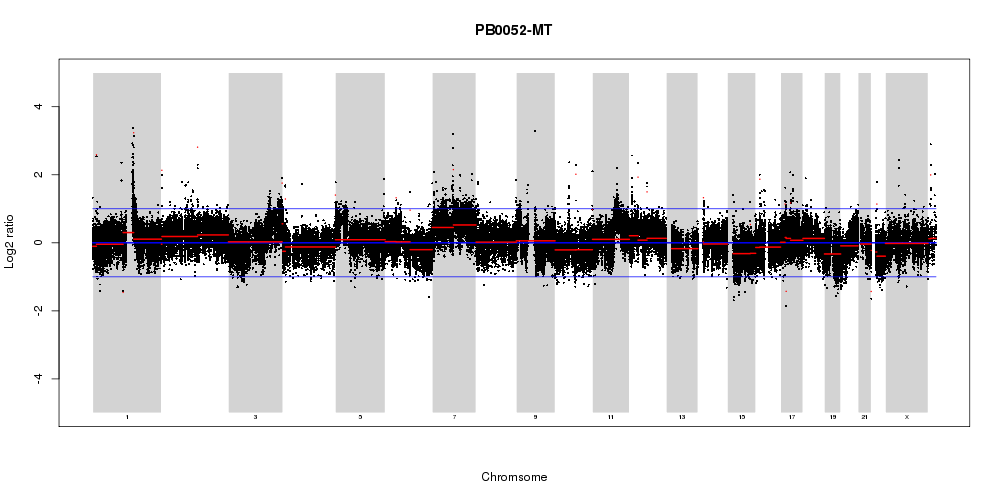


| **PB0052-P** | **PB0052-MT** |
| --- | --- |

**
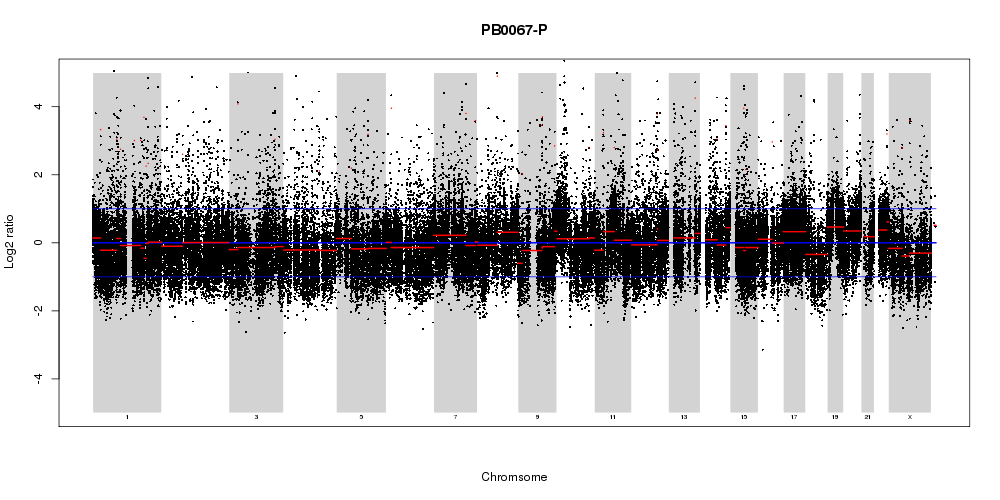
**
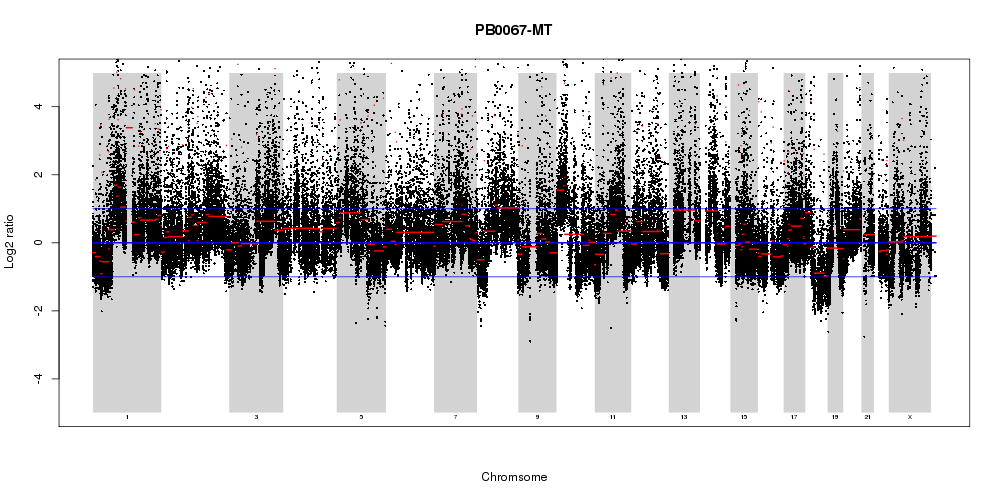


| **PB0067-P** | **PB0067-MT** |
| --- | --- |


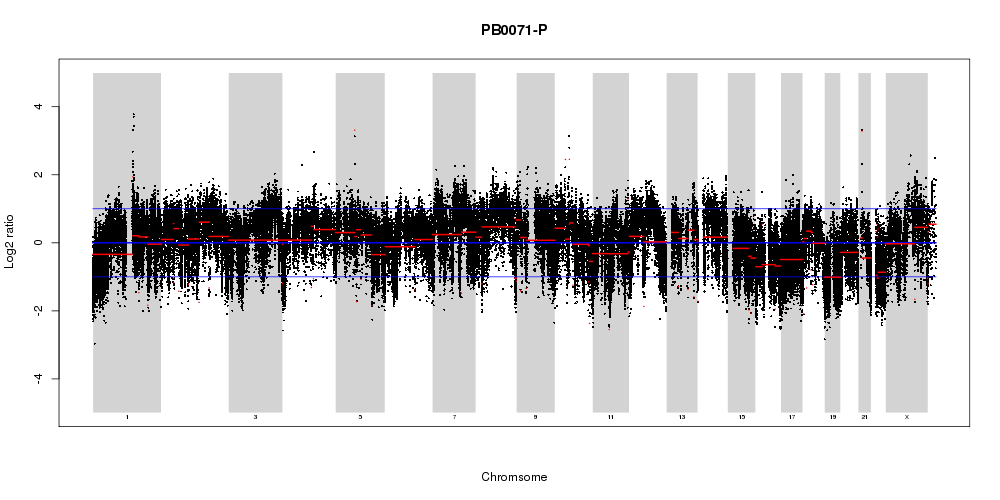

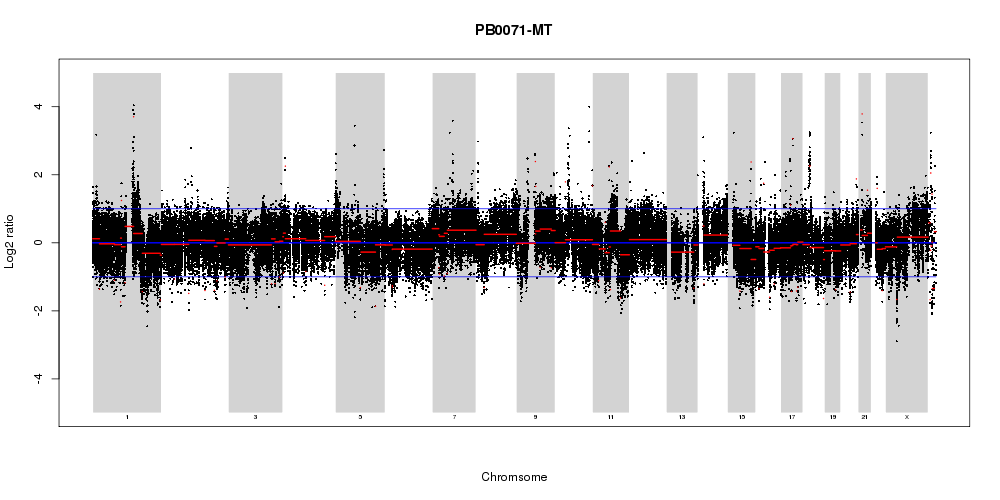


| **PB0071-P** | **PB0071-MT** |
| --- | --- |


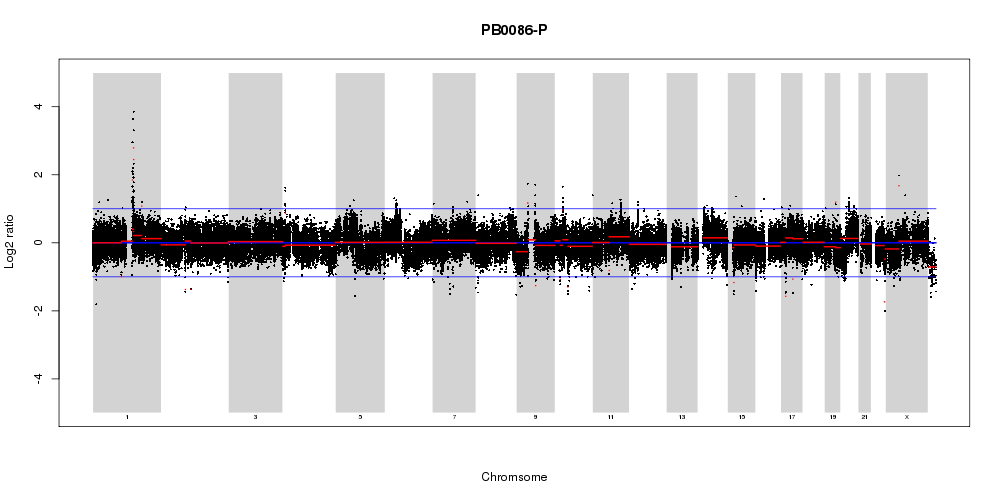

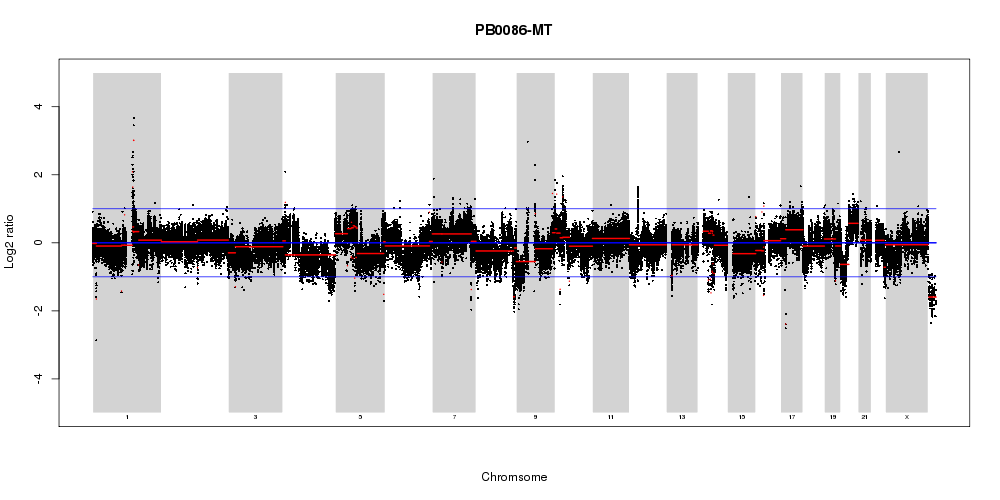


| **PB0086-P** | **PB0086-MT** |
| --- | --- |

**
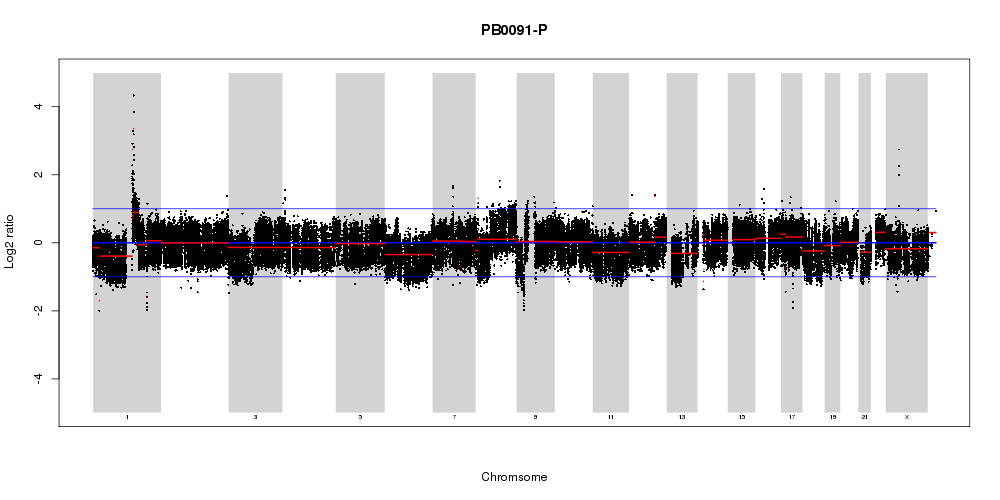

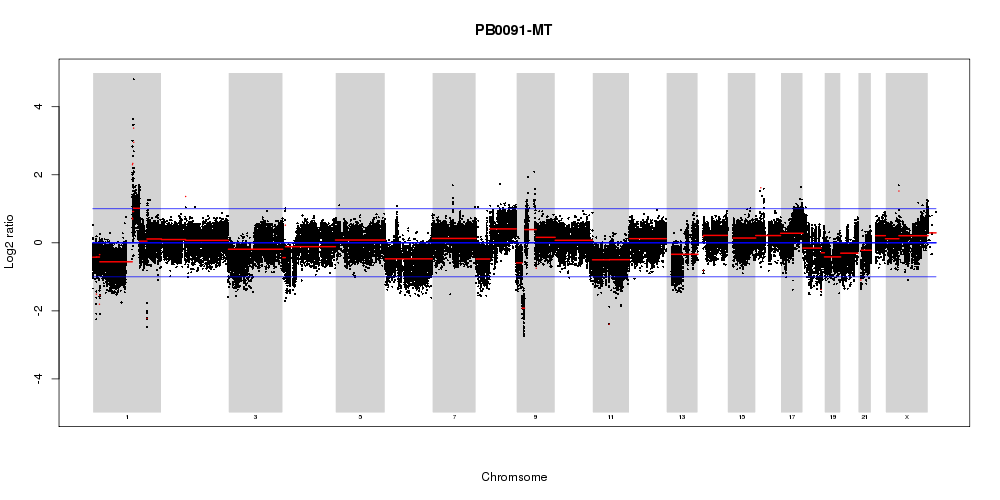
**

| **PB0091-P** | **PB0091-MT** |
| --- | --- |

**
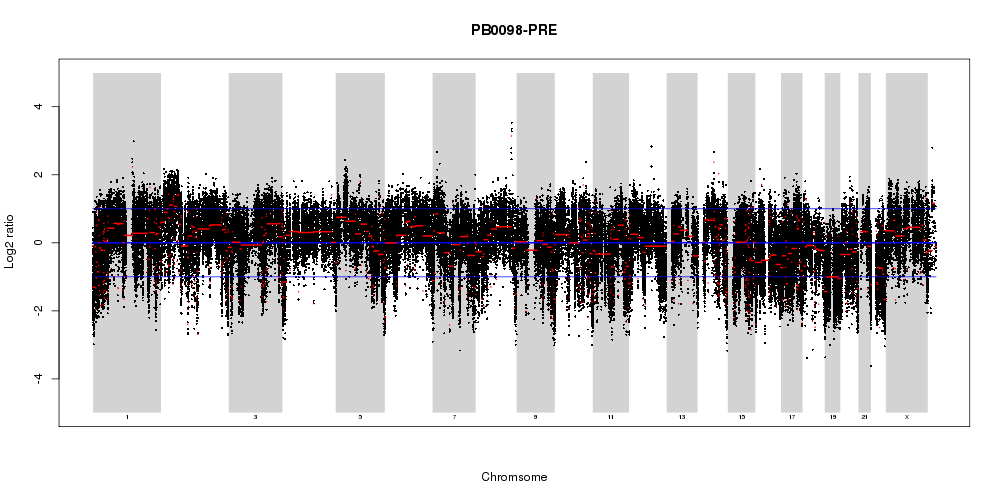

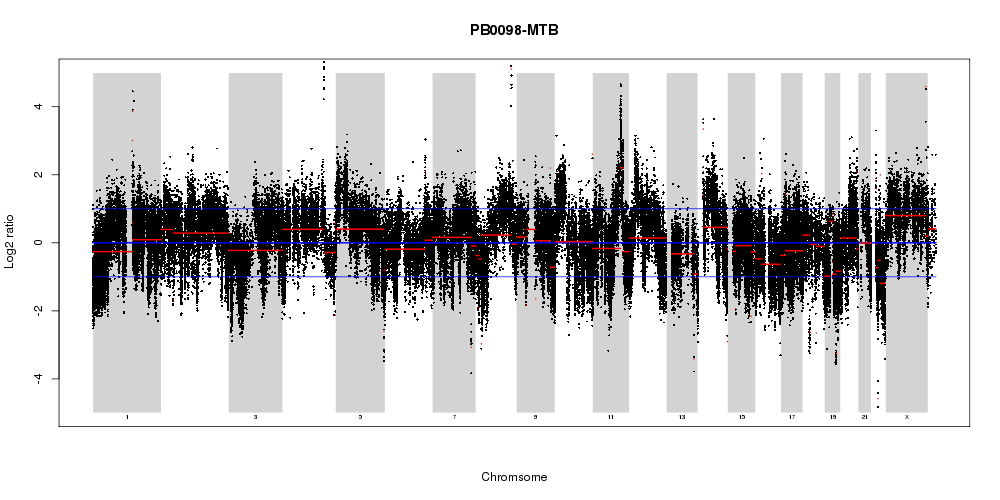
**

| **PB0098-PRE** | **PB0098-MTB** |
| --- | --- |

**
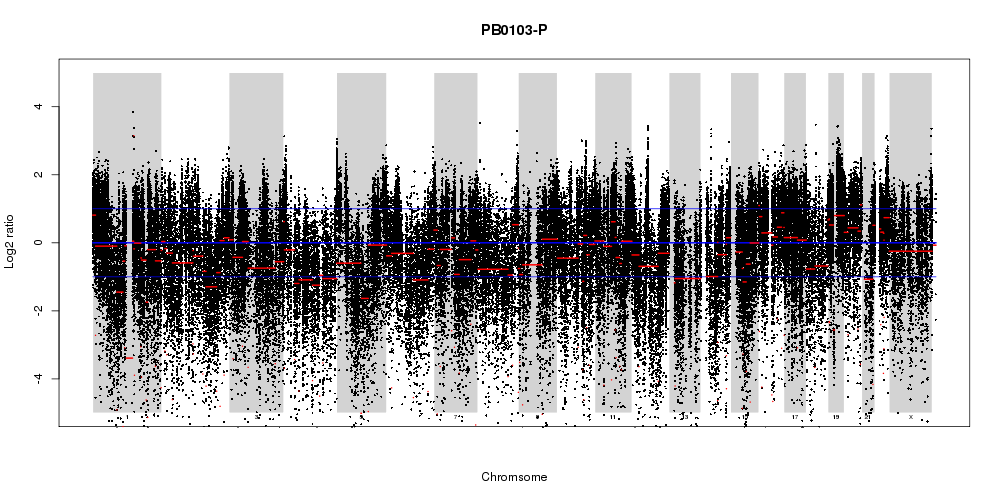

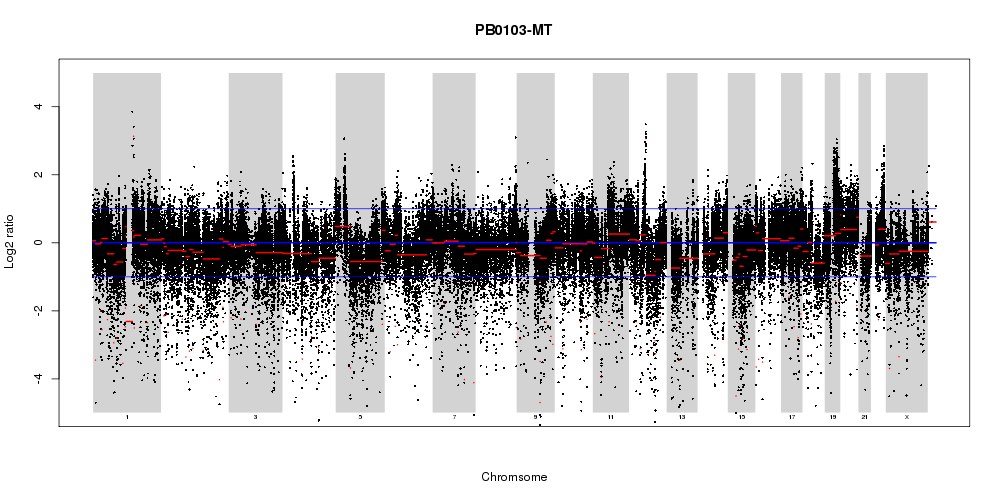
**

| **PB0103-P** | **PB0103-MT** |
| --- | --- |

**
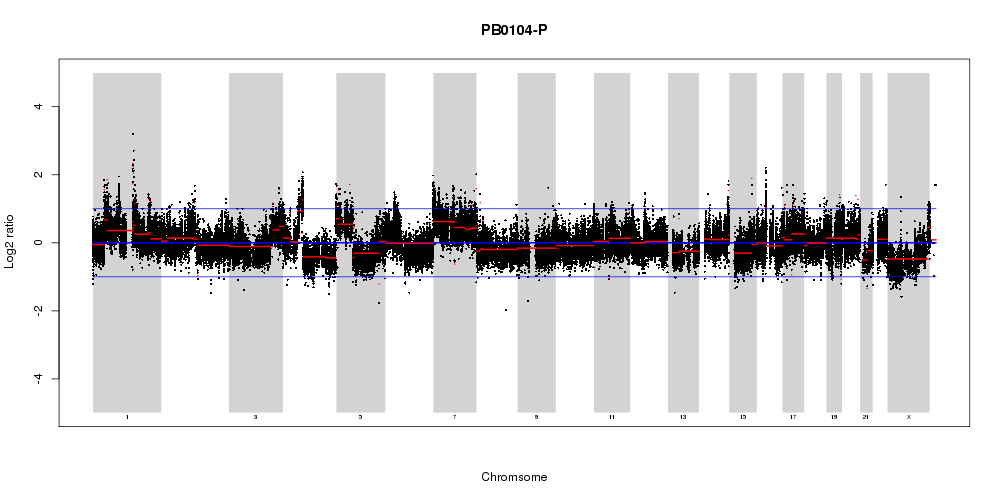
**
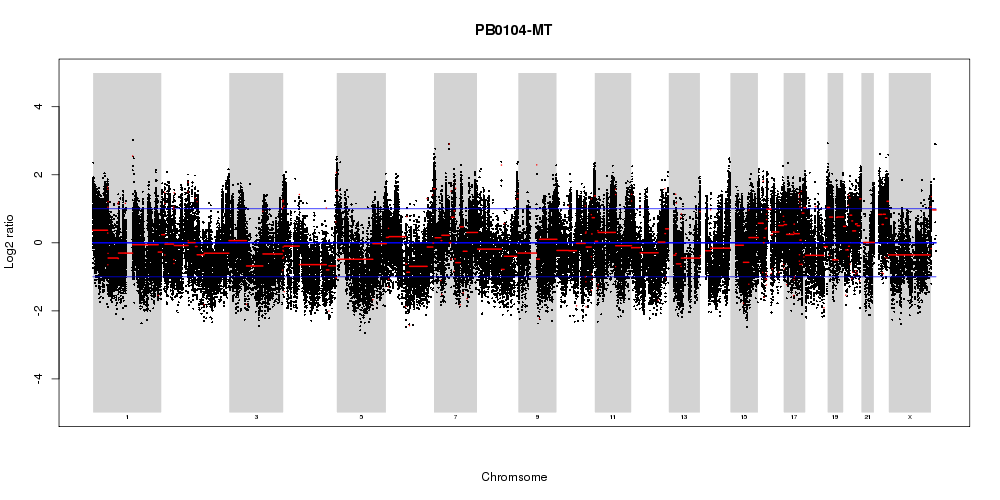


| **PB0104-P** | **PB0104-MT** |
| --- | --- |

**
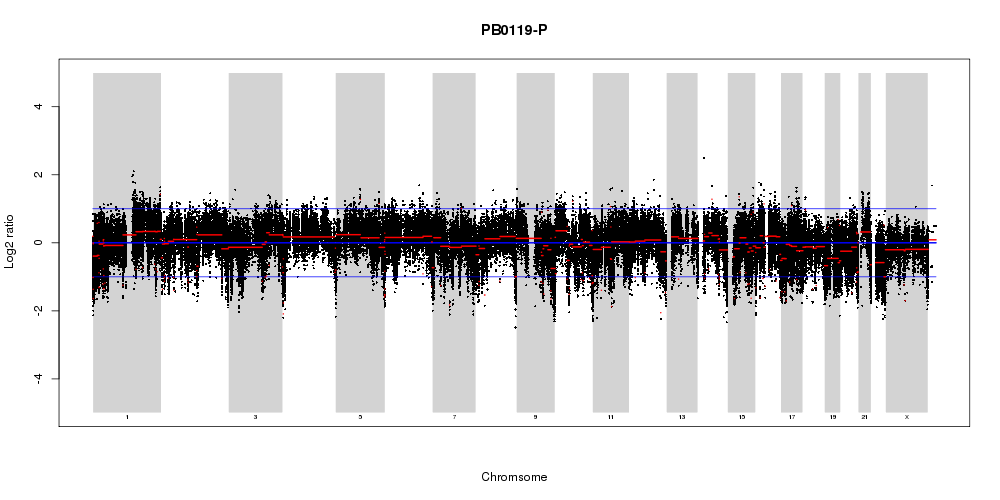

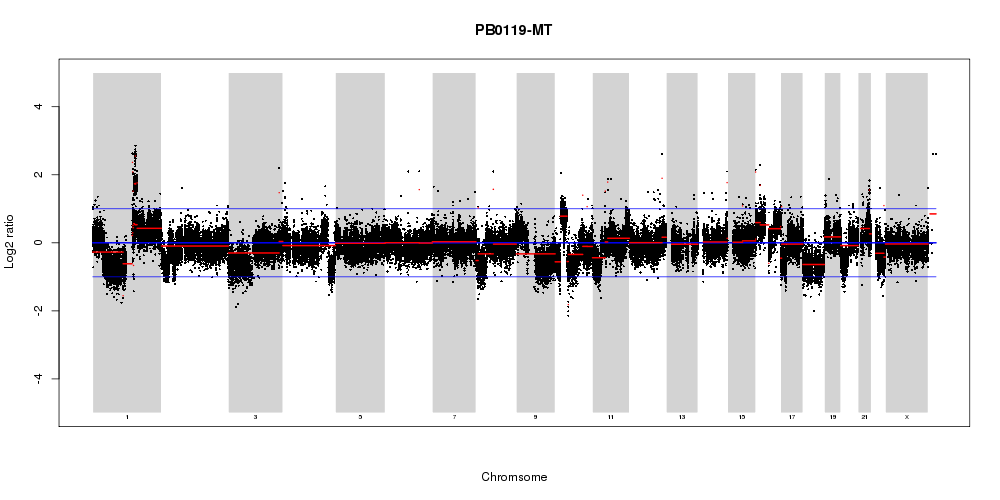
**

| **PB0119-P** | **PB0119-MT** |
| --- | --- |

**
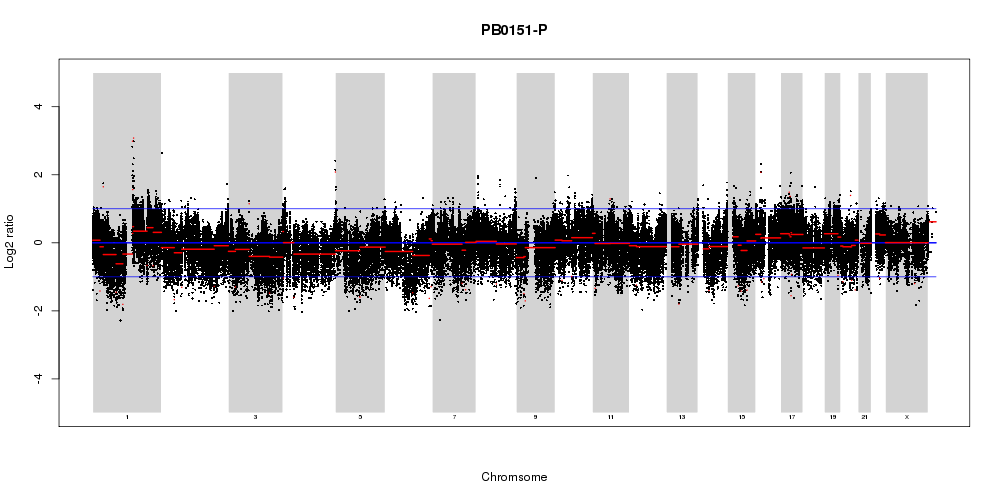

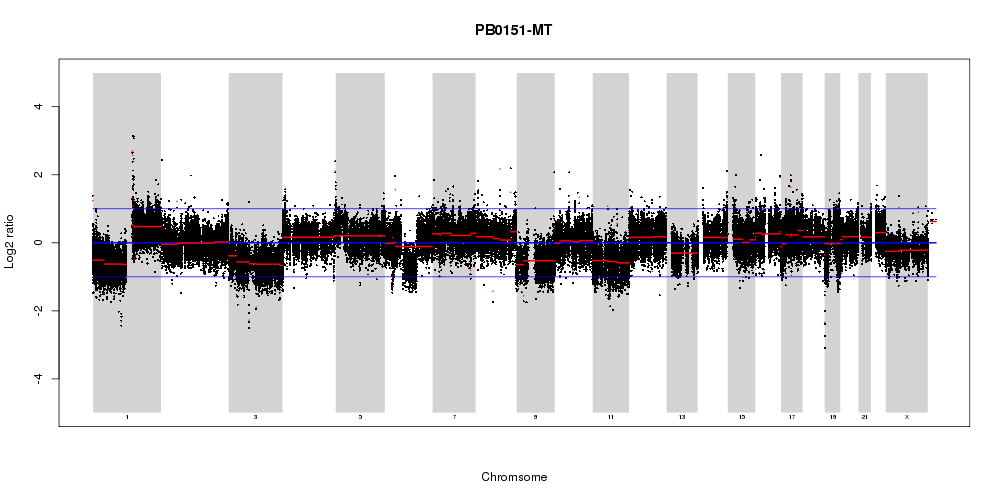
**

| **PB0151-P** | **PB0151-MT** |
| --- | --- |

**
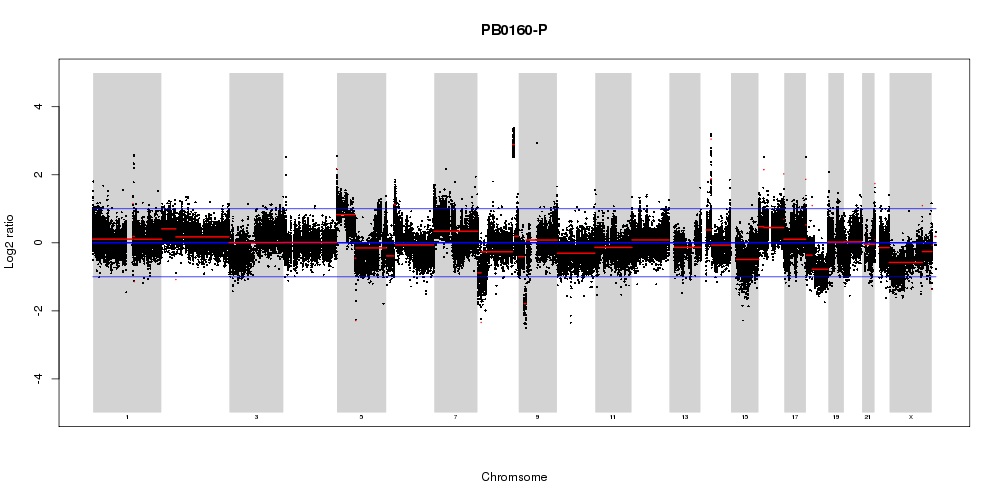
**
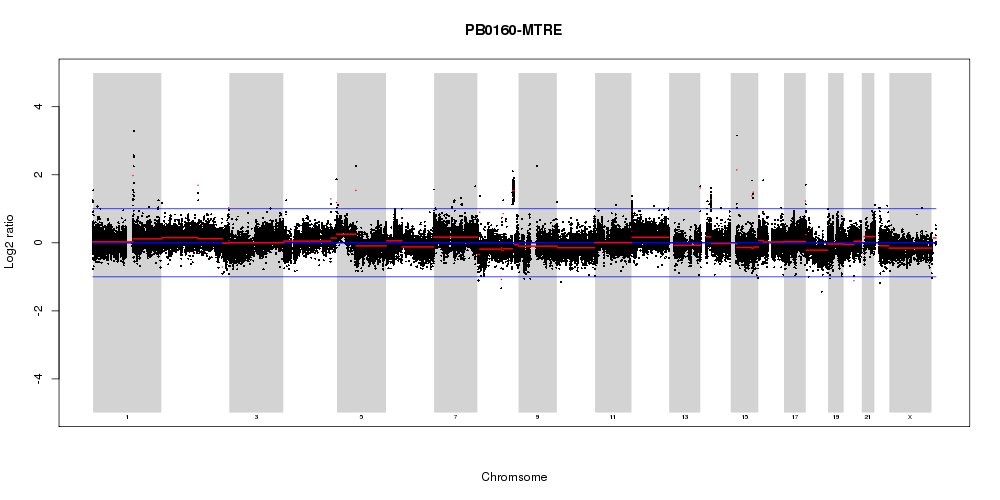


| **PB0160-P** | **PB0160-MTRE** |
| --- | --- |


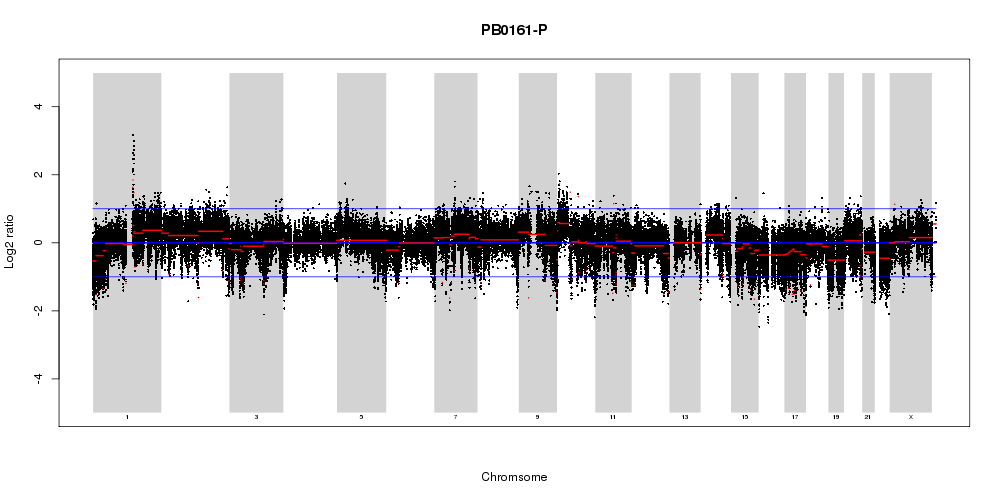

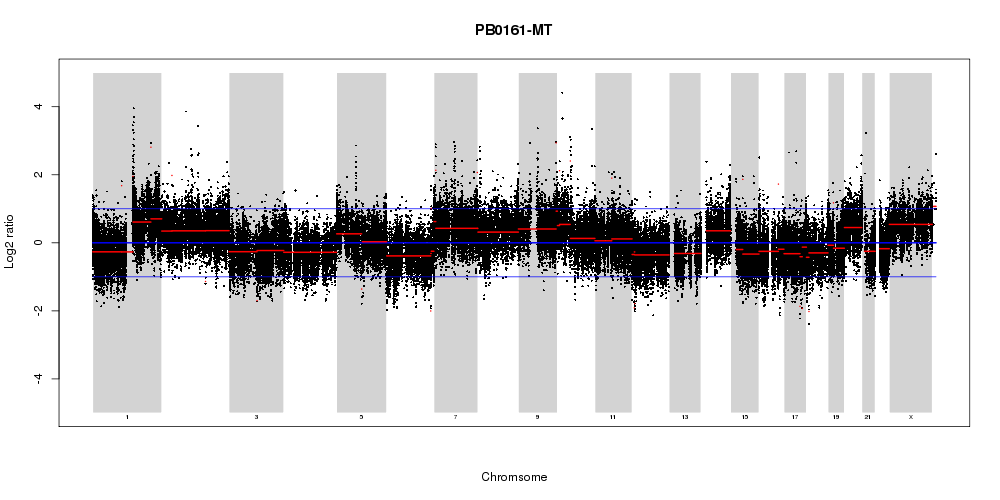


| **PB0161-P** | **PB0161-MT** |
| --- | --- |

**
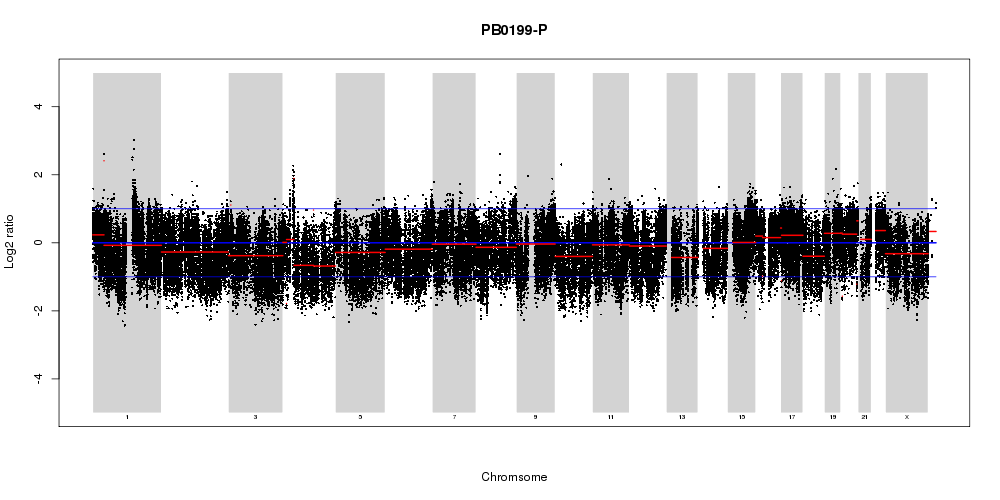
**
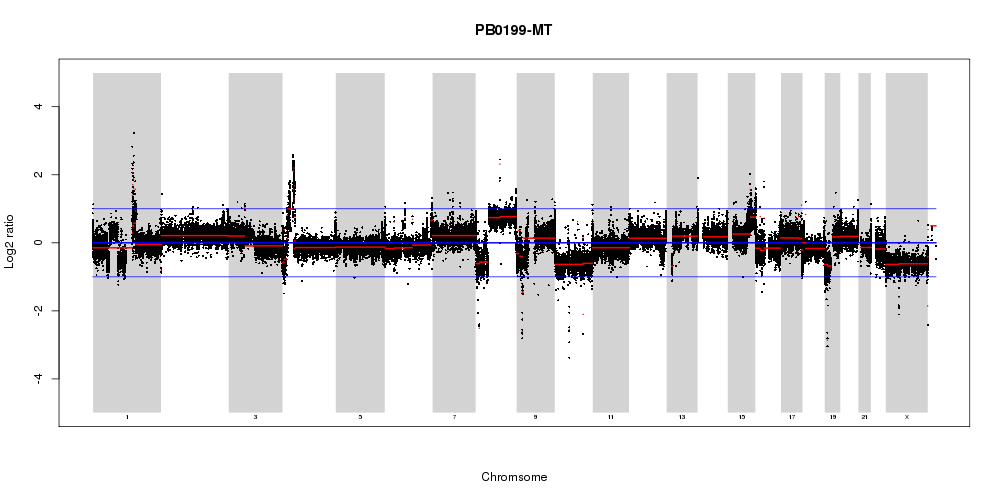


| **PB0199-P** | **PB0199-MT** |
| --- | --- |


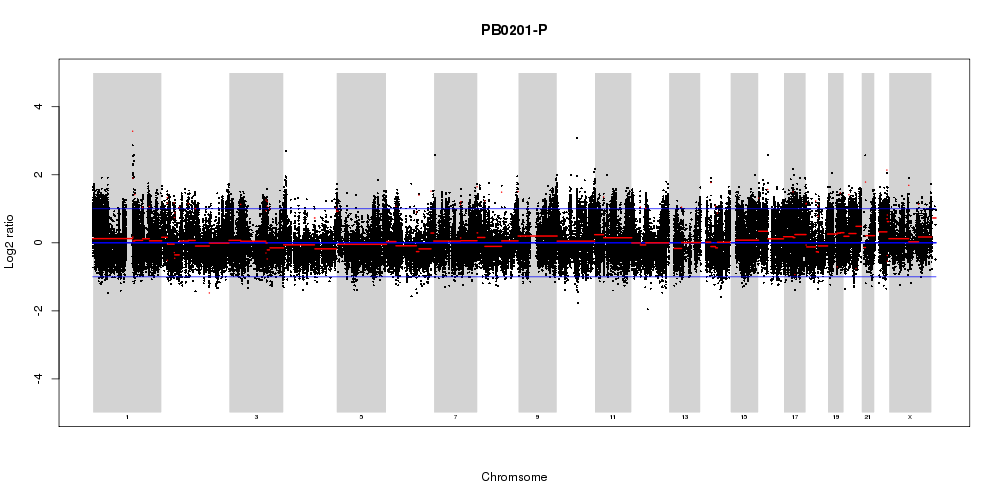

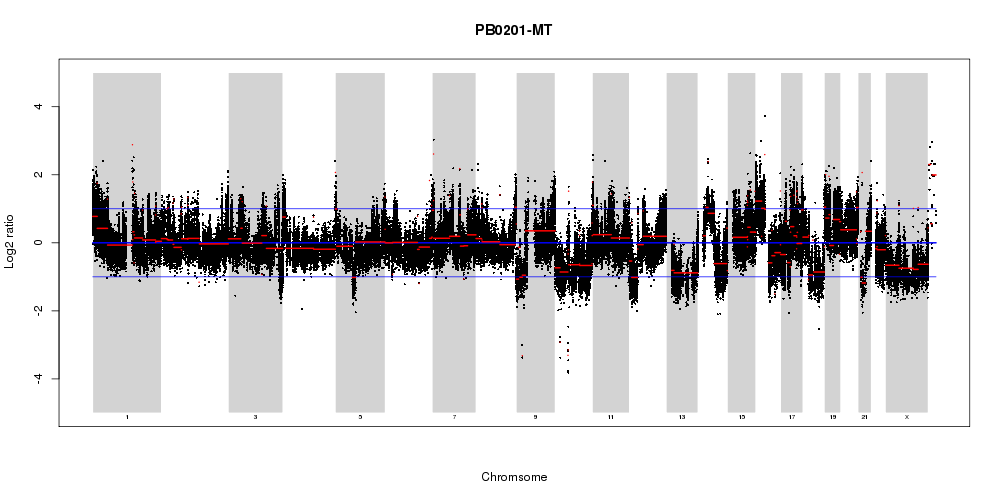


| **PB0201-P** | **PB0201-MT** |
| --- | --- |


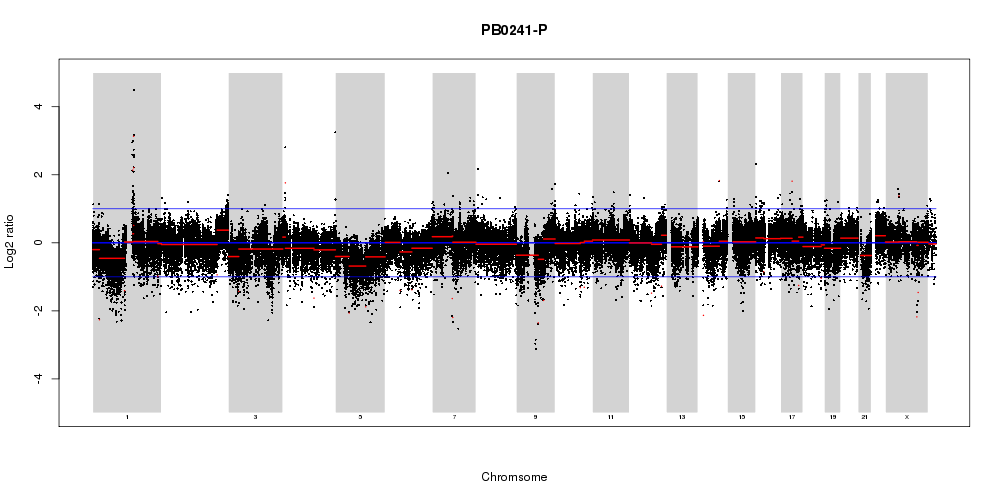

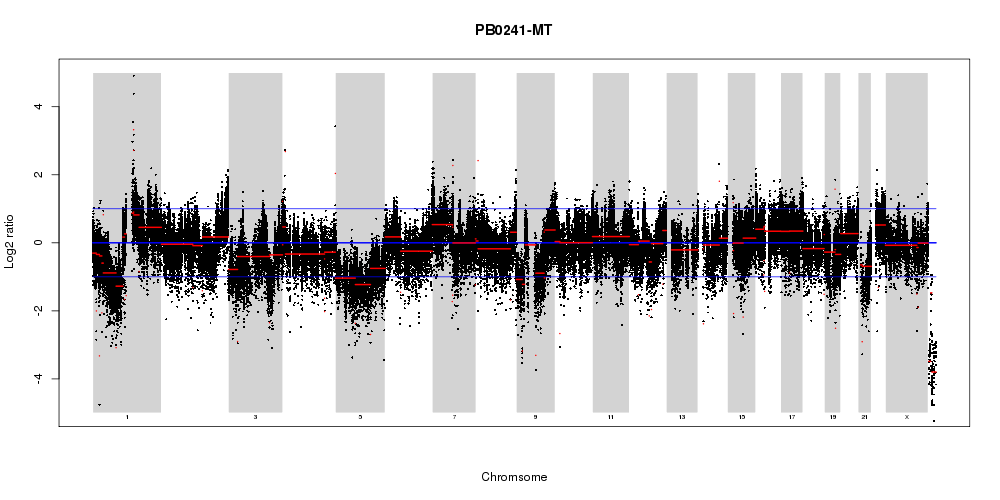


| **PB0241-P** | **PB0241-MT** |
| --- | --- |

**
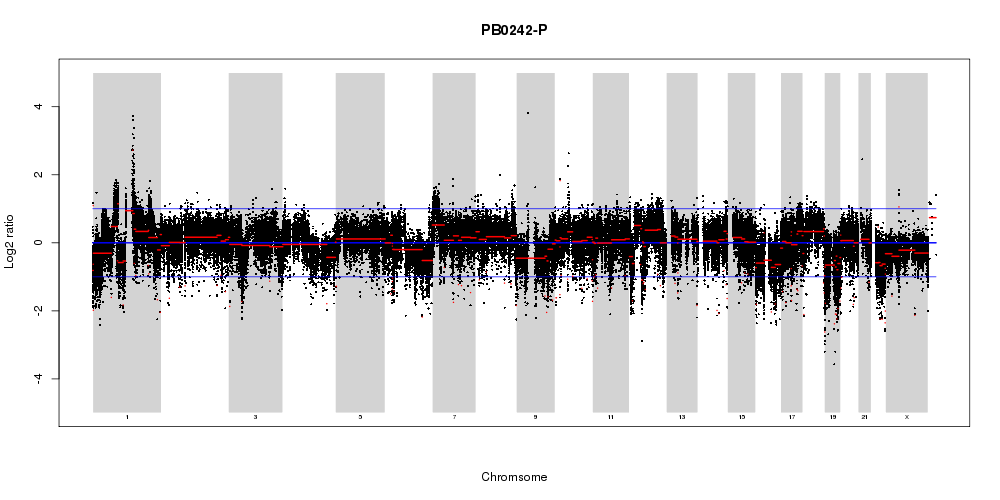
**
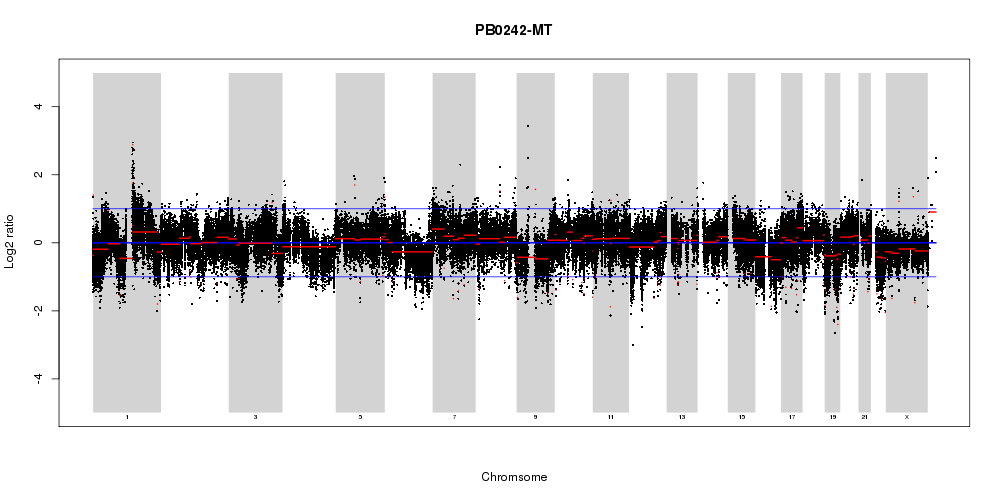


| **PB0242-P** | **PB0242-MT** |
| --- | --- |

**
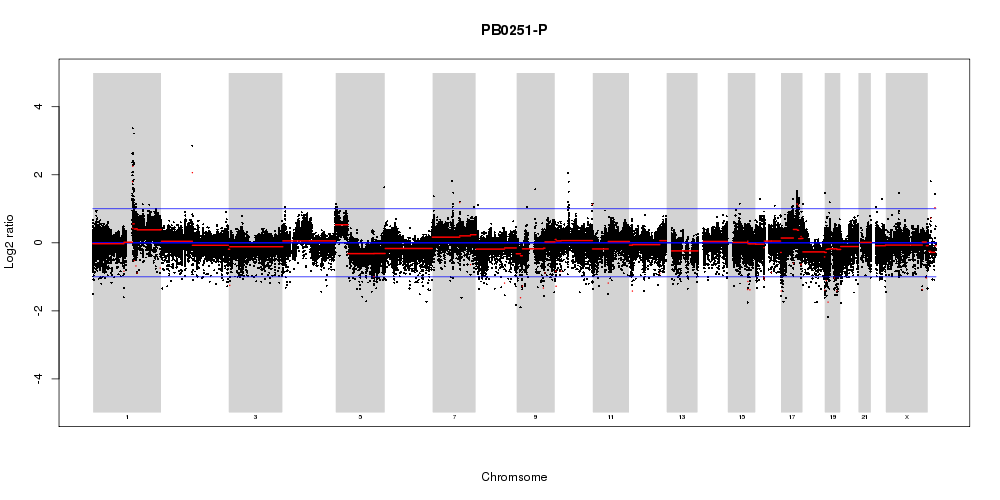
**
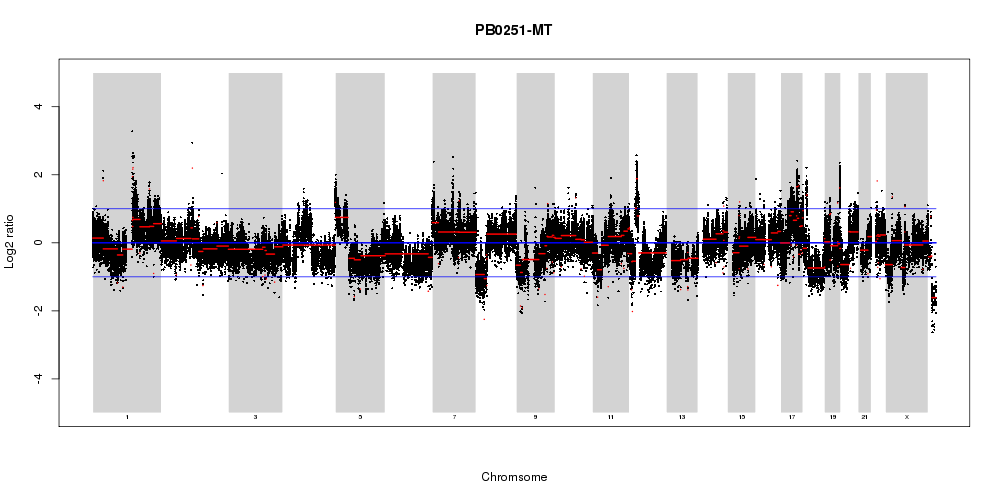


| **PB0251-P** | **PB0251-MT** |
| --- | --- |


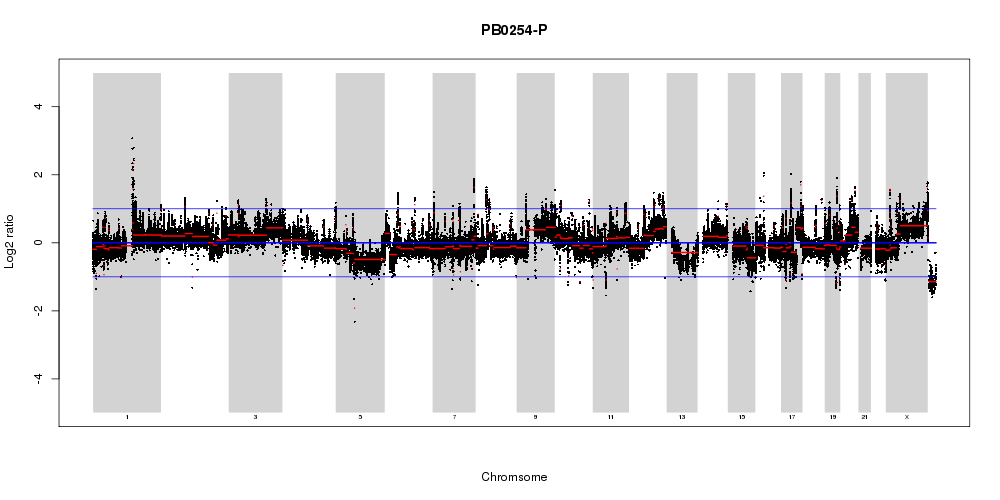

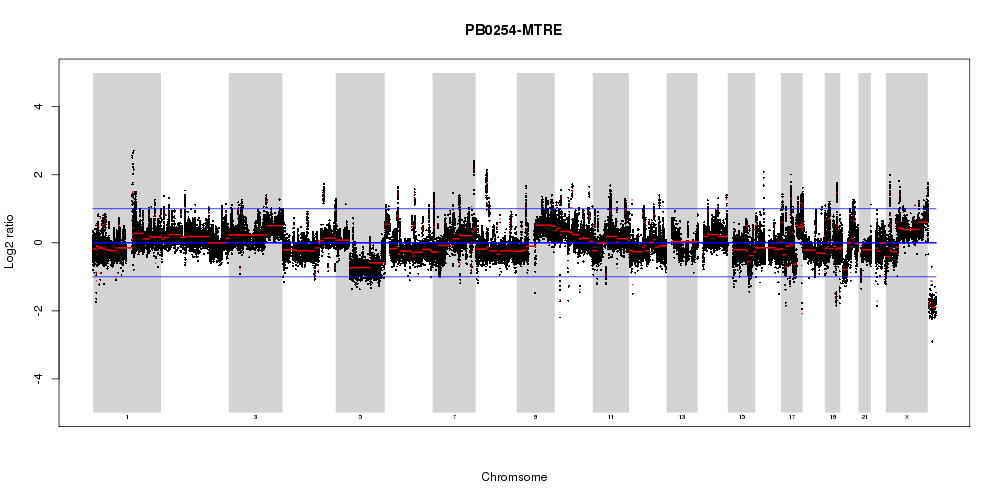


| **PB0254-P** | **PB0254-MTRE** |
| --- | --- |

**
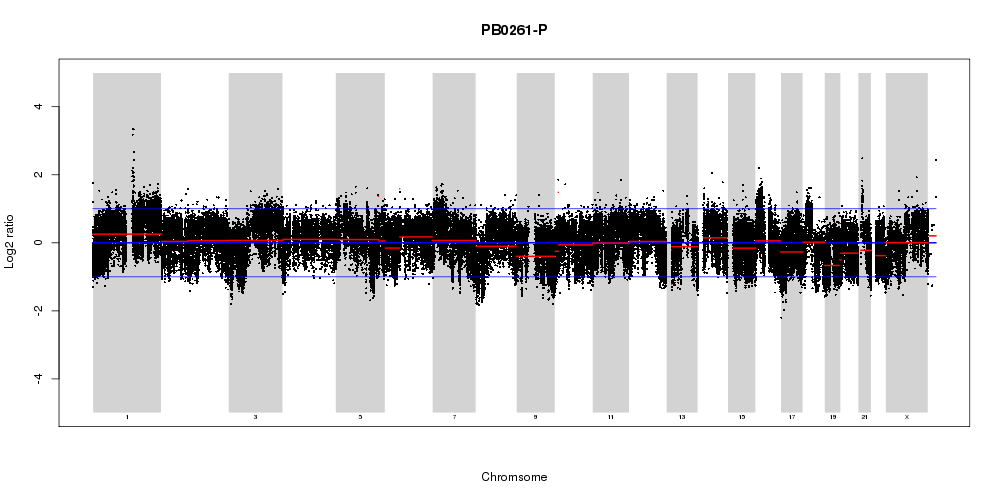

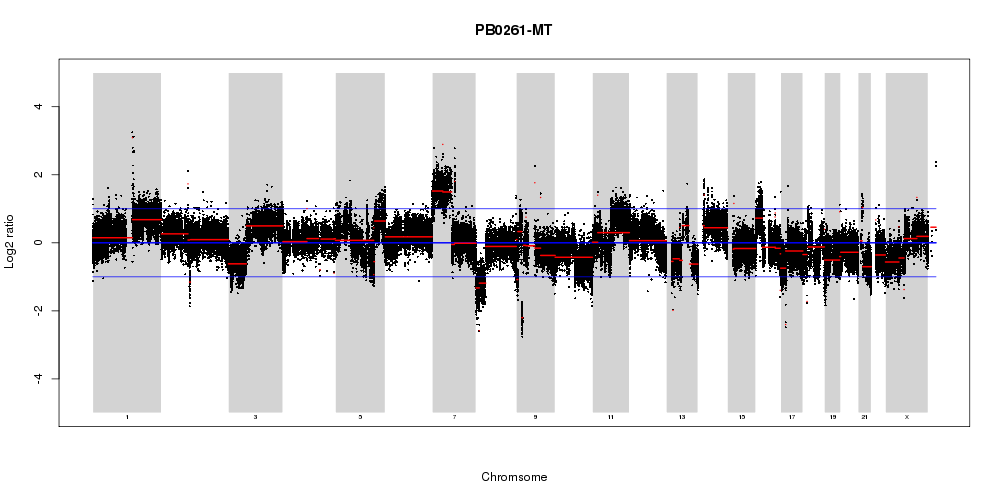
**

| **PB0261-P** | **PB0261-MT** |
| --- | --- |

**
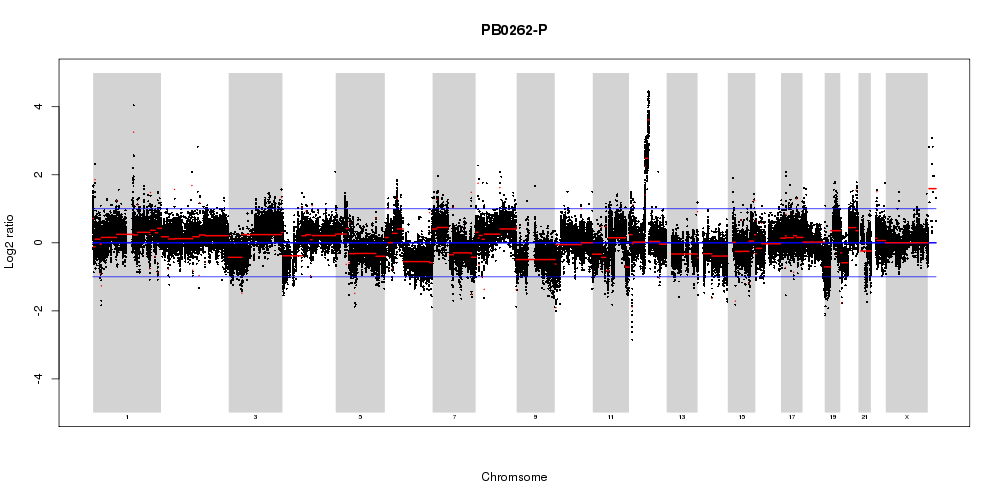

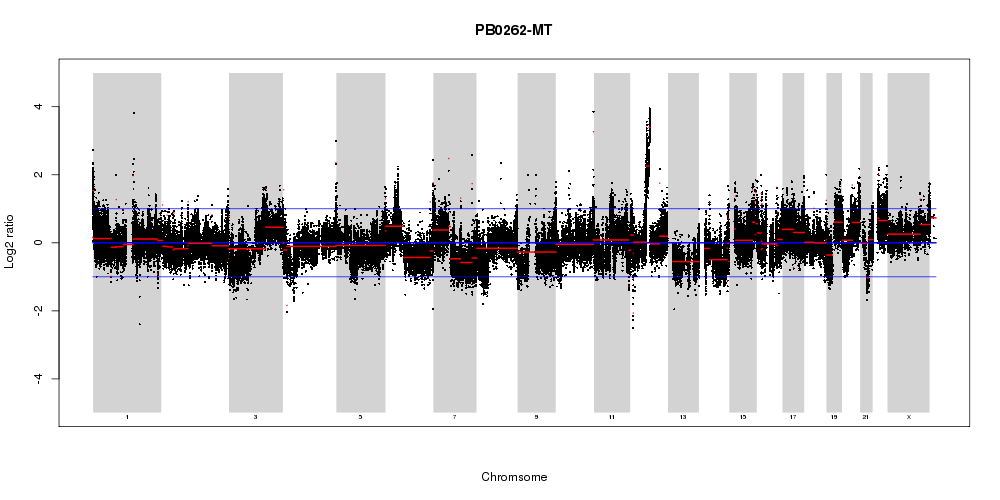
**

| **PB0262-P** | **PB0262-MT** |
| --- | --- |

**
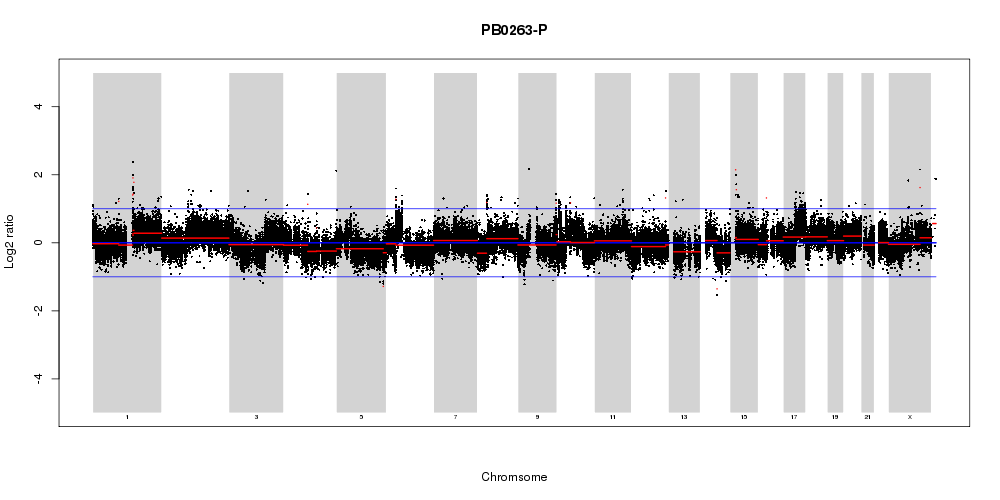

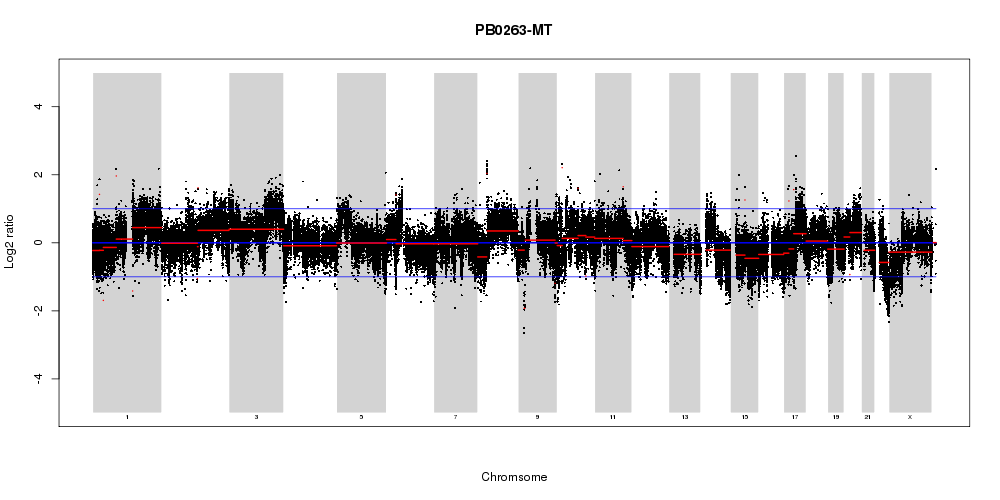
**

| **PB0263-P** | **PB0263-MT** |
| --- | --- |

**
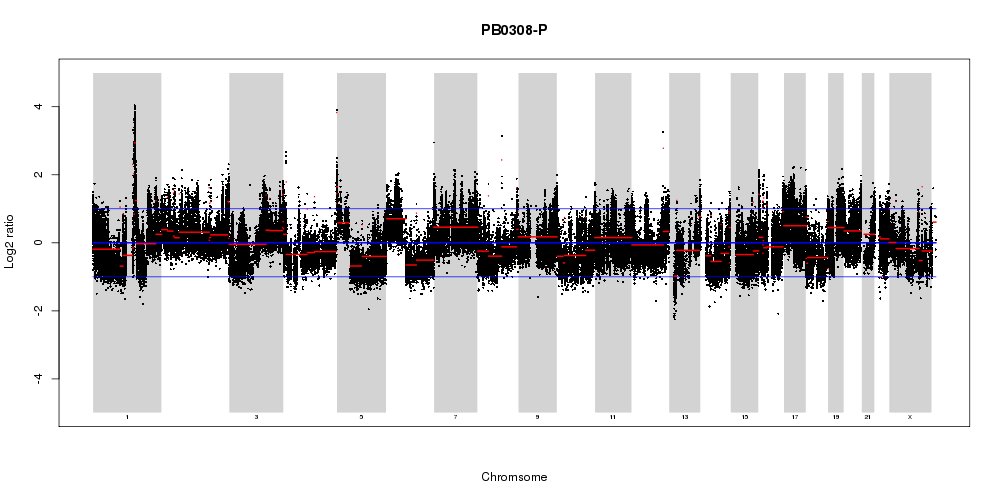

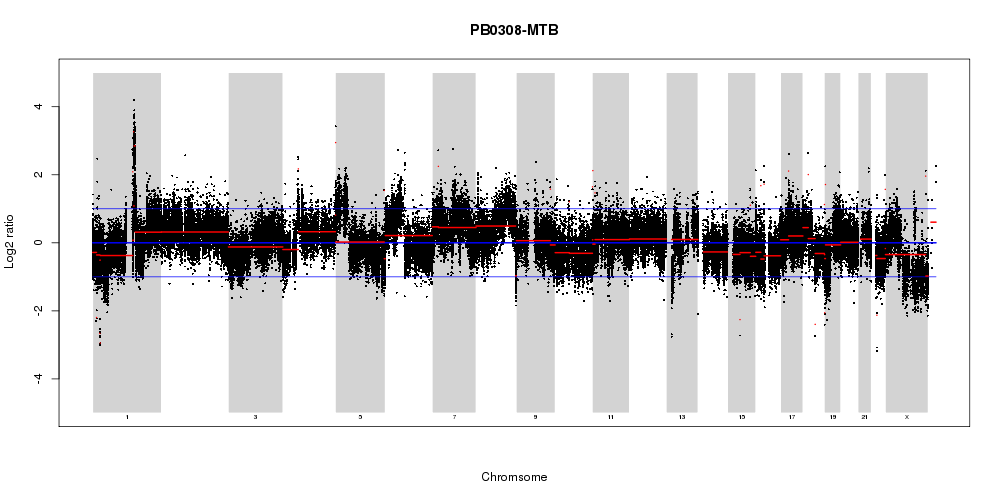
**

| **PB0308-P** | **PB0308-MTB** |
| --- | --- |

**
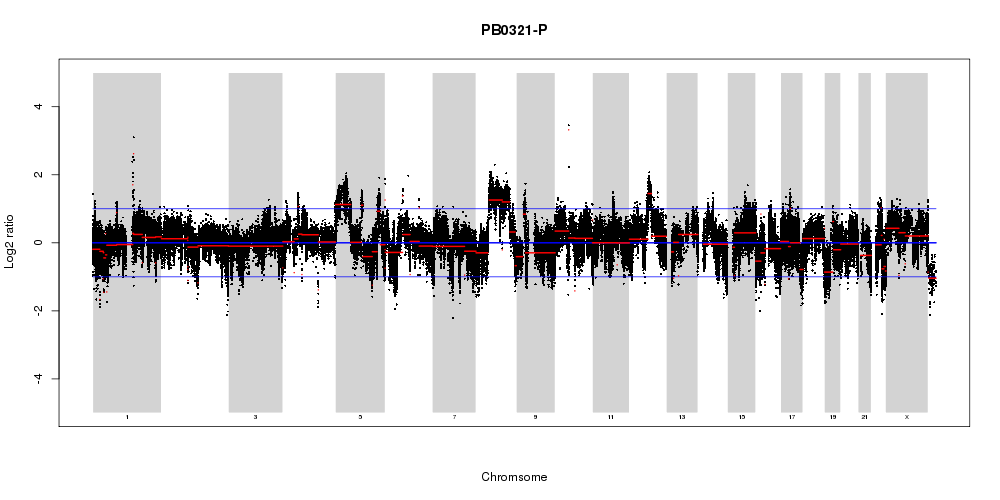

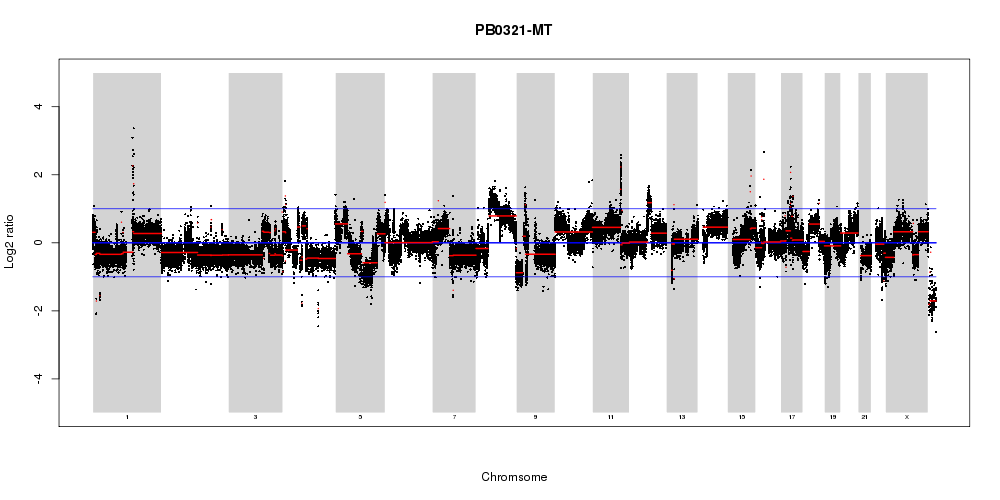
**

| **PB0321-P** | **PB0321-MT** |
| --- | --- |

**
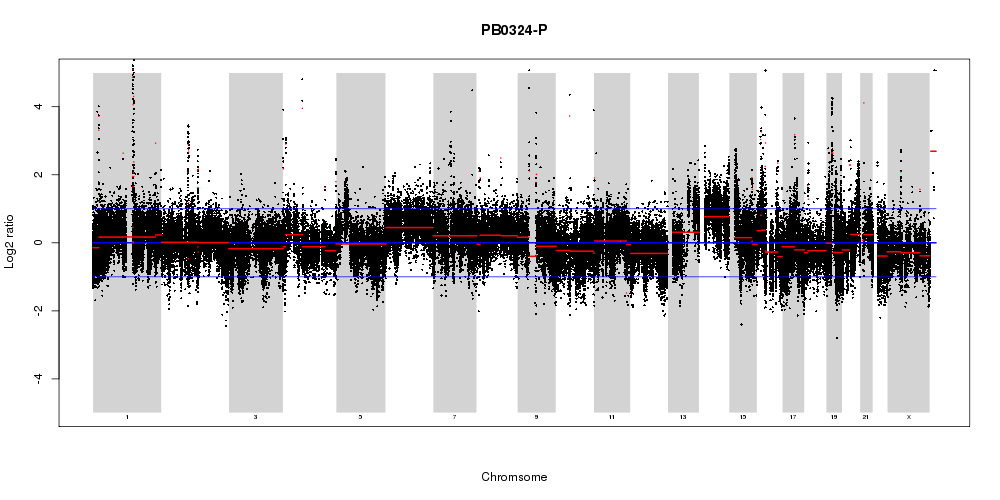

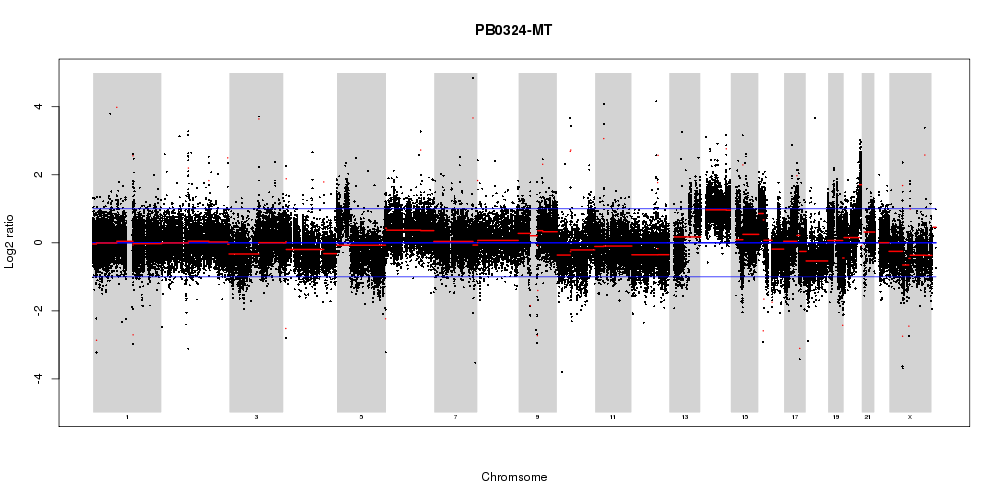
**

| **PB0324-P** | **PB0324-MT** |
| --- | --- |

**
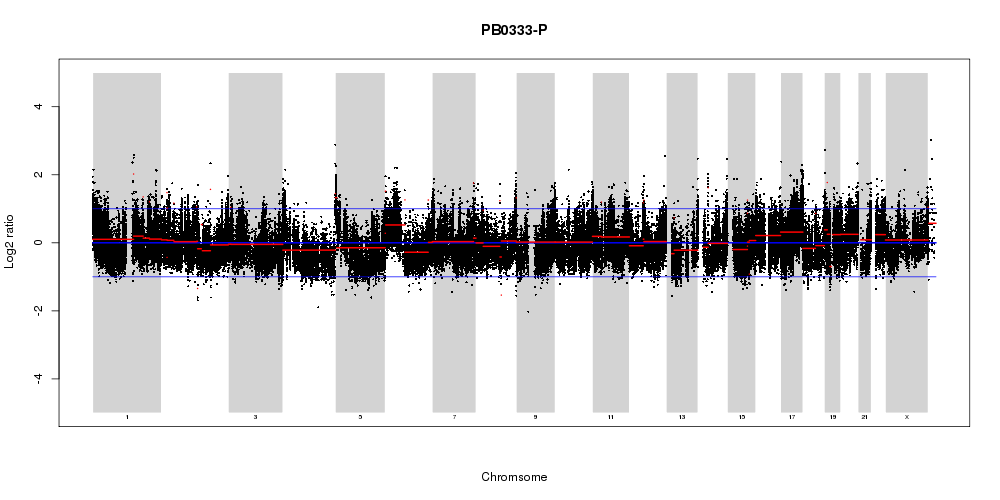

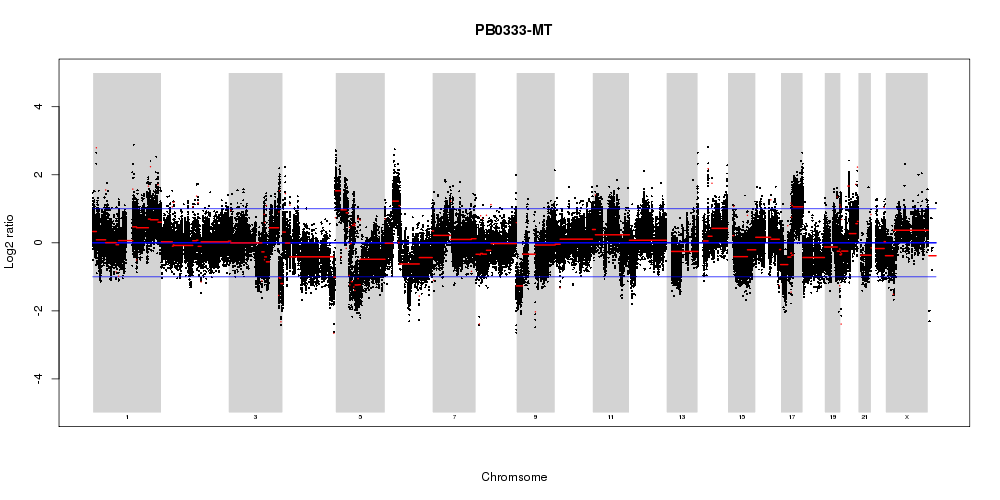
**

| **PB0333-P** | **PB0333-MT** |
| --- | --- |

**
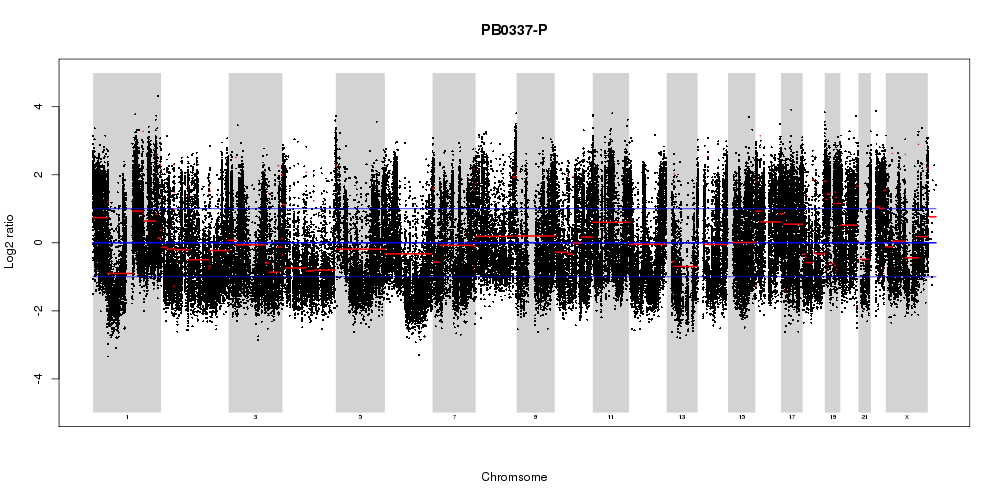

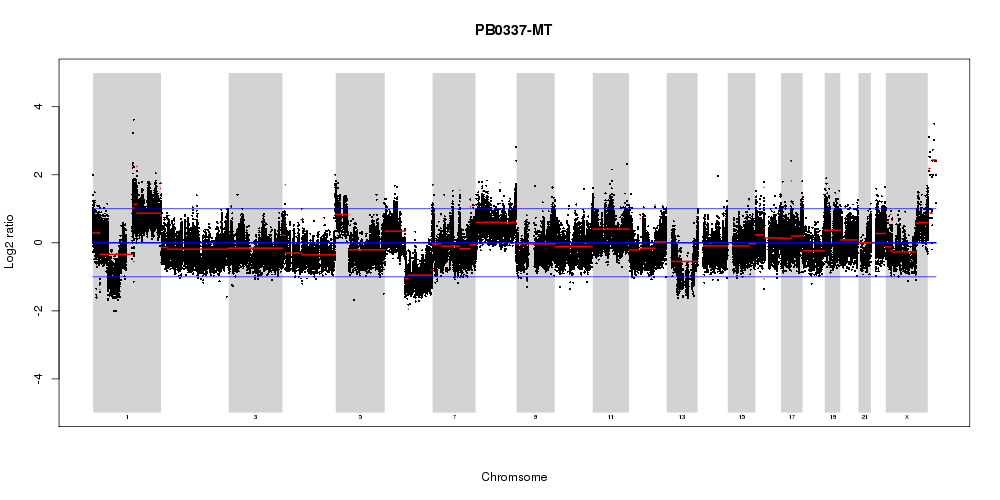
**

| **PB0337-P** | **PB0337-MT** |
| --- | --- |

**
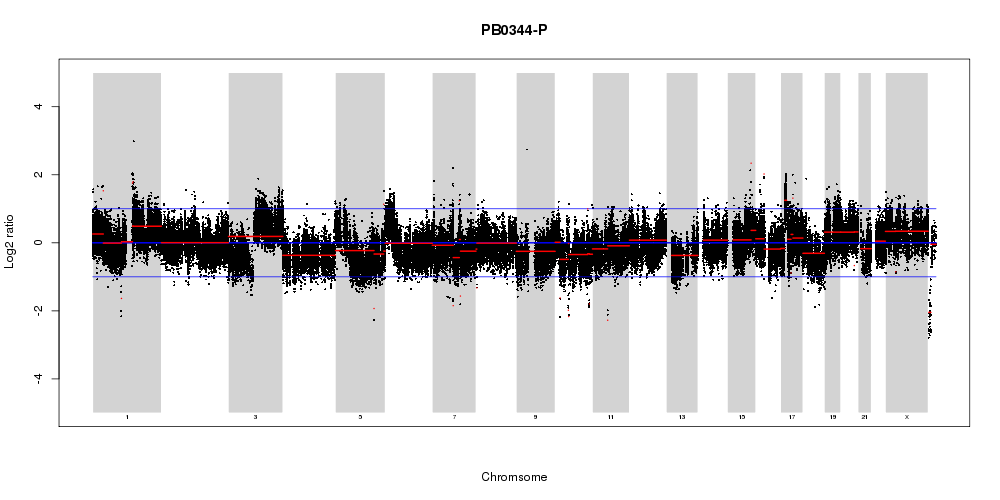

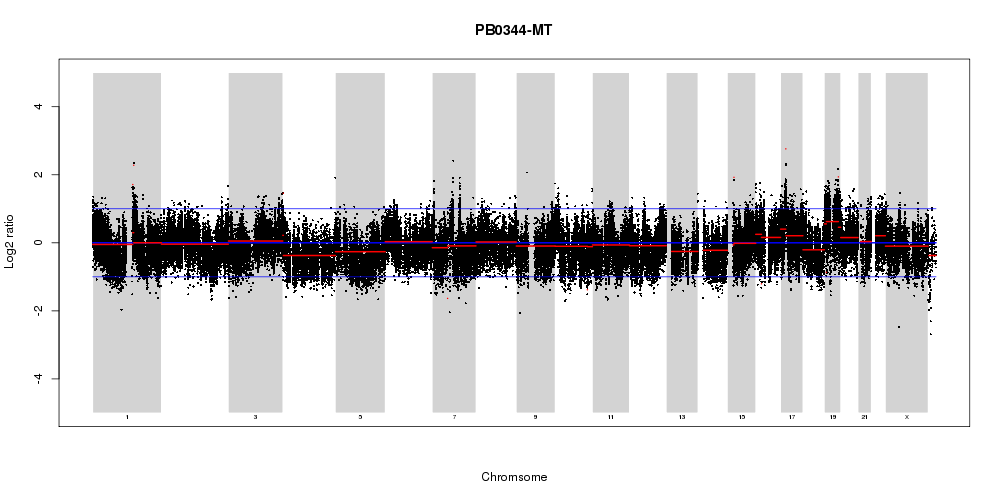
**

| **PB0344-P** | **PB0344-MT** |
| --- | --- |

**
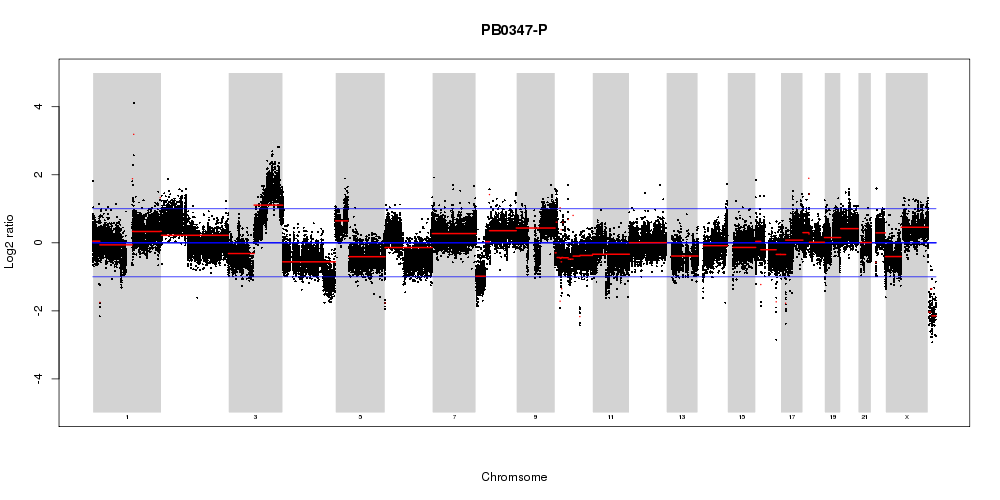

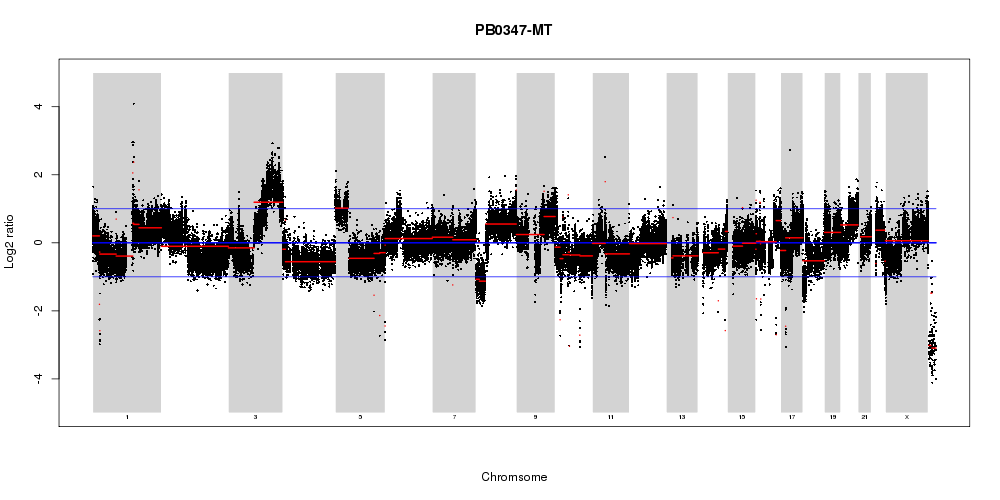
**

| **PB0347-P** | **PB0347-MT** |
| --- | --- |

**
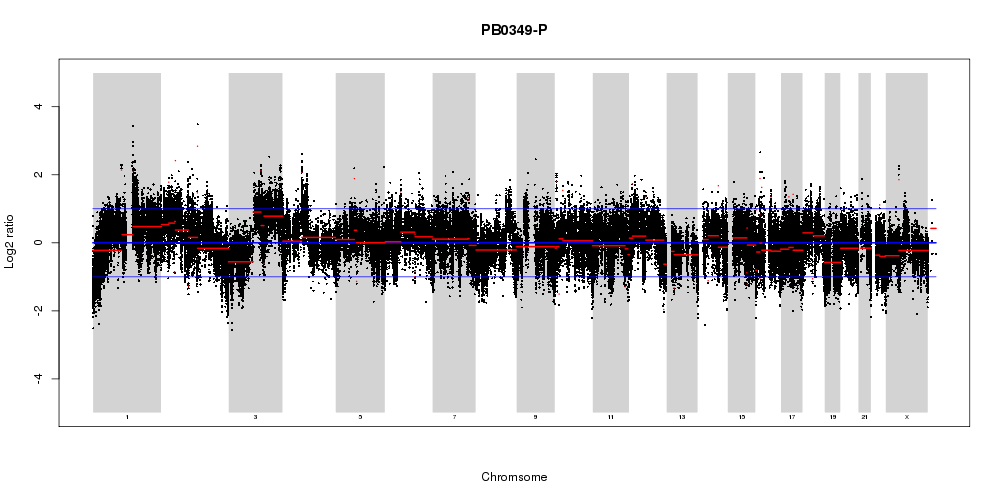

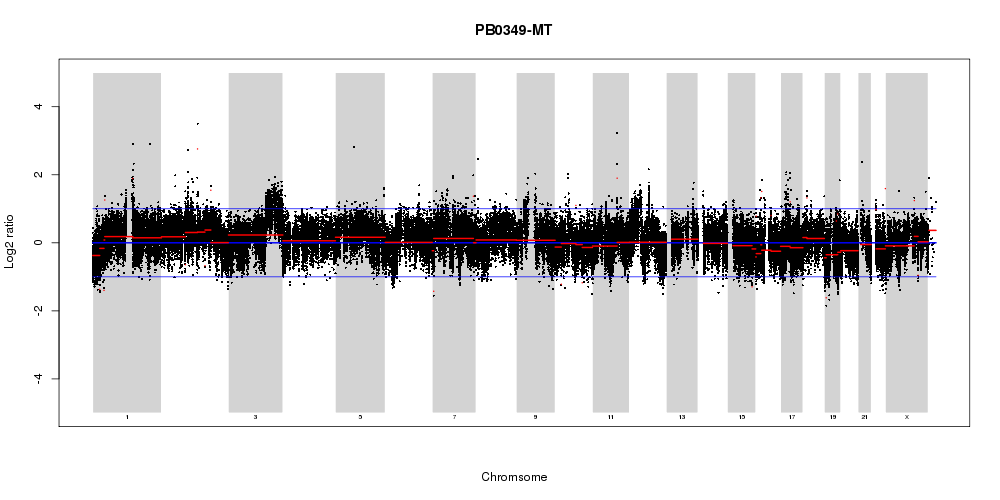
**

| **PB0349-P** | **PB0349-MT** |
| --- | --- |

**
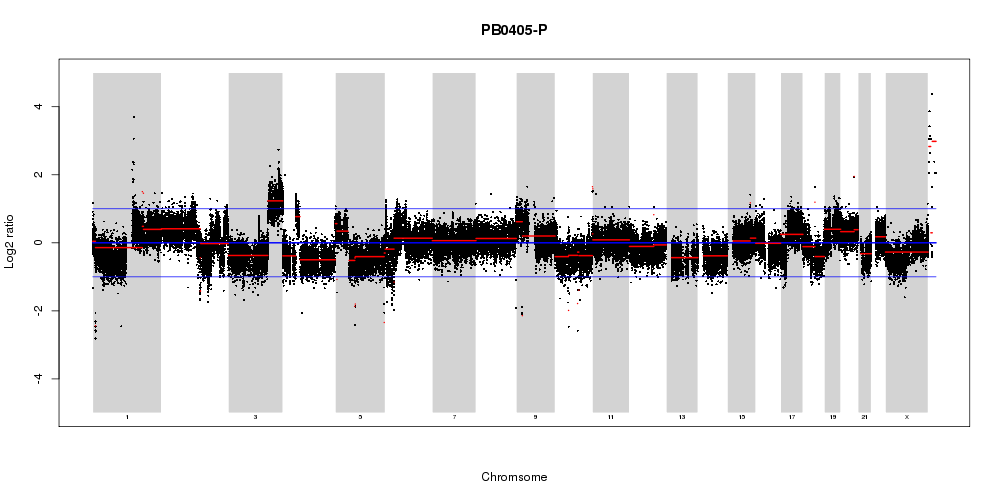

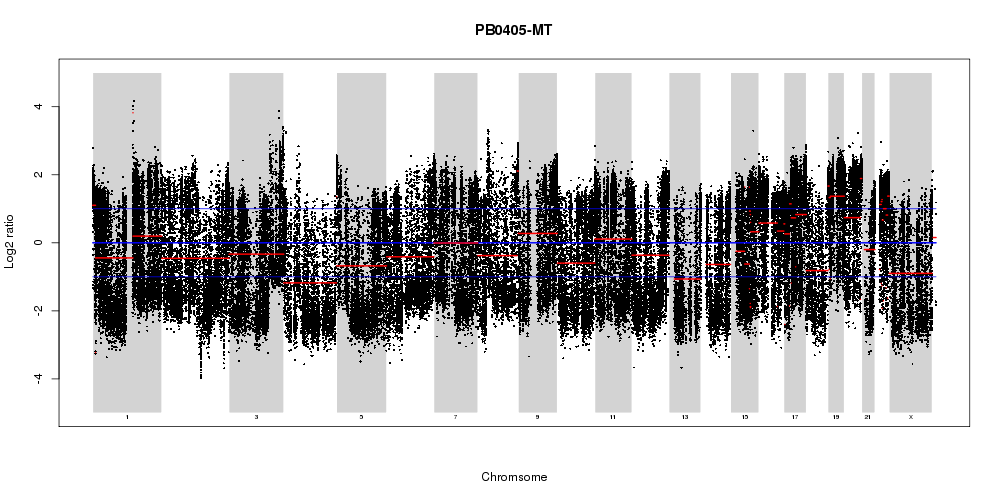
**

| **PB0405-P** | **PB0405-MT** |
| --- | --- |

**
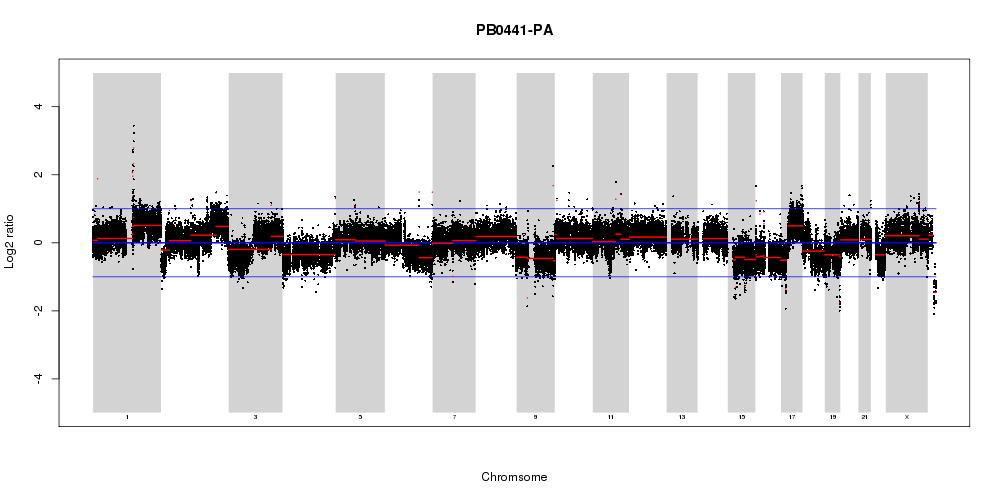

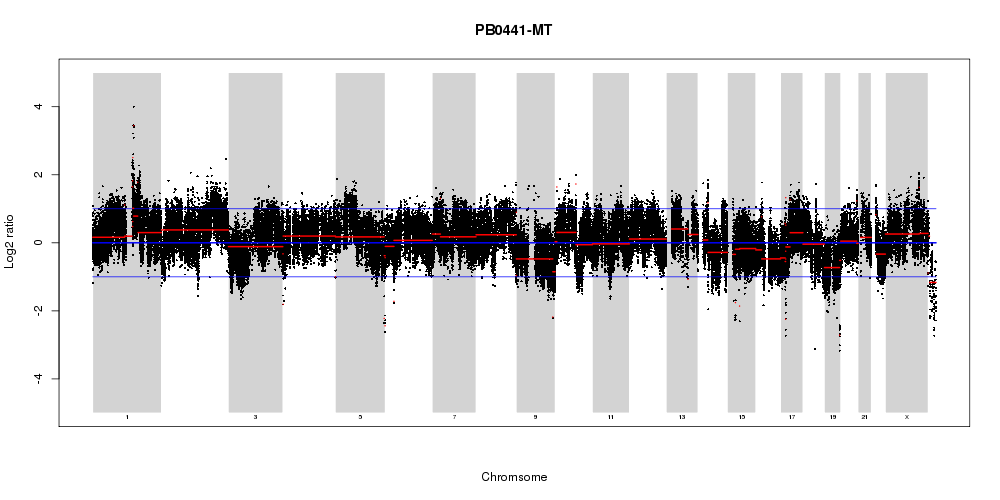
**

| **PB0441-PA** | **PB0405-MT** |
| --- | --- |

**Fig. S10. The raw log2 ratio plots for somatic copy number aberrations**


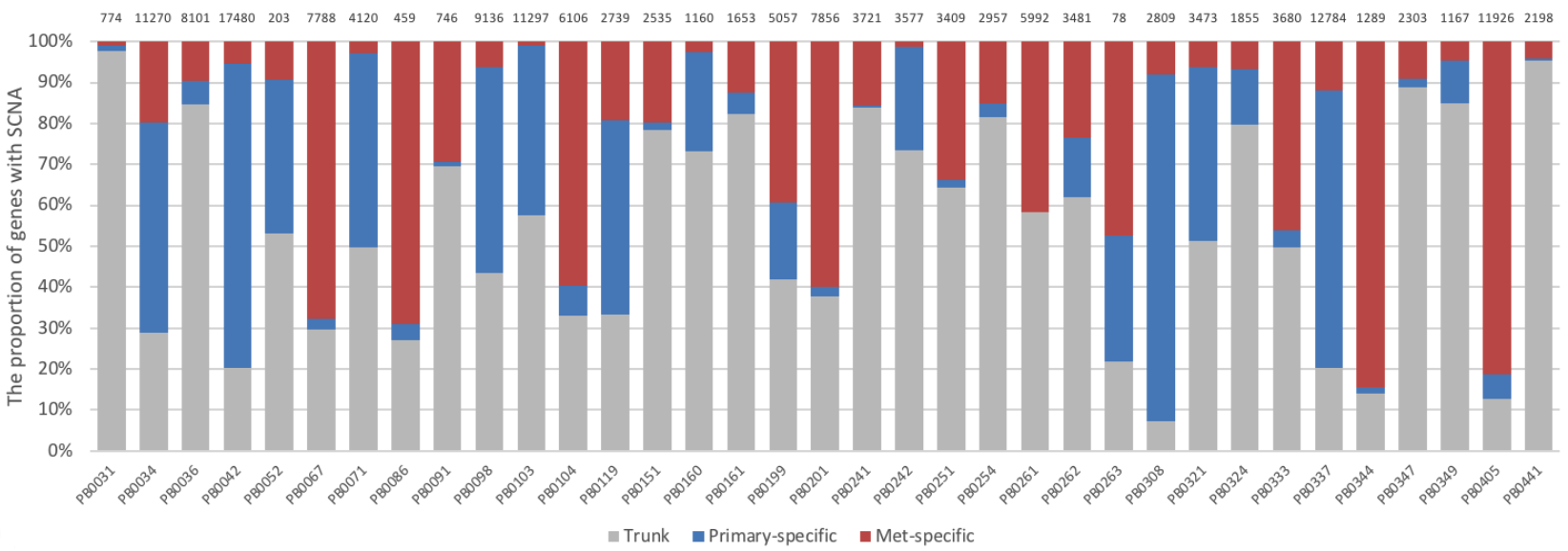


**Fig. S11. Concordance of somatic copy number aberrations (SCNAs) between primary tumors and paired metastases**

The proportion of shared (trunk, grey), primary-specific (blue) and metastasis-specific (red) SCNA events is shown for each of the 35 pairs of primary tumors and brain metastases (*Brastianos* *et el.*, *Cancer Discovery*, *2015*). SCNA events were defined at gene level. Specifically, segment log2 ratio means were assigned to genes within each segment with SCNA, so each sample would have log2 ratio values of the same number of genes for fair comparison between samples.

**
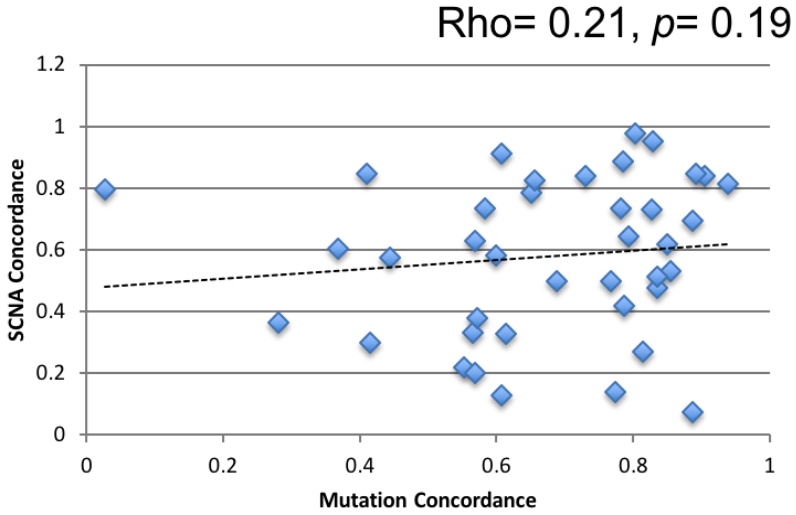
**

**Fig. S12. The correlation between SCNA concordance and mutation concordance**

The relationship between concordant ratio of SCNA profiles (primary tumors versus paired metastases) and concordant ratio of mutation profiles (primary tumors versus paired metastases) was assessed by the Spearman's rank-order correlation. The concordant ratio was defined as the number of shared events (SCNA events or somatic mutations) between primary tumors and paired metastases divided by the total number of events in the same primary tumor and metastasis pairs.


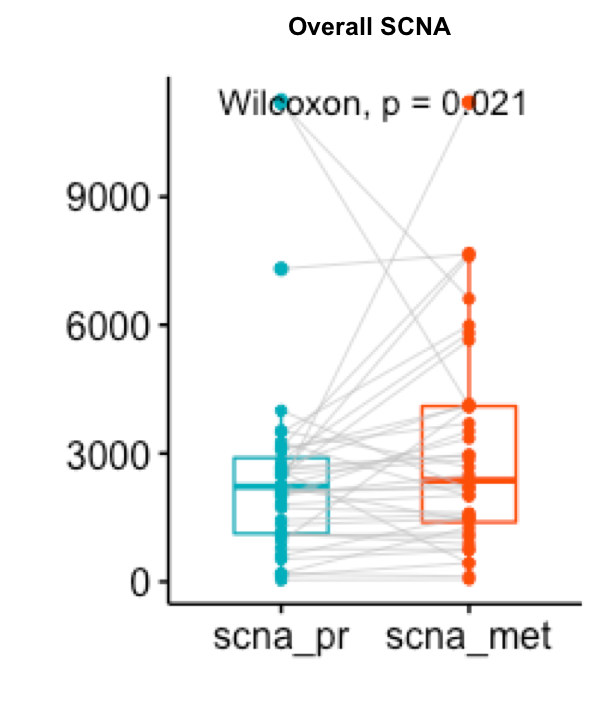


**Fig. S13. Overall SCNA burden for primary tumors and paired metastases**

Tumor purity-adjusted overall SCNA burden (copy number gains plus losses) was compared between primary tumors and paired metastases (40 pairs). SCNA events were defined at gene level. Specifically, segment log2ratio means were assigned to genes within each segment with SCNA so each sample would have log2ratio values of the same number of genes for fair comparison between samples.


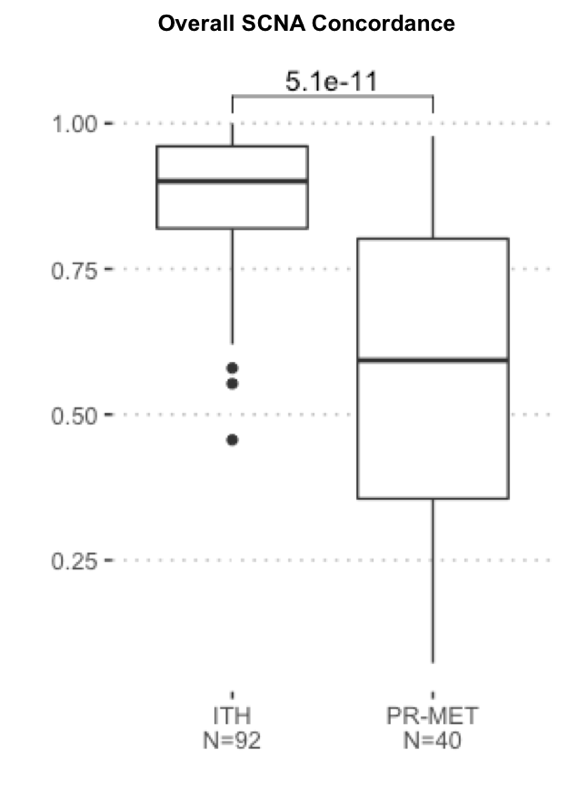


**Fig. S14. Overall SCNA concordance for ITH and primary-met pair (PR-MET) datasets**

Overall SCNA concordance between spatially separated tumor regions of the same tumors from TRACERx study (*Jamal-Hanjani M, et al., NEJM, 2017*) and 40 pairs of primary tumors and metastases in the current study. SCNA events were defined at gene level. Specifically, segment log2 ratio means were assigned to genes within each segment with SCNA so each sample would have log2 ratio values of the same number of genes for fair comparison between samples. The concordance was defined as the number of shared SCNA events between two tumor specimens divided by the total number of events in these two tumor specimen pairs.


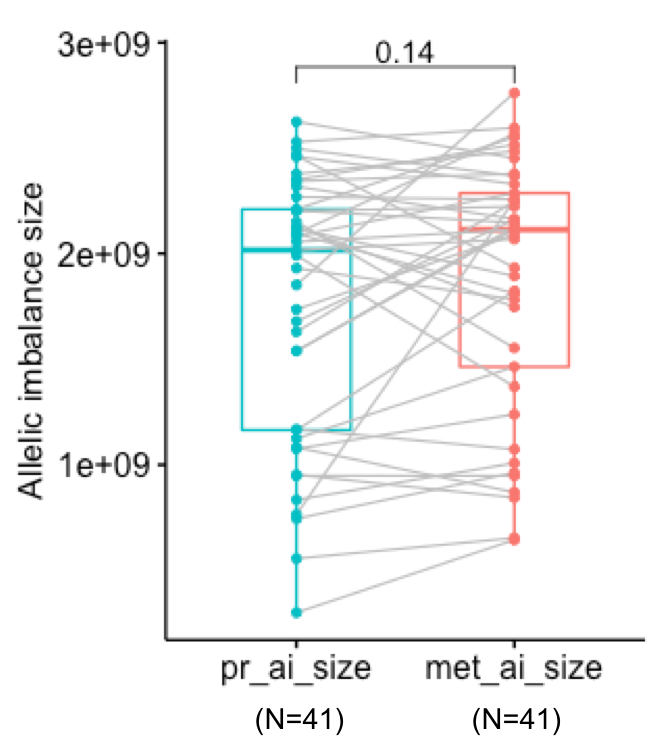


**Fig. S15. Comparison of allelic imbalance (AI) burden between primary tumors and paired metastases calculated by FACETS**


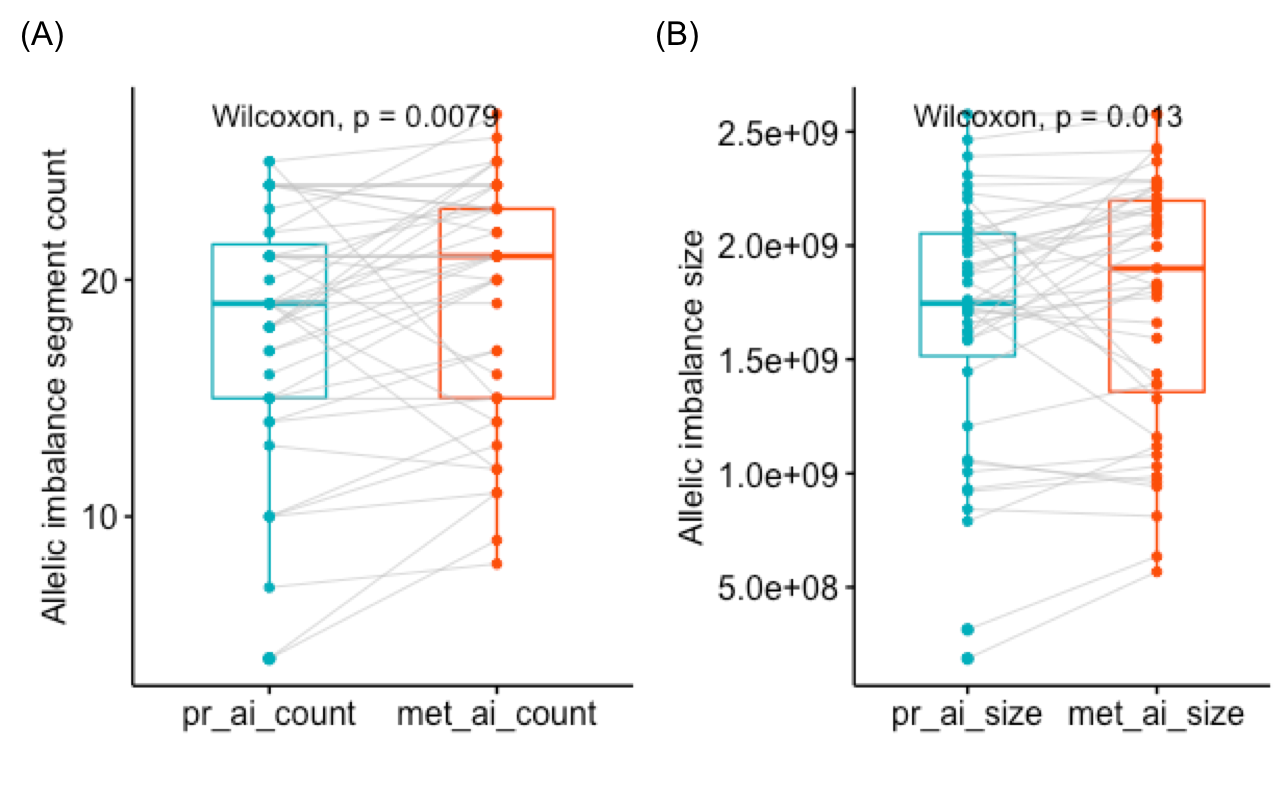


**Fig. S16. Comparison of allelic imbalance (AI) burden between primary tumors and paired metastases called by hapLOHseq**

**(A)** AI burden quantified as the number of genomic segments affected by AI. **(B)** AI burden quantified as the total size of genome affected by AI.

**Fig. S17. The level of allelic imbalance (AI) concordance for 35 pairs of primary tumors and brain metastases**

The proportion of shared (trunk, grey), primary-specific (blue) and metastasis-specific (red) AI events is shown for each of the 35 pairs of primary tumors and brain metastases (*Brastianos et el., Cancer Discovery, 2015*). The numbers on the top of the bars show the size of genomic regions (mega bases) subject to AI.

**Fig. S18. Immune score comparison between primary tumors and paired metastases**

**(A)** Pairwise immune score comparison between primary tumors and metastases. **(B)** Immune score difference (immune score for primary tumor minus immune score for metastases for each patient).

**Fig. S19. CD4 / CD8 T cell ratio in primary tumor (P) versus paired metastasis (M)**

**A B C D**

**E F G H**

**Fig. S20.** **Immune cell infiltration inferred from deconvolution of transcriptomic data using CIBERSORT in primary tumors versus metastases**

The y axis represents the proportion of each immune cell type in the specimen. The immune cell subset was inferred from gene expression profiling data using CIBERSORT. The difference was assessed by paired-sample Wilcoxon test. Immune cell subsets inferred with extremely low infiltration are not shown.

**Fig. S21. Immune scores for two external datasets with extracranial metastases**

Immune scores were calculated for the samples from 4 treatment naïve NSCLC patients (left, *Suda K, et al., J Thorac Oncol, 2018*) and 4 mice of genetic mouse model (GEMM) of human lung adenocarcinoma (right, *Gibbons DL, et al., PLos One, 2009*). Metastases with decreased immune scores compared to their primary tumors are indicated by dotted lines.

**Fig. S22. Patients with HLA LOH in either primary tumor and/or metastases**

16 patients with HLA LOH in either primary tumor and/or metastases. HLA LOH status are shown as YES (HLA LOH present) and NO (HLA LOH absent).

**Fig. S23. Association of HLA LOH with SCNA**

The number of genes with overall SCNA (left), copy number losses (middle) and copy number gains (right). Samples were divided to FALSE (HLA LOH absent) and TRUE (HLA LOH present). The difference was assessed by Wilcoxon rank sum test.

**Fig. S24. Comparison of HLA gene expression between primary tumors and metastases.** The difference was assessed by Wilcoxon signed-rank test.
