# Supplementary material for: Multiomics profiling of primary lung cancers and distant metastases reveals immunosuppression as a common characteristic of tumor cells with metastatic plasticity
Source: Genome Biol. 2020 Nov 4;21:271. doi: 10.1186/s13059-020-02175-0 (PMC7640699; doi:10.1186/s13059-020-02175-0)
Supplement: Supplementary file 3 — Additional file 3: Review history. [file 13059_2020_2175_MOESM3_ESM.docx]

Review History

**First round of review**

**Reviewer 1**

**Are you able to assess all statistics in the manuscript, including the appropriateness of statistical tests used?** No, I do not feel adequately qualified to assess the statistics.

**Comments to author:**

The manuscript by Lee et al measures mutations, CNV, methylation, and expression levels in primary NSCLCs and metastases. They conclude that generally metastases are usually "monoclonal" and that metastases tend to have greater numbers of SCNVs. They also indicate that transcripts involved in immune-related pathways are lower in metastases compared to primary NSCLCs. Overall the findings are interesting.

Several comments:

1) Results, pg 5: "took place late in the molecular evolutionary course"----this sentence is hard to understand. By the trees, most mets diverge after about two thirds of the total mutations had accumulated. Not sure if this finding really describes "late" metastasis. Please explain a bit more.

2) Results, pg 5: also using VAF to calculate a genetic distance seems a bit problematic because VAF depends on tumor purity. So, the same tumor with a bit more normal cells could have a fairly large genetic distance because the VAF adjustment changes for every mutation. Is there a difference if the is VAF adjustment is not made?

3) Results; "monoclonal seeding". These results are interesting. Based on the trees in Fig 1, perhaps the term "monophyletic" may also be appropriate

4) Results, pg 8: was there a size threshold for the SCNA calling? How does one call a SCNA the "same" between samples (ie exact breakpoints or what criterion are used?)

5) Results, pg 11: The transcription analysis and pathway studies are interesting. However, it was noted that the metastases have fewer immune cells. What is of interest and should be defined better, is whether the decrease in transcripts in immune-related pathways reflects down-regulation in the tumor cells or just fewer immune cells. This issue should at least be discussed to clarify this issue (reduced immune cells versus reduced immune-related expression in the tumor cells).

6) Results, pg 12: HLA LOH is potentially a mechanism of immune evasion. Was there any evidence at the transcript level (this data should be available, ie see #5)

7) AI in the Abstract is not defined

**Reviewer 2**

**Are you able to assess all statistics in the manuscript, including the appropriateness of statistical tests used? Yes, and I have assessed the statistics in my report.**

**Comments to author:**

Lee et al performed exome and RNAseq analysis on paired NSCLC primaries and mets, to study mechanisms underlying metastasis.

First they found that 67% of mutations are shared between primaries and mets. They suggest that this indicates metastasis was a late event in these tumours. In comparison to data from TRACERx, they found that based on VAFs mets are more distant from their primary, than different sections of primaries are from each other.

Based on PyClone analysis, mutations shared by primary/met samples clustered in the clone with highest representation (CCF). Using the "pigeonhole" principle, it appears that only a single founder clone was common to primary and met, with other clones being private to them.

I found the mutational process analysis of primary and mets to be one of the most interesting aspects. A smoking related signature was the most prominent in both primary and met samples, nd was also present in some mutations unique to mets (though they could have been missed due to region selection in the primary). Two signatures of unknown origin were unique to primaries; whereas APOBEC processes were unique to mets. Overall these suggest different signatures dominating at different stages of the evolution of these tumors.

Similar to point mutations, copy number alterations (SCNAs) were largely shared by primary and mets. However patients whose primaries and mets were similar in SCNAs could be different in terms of their SNVs. This was also seen in allelic imbalances though generally mets had higher numbers of these, consistent with more chromosomal instability.

Only 16 "canonical" mutations (in known oncogenes or tumor suppressors) were present, but 13 of them were shared in primary and met from same patient again suggesting acquisition prior to metastasis.

The DNA methylation analysis is quite surprising to me. If I read figure 3 properly, in terms of methylation pattern the primary and mets from a patient are more similar to each other than are the lung samples between patients (or the brain mets between patients). I would have expected that strong tissue specific differences in methylation would have dominated, as they do in the normal samples. However, it's not clear how this analysis was done. The last line of the Figure 3 caption reads "SRAM genes known to be regulated by DNA methylation in NSCLC were only used.". Does that apply to *all* the analyses in this figure, or just panel C? Given the differences between primaries and mets in terms of SNVs and SCNAs, it is notable that there are no methylation differences between them.

Transcriptionally, there was potentially immune dysregulation and lower infiltration in mets vs primaries; though it's not defined what (M density/P density) means in Fig 4c? As the authors note, this is based on small sample sizes. They attempt to validate in other available data, but only focus on datasets with paired samples. It seems like this pattern should emerge in non-paired data.

Questions & Comments:

Is anything known about the "unknown etiology" signatures that are unique to primaries?

The last line of the Figure 3 caption reads "SRAM genes known to be regulated by DNA methylation in NSCLC were only used.". Does that apply to *all* the analyses in this figure, or just panel C? In general the methods for the methylation analysis could be better explained.

What does (M density/P density) mean in Fig 4c? Did the authors try a proper deconvolution method such as CIBERSORTx? That could be much more informative.

Do the observations regarding down regulation of immune processes in mets hold up in datasets such as MET500? If it is so pervasive, then it should not need paired samples to pull out this relationship given the much higher sample size.

The authors speculate, reasonably, that cells that can down-regulate the immune response might be more readily able to metastasize. However, this analysis doesn't really show that since they do not attribute these changes to a specific cell type rather than to the general immune environrment in the lung vs brain. The supporting evidence is based on one patient (7) and on a couple of small external datasets. Again, there is a lot of public data available that could be used to test this more rigorously.

Are the authors sure that Nei's distance is appropriate in this context? It assumes a constant (and slow) rate of mutation over time. I'm not convinced whether that applies to tumors, particularly when they metastasize. Can they cite support for using this measure in the context of tumor evolution?

Parts of Figure 1 are very fuzzy and hard to read.

In general the figures are not very compelling in terms of their appearance. Spending some time on this would improve the overall impression given by the paper. At the moment they just look thrown together in a rush.

**Point-by-point response to reviewers’ comments**

**Reviewer #1**

The manuscript by Lee et al measures mutations, CNV, methylation, and expression levels in primary NSCLCs and metastases. They conclude that generally metastases are usually "monoclonal" and that metastases tend to have greater numbers of SCNVs. They also indicate that transcripts involved in immune-related pathways are lower in metastases compared to primary NSCLCs. Overall the findings are interesting.

**Author’s response:** We thank the reviewer for their overall favorable comments.

1) Results, pg 5: "took place late in the molecular evolutionary course"----this sentence is hard to understand. By the trees, most mets diverge after about two thirds of the total mutations had accumulated. Not sure if this finding really describes "late" metastasis. Please explain a bit more.

**Author’s response:** We appreciate the reviewer’s comment. We intended to say that at the time when the metastasis occurred, the tumors have already acquired a majority of mutations (explaining the large proportion of shared mutations between primary tumors and metastases), therefore metastasis could be a late event in molecular time. We agree that “metastasis took place late in the molecular evolutionary course” could be confusing. We therefore revised the sentence as the following:

***Results:*** *“These data suggest that at the time the metastasis occurred, these tumors were already late in their course of molecular evolution when most somatic mutations have already occurred.” (line 122-124)*

2) Results, pg 5: also using VAF to calculate a genetic distance seems a bit problematic because VAF depends on tumor purity. So, the same tumor with a bit more normal cells could have a fairly large genetic distance because the VAF adjustment changes for every mutation. Is there a difference if the is VAF adjustment is not made?

**Author’s response:** We appreciate the reviewer’s critical comment. As the reviewer pointed out, VAF can be impacted by tumor purity and local copy number changes. Therefore, in the revised manuscript, we re-calculated the Nei’s genetic distance using cancer cell fractions (CCFs) derived from PyClone, which adjusts both tumor purity and copy number changes of all mutations as reported in previous studies (Ding et al., 2019; Lee et al., 2017). Additionally, we also used the mean CCF difference of all mutations as well as Jaccard distance to quantify the genetic distance. These analyses showed the same results as in our original analyses that the genetic distance between primary tumors and paired metastases was greater than that between different regions within the same tumors (**Reviewer Figure 1**).

**C**

**B**

**A**

**Reviewer Figure 1 (Supplementary Figure 2 in revised manuscript). Genetic distance of somatic mutations.**

The pairwise genetic distance was compared between spatially separated tumor regions of the same tumors using intra-tumor heterogeneity (ITH) dataset (Jamal-Hanjani M, et al., NEJM, 2017) versus the 42 pairs of primary tumors and distant metastases (PM) in the current study. The genetic distance was calculated based on the cancer cell fraction (CCF) of all mutations inferred by PyClone adjusted for purity and local copy number changes. (A) Nei’s genetic distance. (B) The mean absolute cancer cell fraction (CCF) difference. (C) Jaccard distance. P-value was calculated by Wilcoxon rank-sum test.

We thank the reviewer for this critical suggestion. We have therefore replaced **Figure S2** and revised the manuscript accordingly, which reads as the following:

***Results:*** *“To precisely quantify the difference in mutational landscape in the context of intratumor heterogeneity (ITH), we calculated the genetic distance between primary NSCLC tumors and metastases based on the cancer cell fraction (CCF) of the mutations derived from PyClone considering variant allele frequency (VAF), tumor purity and local copy number changes (Roth et al., 2014).” (line 126-129)*

***Methods: “Genetic distance calculation***

*Using cancer cell fractions (CCFs) of all mutations including single nucleotide polymorphisms and INDELs, we calculated genetic distance between any given two samples (primary tumor vs. paired metastasis or two different regions of the same tumors). We used three different definitions of genetic distance. First, Nei’s genetic distance (Ding et al., 2019; Lee et al., 2017) was calculated as follows.*

$$D=-ln\frac{\sum\left( x_{i}y_{i}+\left( 1-x_{i} \right)\left( 1-y_{i} \right) \right)}{\sqrt{\left( \sum{x_{i}}^{2}+\left( 1-x_{i} \right)^{2} \right)\left( \sum{y_{i}}^{2}+\left( 1-y_{i} \right)^{2} \right)}}$$

*Here, x_i_ and y_i_ denote the CCFs of the i-th mutation in sample x and sample y, respectively.*

*Next, we calculated the genetic distance by taking the mean of absolute CCF difference defined as below.*

$$D=\frac{\sum\left| x_{i}-y_{i} \right|}{n}$$

*In the formula, x_i_ and y_i_ denote the CCFs of the i-th mutation in sample x and sample y respectively, and n is the number of total mutations.*

*Finally, the Jaccard distance (Gates et al., 2019) was calculated as follows.*

$$D=\frac{\sum\left( x_{i}-y_{i} \right)^{2}}{\sum{x_{i}}^{2}+\sum{y_{i}}^{2}-\sum\left( x_{i}y_{i} \right)}$$

*In the Jaccard distance definition, x_i_ and y_i_ denote the CCFs of the i-th mutation in sample x and sample y, respectively.*

*For the ITH dataset, one tumor can have multiple two-sample combinations. In this case, we took the average distance among all the possible pairwise combinations.” (line 505-531)*

3) Results; "monoclonal seeding". These results are interesting. Based on the trees in Fig 1, perhaps the term "monophyletic" may also be appropriate

**Author’s response:** We thank the reviewer for this suggestion and agree that "monophyletic" is appropriate. We have included “monophyletic” seeding pattern in the revised manuscript which now read as the following:

***Results:*** *“Taken together, these data suggest monoclonal/monophyletic seeding may be a predominant mode of metastasis in the majority of NSCLCs.” (line 152-153)*

4) Results, pg 8: was there a size threshold for the SCNA calling? How does one call a SCNA the "same" between samples (ie exact breakpoints or what criterion are used?)

**Author’s response:** The reviewer raises a very important question regarding fair comparison of SCNA data between specimens. As the reviewer pointed out, it is difficult to call “the same SCNA”, particularly from exome sequencing data since only a small proportion of the genome was sampled leading to different breakpoints in different samples. Therefore, for SCNA analysis, we used a gene-based algorithm implemented in the CNTools R package, an approach widely used for SCNA analyses from exome sequencing data (Beird et al., 2018; Roh et al., 2017; Seth et al., 2019). Specifically, we assigned segment log2 ratio means to genes within those segments with SCNA so each sample would have log2 ratio values of the same number of genes for fair comparison between samples. We agree that the methodological details are important for readers to assess whether the analyses are appropriate. We therefore expanded the relevant section in Methods and briefly described it in the Results in the revised manuscript as follows:

***Results:*** *“We next delineated the genome-wide SCNA profiles of primary NSCLCs and paired distant metastases using a gene-based SCNA analysis algorithm for exome sequencing data that allows fair comparison of the SCNAs between different samples (Beird et al., 2018; Roh et al., 2017; Seth et al., 2019)* *to identify shared and unique SCNA events (Fig. S10).” (line 212-215)*

***Methods:*** *“We applied ExomeCN, a gene-based SCNA analysis algorithm for exome sequencing data that allows fair comparison of the SCNAs between different samples. Specifically, we first obtained copy number segments with copy ratios between tumor and normal. The log2 copy number ratios of the segments were then assigned to the genes within the segments by CNTools (Zhang, 2018) so each sample would have log2 ratio values of the same number of genes for fair comparison between samples.” (line 538-543)*

5) Results, pg 11: The transcription analysis and pathway studies are interesting. However, it was noted that the metastases have fewer immune cells. What is of interest and should be defined better, is whether the decrease in transcripts in immune-related pathways reflects down-regulation in the tumor cells or just fewer immune cells. This issue should at least be discussed to clarify this issue (reduced immune cells versus reduced immune-related expression in the tumor cells).

**Author’s response:** The reviewer makes an important point. We believe reduced immune cell infiltration is the major reason for down-regulation of immune-related pathways in metastases as evidenced by the immunohistochemical (IHC) data (**Reviewer Figure 2**) and deconvolution of gene expression data (**Reviewer Figures 3-5)**.

As shown in **Reviewer Figure 2**, the IHC data demonstrated that infiltration of immune cells was lower in metastases than matched primary lung cancers except in *Patient 7*.

**Reviewer Figure 2 (Figure 4C in revised manuscript).** **Comparison of immune cell infiltration between primary NSCLC tumors and paired metastases by immunohistochemistry (IHC) of immune markers (CD3, CD4, CD8, CD20 and PD1).** The density was defined as the number of cells positive for each marker per mm2. The y axis shows the ratio (log2) of density of each cell type in metastases versus that in paired primary lung cancers.

To further address this important question, we calculated the ESTIMATE (Yoshihara et al., 2013) score, an estimate of infiltration of overall immune cells, which demonstrated significant lower ESTIMATE scores in metastases than primary tumors suggesting decreased immune infiltration in metastases (**Reviewer Figure 3**).

**Reviewer Figure 3 (Figure 5A in revised manuscript).** **The overall immune cell infiltration was inferred by RNA-seq data using ESTIMATE.** The y axis represents the ESTIMATE score in the specimen. The difference was assessed by paired-sample Wilcoxon test.

Additionally, we deconvoluted gene expression data by CIBERSORT (**Reviewer Figure 4**) and TIMER (**Reviewer Figure 5**) to confirm these findings and evaluate immune cell subsets at a more granular level. The findings also revealed overall lower immune cell infiltration in metastases compared to primary tumors.

**Reviewer Figure 4 (Supplementary Figure 20 in revised manuscript). Immune cell infiltration inferred from deconvolution of transcriptomic data using CIBERSORT in primary tumors versus metastases.** The y axis represents the proportion of each immune cell type in the specimen. The difference was assessed by paired-sample Wilcoxon test. Immune cell subsets inferred with extremely low infiltration are not shown.

**Reviewer Figure 5 (Figure 5B-G in revised manuscript).** **The immune cell subsets were inferred by deconvolution of RNA-seq data using TIMER.** The y axis represents the proportion of each immune cell type in the specimen. The difference was assessed by paired-sample Wilcoxon test.

Considering immune-related genes are generally most strongly expressed in the immune cells themselves, we believe these results support that the down-regulation of immune-related pathways in metastases was mainly related to reduced immune cell infiltration in metastases. We thank the reviewer again for this insightful comment and we included these new analyses and discussed this important question in revised manuscript, now read as the following:

***Results:*** *“Moreover, metastases showed significantly lower immune score (Roh et al., 2017) than primary tumors (Fig. S18, p=0.042). These patterns were accompanied by lower CD3, CD4, CD8, CD20 and PD-1 densities in metastases than primary tumors in 4 of 5 patients by IHC (Fig. 4C). Beyond the overall decreased infiltration of immune cells in metastases, a higher CD4/CD8 T cell ratio was seen, potentially supportive of the immunosuppressive environment predominating in metastases (Fig. S19).*

*To further understand down-regulation of immune-related pathways in metastases, we deconvoluted the transcriptomic data using various algorithms. We first applied ESTIMATE, an algorithm estimating the infiltration of overall immune cells (Yoshihara et al., 2013), and the analysis revealed significantly lower ESTIMATE scores in metastases than primary tumors (Fig. 5A) suggesting an overall lower immune cell infiltration in metastases. Next, we de-convoluted gene expression data by TIMER (Li et al., 2016) to infer the infiltration of main immune cell types. As shown in Fig. 5B-G, the infiltration of most immune cell types was higher in primary lung cancers than metastases with the exception of macrophages, which were higher in metastases (Fig. 5D) and CD4+ T cells that were similar between primary and metastases (Fig. 5F). We further applied CIBERSORT to classify the immune subsets at a more granular level (Newman et al., 2015). Unfortunately, the inferred infiltration of most immune cell subsets derived by CIBERSORT was very low in this cohort of tumors (Fig. S20) precluding comparison between primary tumors and metastases. Nevertheless, CIBERSORT data suggested the macrophages were predominately pro-tumor M2 macrophages (Gordon, 2003), which were significantly higher in metastases (Fig. S20H). Taken together, these results suggest that down-regulation of immune-related pathways was mainly due to reduced immune cell infiltration in metastases.” (line 321-344)*

6) Results, pg 12: HLA LOH is potentially a mechanism of immune evasion. Was there any evidence at the transcript level (this data should be available, ie see #5)

**Author’s response:** This is a great question. We found that 16 of 43 patients with exome sequencing data harbored HLA LOH in either primary NSCLC and/or metastases and 5 of them harbored HLA LOH only in metastases suggesting HLA LOH-positive cancer cells may have the advantage to evade immune surveillance and establish distant metastases. It is reasonable to speculate that other potential mechanisms may decrease HLA expression leading to ineffective neoantigen presentation and immune evasion. As the reviewer suggested, we compared the expression of HLA genes between the 8 pairs of primary NSCLCs and matched distant metastases. As shown in **Reviewer Figure 6**, the expression of HLA genes was overall lower in metastases, but the difference did not reach statistical significance.

**Reviewer Figure 6 (Supplementary Figure 24 in revised manuscript). Comparison of HLA gene expression between primary tumors and metastases.** The difference was assessed by Wilcoxon signed-rank test.

Although immune evasion may be a required feature for cancer cells with metastatic capacity, the molecular mechanisms leading to immune evasion are likely heterogeneous and inactivation of HLA by LOH or at the transcript level may be one of the many possible mechanisms. Further studies with larger cohorts of paired primaries-metastases are needed to further assess the possibility of decreased HLA expression as a potential mechanism of immune evasion. We discussed this valuable point in the revised manuscript, which now reads as the following:

***Results:*** *“…We further compared the expression of HLA genes between the 8 pairs of primary NSCLCs and matched distant metastases to explore whether decreased HLA expression may lead to ineffective neoantigen presentation and immune evasion. As shown in Fig. S24, the expression of HLA genes was overall lower in metastases, but the difference did not reach statistical significance.” (line 372-376)*

***Discussion:*** *“… Given the heterogeneous nature of NSCLC, the molecular mechanisms leading to immune evasion are likely heterogeneous across different tumors. Further studies with larger cohorts of primary-metastasis are needed to identify particular molecular features associated with immunosuppression in metastases.” (line 450-453)*

7) AI in the Abstract is not defined

**Author’s response:** We apologize for this omission and have now defined AI in the Abstract.

**Reviewer #2**

Lee et al performed exome and RNAseq analysis on paired NSCLC primaries and mets, to study mechanisms underlying metastasis.

First they found that 67% of mutations are shared between primaries and mets. They suggest that this indicates metastasis was a late event in these tumours. In comparison to data from TRACERx, they found that based on VAFs mets are more distant from their primary, than different sections of primaries are from each other.

Based on PyClone analysis, mutations shared by primary/met samples clustered in the clone with highest representation (CCF). Using the "pigeonhole" principle, it appears that only a single founder clone was common to primary and met, with other clones being private to them.

I found the mutational process analysis of primary and mets to be one of the most interesting aspects.  A smoking related signature was the most prominent in both primary and met samples, and was also present in some mutations unique to mets (though they could have been missed due to region selection in the primary). Two signatures of unknown origin were unique to primaries; whereas APOBEC processes were unique to mets. Overall these suggest different signatures dominating at different stages of the evolution of these tumors.

Similar to point mutations, copy number alterations (SCNAs) were largely shared by primary and mets. However patients whose primaries and mets were similar in SCNAs could be different in terms of their SNVs. This was also seen in allelic imbalances though generally mets had higher numbers of these, consistent with more chromosomal instability.

Only 16 "canonical" mutations (in known oncogenes or tumor suppressors) were present, but 13 of them were shared in primary and met from same patient again suggesting acquisition prior to metastasis.

The DNA methylation analysis is quite surprising to me. If I read figure 3 properly, in terms of methylation pattern the primary and mets from a patient are more similar to each other than are the lung samples between patients (or the brain mets between patients). I would have expected that strong tissue specific differences in methylation would have dominated, as they do in the normal samples. However, it's not clear how this analysis was done. The last line of the Figure 3 caption reads "SRAM genes known to be regulated by DNA methylation in NSCLC were only used.". Does that apply to *all* the analyses in this figure, or just panel C? Given the differences between primaries and mets in terms of SNVs and SCNAs, it is notable that there are no methylation differences between them.

Transcriptionally, there was potentially immune dysregulation and lower infiltration in mets vs primaries; though it's not defined what (M density/P density) means in Fig 4c? As the authors note, this is based on small sample sizes. They attempt to validate in other available data, but only focus on datasets with paired samples. It seems like this pattern should emerge in non-paired data.

**Author’s response:** We thank the reviewer for these favorable comments and constructive critiques, which are addressed point-by-point below.

**Questions & Comments:**

1. Is anything known about the "unknown etiology" signatures that are unique to primaries?

**Author’s response:** We thank the reviewer for this important question. In our study, we used widely referenced COSMIC mutational signatures to define the mutation processes. The COSMIC mutational signatures are based on the analysis of whole exome and whole-genome data across various cancer types. When a mutation signature is present in a small number of cancers that cannot give sufficient evidence pointing to certain etiology or present in multiple cancer types, it is often classified as “etiology unknown”. In our datasets, two mutational signatures, Signatures 5 and 30, were classified as “etiology unknown”, both of which were associated with primary-only mutations. Signature 5 was associated with smoking in lung cancers, but it was also found in all cancer types and most cancer samples, many of which are not associated with smoking. It was therefore defined as “etiology unknown” (Alexandrov et al., 2016; Alexandrov et al., 2013). On the other hand, Signature 30 has been observed in a small subset of breast cancers giving insufficient evidence defining its etiology (Alexandrov et al., 2016; Alexandrov et al., 2013). However, it has been reported that human stem cells with base excision repair protein NTHL1 deleted by CRISPR-Cas9 showed a predominant increase in Signature 30-associated mutations (Drost et al., 2017). We added additional information about the two signatures in the revised manuscript which reads as the following:

***Results:*** *“On the other hand, Signature 5 (etiology-unknown, associated with smoking in lung cancers (Alexandrov et al., 2013)) and Signature 30 (etiology-unknown, associated with base excision repair in model system (Drost et al., 2017)) emerged as the top mutational signatures for primary-only mutations…” (line 187-190)*

2. The last line of the Figure 3 caption reads "SRAM genes known to be regulated by DNA methylation in NSCLC were only used.". Does that apply to *all* the analyses in this figure, or just panel C? In general the methods for the methylation analysis could be better explained.

**Author’s response:** We apologize for the lack of clarity. We compared DNA methylation profiles between primary NSCLCs and paired distant metastases with 3 different approaches: (1) We performed unsupervised clustering based on the DNA methylation levels for all CpG islands (n=27,000), which revealed the general DNA methylation landscape was similar between primary tumors and metastases (**Fig. 3A**); (2) In **Fig. 3B**, we focused on the promoter DNA methylation level of individual genes that were regulated by DNA methylation (negative correlation between DNA methylation and gene expression) based on the data from our main cohort of samples including normal samples (n=24) and showed no significant difference between primary tumors and metastases; and (3) In **Fig. 3C**, we compared the methylation status of primary NSCLCs to their paired metastases focusing on the previously reported 521genes known to be regulated by DNA methylation in NSCLC (Significantly Repressed in Association with DNA Methylation (SRAM)) and again identified no significant difference. Taken together, these data suggested that the methylation landscape was overall similar between primary NSCLC and paired metastases.

We totally understand that these results were surprising to the reviewer as strong tissue-specific methylation patterns are expected. These results were unexpected to us too. One possible explanation is that the tumor purity was overall high in this cohort of specimens. Therefore, the difference was primarily driven by the cancer cells where inter-patient heterogeneity dominated. We agree with the reviewer that these details are important to interpret the data in context, and therefore have revised the figure legend and methods accordingly, which now read as the following:

***Methods: “DNA methylation analysis***

*Methylation profiling was done using Infinium MethylationEPIC Kit (Illumina, Inc.) according to manufacturer’s protocol. Data normalization and beta value determination was carried out using Illumina GenomeStudio Software with the additional Methylation Module Software. RnBeads package in R was then used to derive 27,000 CpG island methylation values. The hierarchical clustering based on average DNA methylation level of the CpG islands was performed with the correlation distance metric (1 – Pearson correlation coefficient). We identified a list of genes whose promoter DNA methylation was negatively correlated with mRNA expression (Spearman’s rank correlation coefficient <= -0.5) by correlating DNA methylation and gene expression data of all samples including normal tissues in our cohort. We also obtained previously reported 521 Significantly Repressed in Association with DNA Methylation (SRAM) genes known to be regulated by DNA methylation in NSCLC from the study examining 73 cell lines (Lin et al., 2014) and compared their promoter DNA levels between primary tumors and paired metastases in our cohort.” (line 579-592)*

***Figure Legend: “Figure 3. DNA methylation similarity between primary tumors and paired metastases.*** *(A) Unsupervised clustering of all samples including normal tissues based on DNA methylation level of all CpG islands (n=27,000). (B) Correlation of promoter DNA methylation between primary tumors and metastases for the 1,084 genes showing a negative correlation between DNA methylation and gene expression (Spearman’s rank correlation <= -0.5 based on the data in our main cohort). (C) Correlation of promoter DNA methylation level between primary tumors and metastases for the previously reported 521 genes to be regulated by DNA methylation in NSCLC from the study examining 73 cell lines.” (line 688-695)*

3. What does (M density/P density) mean in Fig 4c? Did the authors try a proper deconvolution method such as CIBERSORT? That could be much more informative.

**Author’s response:** We apologize for the lack of clarity in **Fig 4C**. “M density” referred to the cell density (counts/mm^2^) in the metastases while “P density” referred to the same in the primary tumors by immunohistochemistry (CD3, CD4, CD8, CD20 and PD1). We have revised the figure legend which now reads:

***Figure Legend:*** *“Fig 4C.* *Comparison of immune cell infiltration between primary NSCLC tumors and paired metastases by immunohistochemistry (IHC) of immune markers (CD3, CD4, CD8, CD20 and PD1). The density was defined as the number of cells positive for each marker per mm2. The y axis shows the ratio (log2) of density of each cell type in metastases versus that in paired primary lung cancers.” (line 705-709)*

Furthermore, as suggested by the reviewer, we used deconvolution approaches to infer immune infiltration in these tumors. We first applied the CIBERSORT, which revealed lower proportions of activated dendritic cells and a higher proportion of M2 macrophages in metastases (**Reviewer Fig. 7**). However, the infiltration of most immune cell subsets was very low in this cohort of tumors (close to 0, data not shown here) precluding comparison between primary lung cancers and metastases. We therefore performed immune cell deconvolution using TIMER (**Reviewer Fig. 8**) (Li et al., 2016) and ESTIMATE (an estimate of infiltration of overall immune cells, **Reviewer Fig. 9**). In line with the pathway analysis, the infiltration of overall immune infiltration inferred by ESTIMATE and most immune cell types inferred by TIMER was higher in primary lung cancers than metastases with the exception of CD4+ T cells that were similar between primary and metastases and macrophages, which were higher in metastases (**Reviewer Fig. 8** and **9**). Interestingly, the data from CIBERSORT suggested that these macrophages are predominantly pro-tumor M2 macrophages (Gordon, 2003) (**Reviewer Fig. 7**).

**Reviewer Figure 7 (Supplementary Figure 20 in revised manuscript). Immune cell infiltration inferred from deconvolution of transcriptomic data using CIBERSORT in primary tumors versus metastases.** The y axis represents the proportion of each immune cell type in the specimen. The difference was assessed by paired-sample Wilcoxon test. Immune cell subsets inferred with extremely low infiltration are not shown.

**Reviewer Figure 8 (Figure 5B-G in revised manuscript).** **The immune cell subsets were inferred by deconvolution of RNA-seq data using TIMER.** The y axis represents the proportion of each immune cell type in the specimen. The difference was assessed by paired-sample Wilcoxon test.

**Reviewer Figure 9 (Figure 5A in revised manuscript).** **The overall immune cell infiltration was inferred by RNA-seq data using ESTIMATE.** The y axis represents the ESTIMATE score in the specimen. The difference was assessed by paired-sample Wilcoxon test.

We thank the reviewer for this suggestion and believe these analyses have substantially strengthened our story. Therefore, we have included these analyses in the revised manuscript and discussed these findings in results, which now reads as the following:

***Results:*** *“To further understand down-regulation of immune-related pathways in metastases, we deconvoluted the transcriptomic data using various algorithms. We first applied ESTIMATE, an algorithm estimating the infiltration of overall immune cells (Yoshihara et al., 2013), and the analysis revealed significantly lower ESTIMATE scores in metastases than primary tumors (Fig. 5A) suggesting an overall lower immune cell infiltration in metastases. Next, we de-convoluted gene expression data by TIMER (Li et al., 2016) to infer the infiltration of main immune cell types. As shown in Fig. 5B-G, the infiltration of most immune cell types was higher in primary lung cancers than metastases with the exception of macrophages, which were higher in metastases (Fig. 5D) and CD4+ T cells that were similar between primary and metastases (Fig. 5F). We further applied CIBERSORT to classify the immune subsets at a more granular level (Newman et al., 2015). Unfortunately, the inferred infiltration of most immune cell subsets derived by CIBERSORT was very low in this cohort of tumors (Fig. S20) precluding comparison between primary tumors and metastases. Nevertheless, CIBERSORT data suggested the macrophages were predominately pro-tumor M2 macrophages (Gordon, 2003), which were significantly higher in metastases (Fig. S20H). Taken together, these results suggest that down-regulation of immune-related pathways was mainly due to reduced immune cell infiltration in metastases.” (line 329-344)*

4. Do the observations regarding down regulation of immune processes in mets hold up in datasets such as MET500? If it is so pervasive, then it should not need paired samples to pull out this relationship given the much higher sample size.

**Author’s response:** We thank the reviewer for this constructive suggestion and agree paired samples shouldn’t be required if using a much larger cohort. Unfortunately, there were very little data from lung cancer metastases even for unpaired specimens. Although MET500 was a big cohort, there were actually only 24 patients with lung cancers and only 5 specimens were distant metastases (n=3 for liver; n=1 for adrenal; n=1 for brain). The remaining 19 specimens included 12 lymph node metastases that are known to have unique immune infiltration and immune microenvironment (Fransen, Arens, & Melief, 2013; Munn & Mellor, 2006; Shu, Cochran, Huang, Morton, & Maecker, 2006) and 7 arguably local spread (lung, hilum, pleural and chest wall) rather than distant metastases **(Reviewer Table 1)**. Nevertheless, we attempted to download the data to perform the suggested analyses. However, access to these data requires a Material Transfer Agreement and additional paperwork which is being significantly delayed due to COVID-19. Our legal department has informed us it will take at least 3-4 months to get the approval to run these analyses. Since there were only 5 unpaired distant metastases, we respectfully request they do not be required in this study.

**Reviewer Table 1. Number of met samples with lung as a primary site**

| **Primary Site** | **Biopsy Site** | **Patient ID** |
| --- | --- | --- |
| Lung | Lymph Node | MO_1108, MO_1111, MO_1125, MO_1150, MO_1189, MO_1236, MO_1250, MO_1286, MO_1294, MO_1397, MO_1433, MO_1558 |
| Lung | Lung | MO_1034, MO_1315, TP_2042 |
| Lung | Liver | MO_1163, MO_1206, MO_1325 |
| Lung | Brain | MO_1119 |
| Lung | Pleura | MO_1177 |
| Lung | Chest Wall | MO_1194 |
| Lung | Lung Nodule | MO_1209 |
| Lung | Hilum | MO_1253 |
| Lung | Adrenal | TP_2052 |

We additionally explored the Human Cancer Metastasis Database (HCMDB), an integrated database specifically designed to store and analyze large scale expression data of cancer metastasis from Gene Expression Omnibus (GEO) and The Cancer Genome Atlas (TCGA) (Zheng et al., 2018). Unfortunately, our search for metastatic lung cancer resulted in only 4 studies (**Reviewer Table 2**) and only one (EXP0073) had microarray-based gene expression data of lymph node metastases (without primary tumors) from 5 lung cancer patients.

**Reviewer Table 2. Experiment results with metastatic lung cancer in HCMDB**

| **Exp ID** | **GEO Accession** | **Cancer Type** | **Metastasis Sites** | **Design** | **Data availability** | **Patient #** |
| --- | --- | --- | --- | --- | --- | --- |
| EXP00038 | GSE18549 | lung cancer | brain  lymph node | Comparing tumors with brain vs tumors with LN metastasis | primary tumors only | 9 |
| EXP00045,  EXP00046 | GSE1987 | lung cancer | NA | Comparing primary tumors and normal lungs | primary tumors only | 25 |
| EXP00073 | GSE29827 | lung cancer | lymph node | Comparing metastases from different subtypes of primary lung cancer | Lymph node metastasis only | 5 |

Furthermore, we reached out to various investigators to identify unpublished data which could be used to validate our findings. We were fortunate to find 3 independent datasets with gene expression data (from microarray) available: PROSPECT (275 primary NSCLC, previously published), BATTLE-1 (95 metastatic NSCLCs, unpublished) and BATTLE-2 (144 metastatic NSCLCs, unpublished). However, quality control analyses demonstrated extremely severe batch effect from unsupervised clustering as shown in **Reviewer Figure 10**. We have discussed with several biostatistics and bioinformatics experts, all of whom advised against using these datasets for comparison. The consensus concern was that with such marked batch effect, the results would not be reliable after batch effect correction is applied.

**Reviewer Figure 10. Profound batch effect between 3 microarray gene expression datasets.** PROSPECT: microarray data from 275 primary NSCLCs. BATTLE-1: microarray data from 95 patients with metastatic NSCLCs. BATTLE-2: microarray data from 144 patients with metastatic NSCLCs.

We apologize for not being able to expand these analyses to larger cohorts, though we believe this reflects the limited molecular profiling data available for lung cancer metastases, and supports the value of our study. This highlights the need for more multi-omics studies exploring lung cancer metastases to underscore the molecular mechanisms of metastases and design more effective treatments in the adjuvant and metastatic settings. We thank the reviewer again for this suggestion and have discussed this important limitation of our study and the need for more profiling studies on lung cancer metastases in the revised manuscript which now reads as the following:

*“One important limitation of the current study is the small sample size, particularly for transcriptomic profiling analyses. Unfortunately, there are very little molecular profiling data available from lung cancer distant metastases even for unpaired specimens. For example, MET500 is a comprehensive molecular profiling study on 500 adult patients with metastatic solid tumors, thus far the largest dataset on molecular profiling of metastases (Robinson et al., 2017). However, there were only 24 lung cancer patients in the cohort and only 5 specimens with distant metastases (n=3 for liver; n=1 for adrenal; n=1 for brain). This highlights the need for more multi-omics studies on lung cancer metastases to underscore the molecular mechanisms underlying distant metastases of NSCLC.” (line 455-463)*

5. The authors speculate, reasonably, that cells that can down-regulate the immune response might be more readily able to metastasize. However, this analysis doesn't really show that since they do not attribute these changes to a specific cell type rather than to the general immune environment in the lung vs brain. The supporting evidence is based on one patient (7) and on a couple of small external datasets. Again, there is a lot of public data available that could be used to test this more rigorously.

**Author’s response:** We thank the reviewer’s again for this suggestion. As stated in the response above, there were no appropriate available large datasets to adequately address this question. We discussed this important limitation of our study and the need for more profiling studies on lung cancer metastases in the revised manuscript as mentioned above.

6. Are the authors sure that Nei's distance is appropriate in this context? It assumes a constant (and slow) rate of mutation over time. I'm not convinced whether that applies to tumors, particularly when they metastasize. Can they cite support for using this measure in the context of tumor evolution?

**Author’s response:** We appreciate the reviewer’s critical comment. Nei’s genetic distance is one of commonly used approaches to quantify genomic heterogeneity in cancer evolution studies (Ding et al., 2019; Lee et al., 2017). Nevertheless, we agree with the reviewer regarding the limitations of Nei’s distance. Therefore, in the revised manuscript, we applied two alternative approaches to quantify the genetic distance: the mean cancer cell fractions (CCFs, derived from PyClone, which adjusts both tumor purity and copy number changes) of all mutations and Jaccard distance (Gates et al., 2019). These analyses showed the same results as in our original analyses that the genetic distance between primary tumors and paired metastases was greater than that between different regions within the same tumors (**Reviewer Figure 11**).

**C**

**B**

**A**

**Reviewer Figure 11 (Supplementary Figure 2 in revised manuscript). Genetic distance of somatic mutations.** The pairwise genetic distance was compared between spatially separated tumor regions of the same tumors using intra-tumor heterogeneity (ITH) dataset (Jamal-Hanjani M, et al., NEJM, 2017) versus the 42 pairs of primary tumors and distant metastases (PM) in the current study. The genetic distance was calculated based on of CCF of all mutations inferred by PyClone adjusted for purity and local copy number changes using **(A)** Nei’s genetic distance, **(B)** the mean absolute cancer cell fraction (CCF) difference and **(C)** Jaccard distance. P-value was calculated by Wilcoxon rank-sum test.

We appreciate for this critical suggestion. We have therefore replaced **Figure S2** and revised the manuscript accordingly, which reads as the following:

***Results:*** *“To precisely quantify the difference in mutational landscape in the context of intratumor heterogeneity (ITH), we calculated the genetic distance between primary NSCLC tumors and metastases based on the cancer cell fraction (CCF) of the mutations considering variant allele frequencies (VAF), tumor purity and local copy number changes (Roth et al., 2014).” (line 126-129)*

***Methods: “Genetic distance calculation***

*Using cancer cell fractions (CCFs) of all mutations including single nucleotide polymorphisms and indels, we calculated genetic distance between any given two samples (primary tumor vs. paired metastasis or two different regions of the same tumors). We used three different definitions of genetic distance. First, Nei’s genetic distance (Ding et al., 2019; Lee et al., 2017)* *was calculated as follows.*

$$D=-ln\frac{\sum\left( x_{i}y_{i}+\left( 1-x_{i} \right)\left( 1-y_{i} \right) \right)}{\sqrt{\left( \sum{x_{i}}^{2}+\left( 1-x_{i} \right)^{2} \right)\left( \sum{y_{i}}^{2}+\left( 1-y_{i} \right)^{2} \right)}}$$

*Here, x_i_ and y_i_ denote the CCFs of the i-th mutation in sample x and sample y, respectively.*

*Next, we calculated the genetic distance by taking the mean of absolute CCF difference defined as below.*

$$D=\frac{\sum\left| x_{i}-y_{i} \right|}{n}$$

*In the formula, x_i_ and y_i_ denote the CCFs of the i-th mutation in sample x and sample y respectively, and n is the number of total mutations.*

*Finally, the Jaccard distance (Gates et al., 2019) was calculated as follows.*

$$D=\frac{\sum\left( x_{i}-y_{i} \right)^{2}}{\sum{x_{i}}^{2}+\sum{y_{i}}^{2}-\sum\left( x_{i}y_{i} \right)}$$

*In the Jaccard distance definition, x_i_ and y_i_ denote the CCFs of the i-th mutation in sample x and sample y, respectively.*

*For the ITH dataset, one tumor can have multiple two-sample combinations. In this case, we took the average distance among all the possible pairwise combinations.” (line 505-531)*

7. Parts of Figure 1 are very fuzzy and hard to read.

**Author’s response:** We apologize for the resolution of **Figure 1** and have remade it and all remaining figures to address this concern.

8. In general the figures are not very compelling in terms of their appearance. Spending some time on this would improve the overall impression given by the paper. At the moment they just look thrown together in a rush.

**Author’s response:** We apologize for the appearance conveyed by our figures. We have now remade all figures to improve esthetics and readability and hope they are now satisfactory.

**References**

Alexandrov, L. B., Ju, Y. S., Haase, K., Van Loo, P., Martincorena, I., Nik-Zainal, S., . . . Stratton, M. R. (2016). Mutational signatures associated with tobacco smoking in human cancer. *Science, 354*(6312), 618-622. doi:10.1126/science.aag0299

Alexandrov, L. B., Nik-Zainal, S., Wedge, D. C., Aparicio, S. A., Behjati, S., Biankin, A. V., . . . Stratton, M. R. (2013). Signatures of mutational processes in human cancer. *Nature, 500*(7463), 415-421. doi:10.1038/nature12477

Beird, H. C., Wu, C. C., Ingram, D. R., Wang, W. L., Alimohamed, A., Gumbs, C., . . . Somaiah, N. (2018). Genomic profiling of dedifferentiated liposarcoma compared to matched well-differentiated liposarcoma reveals higher genomic complexity and a common origin. *Cold Spring Harb Mol Case Stud, 4*(2). doi:10.1101/mcs.a002386

Ding, X., He, M., Chan, A. W. H., Song, Q. X., Sze, S. C., Chen, H., . . . Wong, N. (2019). Genomic and Epigenomic Features of Primary and Recurrent Hepatocellular Carcinomas. *Gastroenterology, 157*(6), 1630-1645 e1636. doi:10.1053/j.gastro.2019.09.005

Drost, J., van Boxtel, R., Blokzijl, F., Mizutani, T., Sasaki, N., Sasselli, V., . . . Clevers, H. (2017). Use of CRISPR-modified human stem cell organoids to study the origin of mutational signatures in cancer. *Science, 358*(6360), 234-238. doi:10.1126/science.aao3130

Fransen, M. F., Arens, R., & Melief, C. J. (2013). Local targets for immune therapy to cancer: tumor draining lymph nodes and tumor microenvironment. *Int J Cancer, 132*(9), 1971-1976. doi:10.1002/ijc.27755

Gates, E. D. H., Yang, J., Fukumura, K., Lin, J. S., Weinberg, J. S., Prabhu, S. S., . . . Schellingerhout, D. (2019). Spatial Distance Correlates With Genetic Distance in Diffuse Glioma. *Front Oncol, 9*, 676. doi:10.3389/fonc.2019.00676

Gordon, S. (2003). Alternative activation of macrophages. *Nat Rev Immunol, 3*(1), 23-35. doi:10.1038/nri978

Lee, J. K., Wang, J., Sa, J. K., Ladewig, E., Lee, H. O., Lee, I. H., . . . Nam, D. H. (2017). Spatiotemporal genomic architecture informs precision oncology in glioblastoma. *Nat Genet, 49*(4), 594-599. doi:10.1038/ng.3806

Li, B., Severson, E., Pignon, J. C., Zhao, H., Li, T., Novak, J., . . . Liu, X. S. (2016). Comprehensive analyses of tumor immunity: implications for cancer immunotherapy. *Genome Biol, 17*(1), 174. doi:10.1186/s13059-016-1028-7

Lin, S. H., Wang, J., Saintigny, P., Wu, C. C., Giri, U., Zhang, J., . . . Heymach, J. V. (2014). Genes suppressed by DNA methylation in non-small cell lung cancer reveal the epigenetics of epithelial-mesenchymal transition. *BMC Genomics, 15*, 1079. doi:10.1186/1471-2164-15-1079

Munn, D. H., & Mellor, A. L. (2006). The tumor-draining lymph node as an immune-privileged site. *Immunol Rev, 213*, 146-158. doi:10.1111/j.1600-065X.2006.00444.x

Newman, A. M., Liu, C. L., Green, M. R., Gentles, A. J., Feng, W., Xu, Y., . . . Alizadeh, A. A. (2015). Robust enumeration of cell subsets from tissue expression profiles. *Nat Methods, 12*(5), 453-457. doi:10.1038/nmeth.3337

Robinson, D. R., Wu, Y. M., Lonigro, R. J., Vats, P., Cobain, E., Everett, J., . . . Chinnaiyan, A. M. (2017). Integrative clinical genomics of metastatic cancer. *Nature, 548*(7667), 297-303. doi:10.1038/nature23306

Roh, W., Chen, P. L., Reuben, A., Spencer, C. N., Prieto, P. A., Miller, J. P., . . . Futreal, P. A. (2017). Integrated molecular analysis of tumor biopsies on sequential CTLA-4 and PD-1 blockade reveals markers of response and resistance. *Sci Transl Med, 9*(379). doi:10.1126/scitranslmed.aah3560

Roth, A., Khattra, J., Yap, D., Wan, A., Laks, E., Biele, J., . . . Shah, S. P. (2014). PyClone: statistical inference of clonal population structure in cancer. *Nat Methods, 11*(4), 396-398. doi:10.1038/nmeth.2883

Seth, S., Li, C. Y., Ho, I. L., Corti, D., Loponte, S., Sapio, L., . . . Viale, A. (2019). Pre-existing Functional Heterogeneity of Tumorigenic Compartment as the Origin of Chemoresistance in Pancreatic Tumors. *Cell Rep, 26*(6), 1518-1532 e1519. doi:10.1016/j.celrep.2019.01.048

Shu, S., Cochran, A. J., Huang, R. R., Morton, D. L., & Maecker, H. T. (2006). Immune responses in the draining lymph nodes against cancer: implications for immunotherapy. *Cancer Metastasis Rev, 25*(2), 233-242. doi:10.1007/s10555-006-8503-7

Yoshihara, K., Shahmoradgoli, M., Martinez, E., Vegesna, R., Kim, H., Torres-Garcia, W., . . . Verhaak, R. G. (2013). Inferring tumour purity and stromal and immune cell admixture from expression data. *Nat Commun, 4*, 2612. doi:10.1038/ncomms3612

Zhang, J. (2018). CNTools: Convert segment data into a region by sample matrix to allow for other high level computational analyses.

Zheng, G., Ma, Y., Zou, Y., Yin, A., Li, W., & Dong, D. (2018). HCMDB: the human cancer metastasis database. *Nucleic Acids Res, 46*(D1), D950-D955. doi:10.1093/nar/gkx1008

**Second round of review**

**Reviewer 1**

The revised manuscript by Lee et al is improved. The authors have addressed my prior concerns. A minor comment is that for the Supplementary Fig 2, the authors might add a comment that although the genetic distances between the primary and metastases were significantly greater than within the primary, the absolute differences were relatively minor.

**Reviewer 2**

The authors addressed my comments regarding methods and have also improved the figures significantly.
